# Unveiling the silent crisis: global burden of suicide-related deaths among children aged 10–14 years

**DOI:** 10.1007/s12519-023-00781-z

**Published:** 2024-01-19

**Authors:** Irmina Maria Michalek, Pawel Koczkodaj, Marzena Michalek, Florentino Luciano Caetano dos Santos

**Affiliations:** 1 Department of Cancer Epidemiology and Primary Prevention, Maria Sklodowska–Curie National Research Institute of Oncology, ul. Wawelska 15 B, Warsaw, Poland; 2 Private Psychiatric Practice–Marzena Michalek, MD, Siedlce, Poland; 3 Harvard Business School, Harvard University, Boston, MA USA

**Keywords:** Early adolescent, Global Burden of Disease, Incidence, Suicide, Years of life lost

## Abstract

**Background:**

The rise in suicides among children aged 10–14 years demands urgent attention globally. This study aims to assess the global burden of suicide-related deaths in this age group from 1990 to 2019, considering factors such as sex, geography, and sociodemographics, to inform prevention strategies and interventions.

**Methods:**

The data from Global Burden of Disease 2019, encompassing 204 countries and territories, were analyzed to investigate deaths and years of life lost (YLLs) due to suicide among children aged 10–14 years. Statistical analyses, including mortality rates, YLLs, and the sociodemographic index (SDI), were conducted using standardized tools.

**Results:**

In 2019, a total of 8327 [95% uncertainty interval (UI) = 7073–9685] children aged 10–14 years died globally due to suicide, with a mortality rate of 1.30 (95% UI = 1.10–1.51) per 100,000. The rates varied across countries/territories ranging between 0.05 (95% UI = 0.02–0.10) in South Africa and 7.49 (95% UI = 5.13–10.57) in Greenland. The contribution of suicide-related deaths to all-cause mortality ranged from 0.07% (95% UI = 0.04%–0.15%) in South Africa to 33.02% (95% UI = 24.36%–41.53%) in Greenland. Worldwide, there were approximately 636,196 (95% UI = 540,383–740,009) YLLs due to suicide, with a rate of 99.07 (95% UI = 84.15–115.23) per 100,000. The association between SDI and suicide-related deaths was evident, with higher contributions observed in countries with higher SDI.

**Conclusions:**

This study reveals a concerning global burden of suicide-related deaths among children aged 10–14 years. Despite progress in reducing mortality rates, suicide remains a significant issue. While overall rates have declined, the percentage of deaths caused by suicide in this age group is increasing.

**Graphical abstract:**

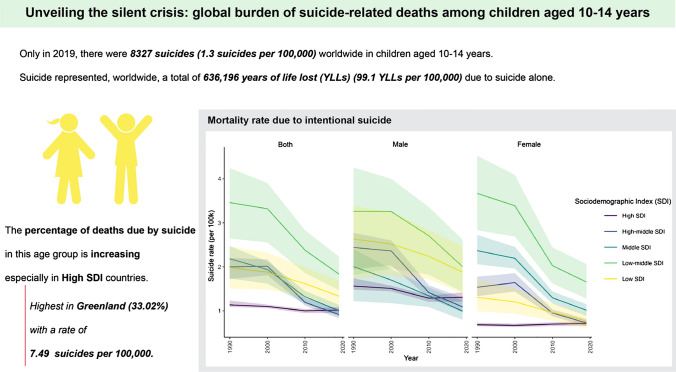

**Supplementary Information:**

The online version contains supplementary material available at 10.1007/s12519-023-00781-z.

## Introduction

In recent years, in some countries, such as the United States, there has been an alarming rise in suicides among children aged 10–14 years [1, 2], marking a distressing trend that demands immediate attention. The gravity of this issue cannot be overstated, as the loss of young people not only devastates families but also affects communities and society as a whole [3–7]. Addressing this growing concern, raising awareness, and delving deeper into the factors contributing to these tragic events is imperative.

Studying suicides among children aged 10–14 years is of predominant importance because of the unique vulnerabilities and challenges faced by this specific age group [8, 9]. Early adolescence (10–14 years) is marked by significant physical, cognitive, and emotional changes, making children particularly susceptible to various stressors and pressures [10–12]. This vulnerable period coincides with a notable increase in suicide rates, with recent research showing that adolescents aged 10–19 exhibited seasonal changes in suicides that increased from March to April, paralleling the school year [13]. Puberty’s profound influence leads to significant changes in body morphology, sexual development, and brain restructuring [8]. Psychologically, this stage is marked by susceptibility to peer influences, limited future orientation, reduced risk perception, and an increased likelihood of engaging in risk-taking behaviors, whereas identity formation and emerging interests, including sexuality and romantic relationships, become paramount [8].

The global prevalence of suicide among children aged 10–14 years is a significant concern that requires attention. As comprehensive global data on this issue are currently limited, the available statistics primarily stem from scattered sources, predominantly focused on the United States. According to reputable sources, such as the Centers for Disease Control and Prevention, thousands of children in this age group tragically take their own lives each year in the United States alone [1]. From 2003 to 2004, there was an 8% increase in the suicide rate among youth and young adults (10–24 years), with a significant upward trend in suicide rates among specific sex-age groups, particularly females aged 10–14 years [1].

These distressing figures reveal a disturbing trend that necessitates immediate attention and action. The absence of comprehensive global data underscores the urgent need for further research and surveillance to gain a holistic understanding of the global crisis. By examining the prevalence of suicide among children aged 10–14 years in various countries, we can begin to comprehend the extent of this problem and identify potential patterns and trends. Such insights are crucial for developing targeted prevention strategies and interventions to mitigate the incidence of suicide in this vulnerable population. Notably, the total number of deaths from suicide for individuals of all ages increased by 6.7% globally between 1990 and 2016, reaching 817,000 deaths in 2016 [14]. Additionally, it is important to acknowledge that globally, the ratios of suicide deaths exhibited significant variations, especially in relation to the sociodemographic index (SDI) level, emphasizing the complex nature of this public health issue.

The primary objective of this study was to calculate the global burden of disease metrics explicitly related to suicide-related deaths among children aged 10–14 years between 1990 and 2019. This study aimed to comprehensively assess the extent and impact of deaths due to suicide within this specific age group, considering factors such as sex, geographical location, and sociodemographic background. By calculating these metrics, we aimed to enhance the understanding of the magnitude and distribution of suicide deaths among children aged 10–14 years.

## Methods

### Source of the data

The data utilized in this study were obtained from the Global Burden of Disease (GBD) study, specifically from the GBD Integrated Health Metrics Evaluation Global Health Data Exchange (GHDx). The GBD is a groundbreaking initiative that provides a comprehensive and systematic assessment of mortality and disability across countries, time periods, age groups, and sexes. It quantifies the burden of diseases, injuries, and risk factors, allowing for a comprehensive understanding of health loss and enabling policymakers and healthcare systems to improve health outcomes and address health inequalities. The data used in this study were based on the GBD 2019, which provides the most up-to-date and robust health metric estimates, ensuring the reliability and validity of our analyses.

To investigate deaths and years of life lost (YLLs) due to suicide among children aged 10–14 years, we performed a query in the GHDx database. Specifically, we extracted data for the years 1990, 2000, 2010, and 2019. The queried variables included the number of deaths, the percentage of deaths attributable to suicide, and the mortality rate per 100,000 population aged 10–14 years. Additionally, we exported data for YLLs, encompassing the number of YLLs due to suicide in the same age group and the percentage of YLLs attributable to suicide. The data were exported from the GHDx database in a disaggregated format, allowing us to analyze the information by sex, countries and territories, GBD regions, and GBD super regions. This comprehensive approach enabled us to assess variations in suicide-related deaths and YLLs across different geographic locations and population subgroups.

In our study, suicide was defined as intentional acts of bodily self-harm inflicted upon oneself, leading to death, as classified by the International Classification of Diseases (ICD-9: E950–E959, ICD-10: X60–X64.9, X66–X84.9, Y87.0). This clear and standardized definition ensured the accurate identification of cases relevant to our research objectives and allowed for meaningful comparisons across different studies and populations.

### Statistical analyses

The GBD framework employed standardized tools such as the cause of death ensemble model, spatiotemporal Gaussian process regression, and DisMod-MR, described in detail elsewhere [15], to model and estimate deaths and YLLs for diverse diseases and injuries. These tools ensured the consistency and reliability of the analysis, allowing for comparisons across different locations and time periods.

The mortality rate was estimated by calculating the number of deaths per 100,000 population within a specific year, country, or geographic area, considering sex and age. YLLs were employed to quantify premature mortality by taking into account both the frequency of cause of death and age at death [16]. To study the link between demographic factors and disease burden, the SDI was utilized as a composite indicator, which incorporated income per capita, years of education, and fertility rate among females under 25 years [17]. The SDI provided insights into the impact of sociodemographic characteristics on the distribution and trends of disease burden. Furthermore, 95% uncertainty intervals (UIs) were calculated to account for the inherent variability and provide a measure of the robustness of the estimates. The statistical analyses were carried out using R (version 4.3.0-“Already Tomorrow”), ensuring a rigorous and standardized approach to data analysis.

### Compliance with ethical and reporting standards

Data for this study were sourced from the GHDx database, a publicly available pre-existing and aggregated dataset. Consequently, no informed consent was necessary. The original GBD study received approval from the University of Washington’s Human Subjects Division (Study ID: STUDY00009060). This study adheres to the guidelines set forth by the Strengthening the Reporting of Observational Studies in Epidemiology Statement: guidelines for reporting observational studies [18].

## Results

In 2019, among the global population of 642,186,704 children aged 10–14 years (331,334,185 males and 310,852,519 females), 8327 (95% UI = 7073–9685) children died due to suicide, including 4988 (95% UI = 3924–6088) male and 3338 (95% UI = 2850–3861) female deaths. The mortality rate due to suicide per 100,000 for both sexes was 1.30 (95% UI = 1.10–1.51; Table 1), for males 1.51 (95% UI = 1.18–1.84), and for females 1.07 (95% UI = 0.92–1.24; Supplementary Tables 1 and 2). The rate varied considerably, depending on the geographical location. The lowest rates were observed in South Africa (0.05; 95% UI = 0.02–0.10), Saudi Arabia (0.10; 95% UI = 0.06–0.16), Antigua and Barbuda (0.13; 95% UI = 0.09–0.18), Bahamas (0.14; 95% UI = 0.09–0.21), and Bermuda (0.15; 95% UI = 0.10–0.21). The highest values were reported in Greenland (7.49; 95% UI = 5.13–10.57), Mongolia (4.61; 95% UI = 2.27–7.19), Kiribati (4.40; 95% UI = 2.61–6.76), Uzbekistan (4.16; 95% UI = 3.04–5.55), and Ecuador (3.99; 95% UI = 2.21–5.55).

**Table 1:** Deaths due to suicide among children aged 10–14 years

Location	Death rate 1990 (95% UI)	Death rate 2019 (95% UI)	Change (95% UI)	Death percentage 1990 (95% UI), %	Death percentage 2019 (95% UI), %	Change (95% UI), %	YLLs 1990 (95% UI)	YLLs 2019 (95% UI)	Change (95% UI)
Global	2.33 (1.90–2.62)	1.30 (1.10–1.51)	− 1.03 (− 1.04 to − 1.02)	2.76 (2.25–3.09)	2.78 (2.38–3.15)	0.02 (0.01–0.03)	955,590 (778,870–1,076,050)	636,196 (540,383–740,009)	− 319,394 (− 319,780 to − 319,008)
Central Europe, Eastern Europe, and Central Asia	2.40 (2.30–2.51)	1.99 (1.76–2.32)	− 0.41 (− 0.44 to − 0.39)	5.65 (5.43–5.89)	7.77 (7.07–8.87)	2.12 (2.05–2.19)	62,399 (59,829–65,273)	39,026 (34,492–45,508)	− 23,372 (− 23,431 to − 23,314)
Central Asia	2.52 (2.26–2.79)	2.93 (2.40–3.63)	0.41 (0.32–0.50)	4.65 (4.19–5.14)	8.07 (6.78–9.69)	3.42 (3.24–3.60)	13,954 (12,499–15,429)	17,930 (14,648−22,179)	3976 (3916–4036)
Armenia	0.32 (0.23–0.43)	0.59 (0.40–0.87)	0.27 (− 0.21 to 0.75)	0.90 (0.67–1.22)	3.18 (2.22–4.63)	2.28 (0.38–4.18)	74 (54–101)	85 (57–126)	11 (2–20)
Azerbaijan	0.40 (0.25–0.84)	0.47 (0.27–0.77)	0.07 (− 0.36 to 0.50)	0.78 (0.49–1.60)	1.33 (0.78–2.19)	0.56 (− 0.17 to 1.29)	218 (138–459)	266 (154–437)	48 (21–75)
Georgia	0.59 (0.44–0.78)	0.47 (0.32–0.66)	− 0.12 (− 0.51 to 0.27)	1.24 (0.94–1.62)	2.15 (1.51–2.97)	0.92 (− 0.33 to 2.16)	198 (149–263)	78 (54–110)	− 120 (− 130 to − 110)
Kazakhstan	4.50 (3.73–5.33)	3.73 (2.83–4.83)	− 0.77 (− 1.09 to − 0.44)	8.26 (6.95–9.79)	12.44 (9.59–15.82)	4.18 (3.39–4.97)	5493 (4550–6501)	4179 (3172–5413)	− 1314 (− 1356 to − 1271)
Kyrgyzstan	4.17 (3.34–5.02)	2.96 (2.21–3.82)	− 1.21 (− 1.73 to − 0.69)	6.78 (5.45–8.16)	10.71 (8.29–13.78)	3.93 (2.68–5.18)	1539 (1233–1850)	1356 (1013–1750)	− 182 (− 207 to − 157)
Mongolia	6.38 (3.44–9.53)	4.61 (2.27–7.19)	− 1.77 (− 3.77 to 0.22)	9.19 (5.00–13.84)	11.87 (5.94–16.95)	2.68 (− 0.32 to 5.69)	1301 (701–1943)	937 (461–1462)	− 364 (− 411 to − 318)
Tajikistan	1.07 (0.78–1.44)	0.94 (0.59–1.41)	− 0.13 (− 0.50 to 0.24)	1.90 (1.39–2.58)	2.57 (1.68–3.71)	0.68 (0.01–1.35)	507 (369–687)	680 (426–1022)	173 (147 to 200)
Turkmenistan	1.70 (1.31–2.22)	1.24 (0.86–1.74)	− 0.46 (− 0.95 to 0.04)	3.18 (2.48–4.12)	3.35 (2.37–4.66)	0.17 (− 0.79 to 1.14)	555 (426–723)	422 (292–591)	− 133 (− 152 to − 114)
Uzbekistan	2.24 (1.85–2.70)	4.16 (3.04–5.55)	1.92 (1.68–2.16)	4.07 (3.41–4.89)	9.67 (7.29–12.46)	5.59 (5.17–6.02)	4070 (3376–4921)	9927 (7271–13,266)	5857 (5793–5920)
Central Europe	1.66 (1.54–1.81)	0.81 (0.69–0.96)	− 0.85 (− 0.89 to − 0.81)	5.16 (4.79–5.61)	5.84 (5.19–6.69)	0.68 (0.48–0.88)	13,150 (12,169–14,286)	3762 (3190–4426)	− 9388 (− 9415 to − 9361)
Albania	0.91 (0.61–1.31)	1.30 (0.72–2.05)	0.39 (− 0.60 to 1.38)	2.03 (1.38–2.92)	4.33 (2.43–6.91)	2.29 (− 0.25 to 4.84)	247 (166–356)	155 (86–245)	− 91 (− 109 to − 74)
Bosnia and Herzegovina	2.04 (1.15–2.75)	1.12 (0.66–1.73)	− 0.92 (− 1.87 to 0.03)	8.13 (4.58–11.05)	7.89 (4.90–11.79)	− 0.23 (− 4.67 to 4.20)	580 (328–785)	143 (85–221)	− 437 (− 459 to − 415)
Bulgaria	3.30 (2.71–3.94)	1.03 (0.68–1.52)	− 2.27 (− 2.78 to − 1.76)	7.56 (6.21–9.00)	4.97 (3.48–6.80)	− 2.59 (− 4.11 to − 1.06)	1606 (1315–1912)	270 (177–396)	− 1336 (− 1356 to − 1317)
Croatia	1.89 (1.48–2.38)	0.78 (0.53–1.12)	− 1.12 (− 1.68 to − 0.55)	8.21 (6.41–10.32)	7.37 (5.19–10.07)	− 0.84 (− 4.19 to 2.51)	504 (392–633)	129 (87–185)	− 375 (− 389 to − 362)
Czechia	1.65 (1.29–2.05)	0.52 (0.35–0.75)	− 1.13 (− 1.44 to − 0.83)	6.97 (5.47–8.63)	5.92 (4.20–8.22)	− 1.05 (− 3.08 to 0.99)	1082 (847–1343)	226 (154–329)	− 856 (− 875 to − 838)
Hungary	1.78 (1.44–2.20)	0.59 (0.40–0.86)	− 1.19 (− 1.52 to − 0.87)	6.96 (5.62–8.54)	5.01 (3.54–6.95)	− 1.95 (− 3.70 to − 0.20)	1164 (941–1433)	222 (150–323)	− 942 (− 960 to − 923)
Montenegro	1.90 (1.27–2.75)	1.17 (0.65–1.86)	− 0.74 (− 3.02 to 1.55)	8.50 (5.72–12.16)	8.46 (5.02–13.12)	− 0.04 (− 10.78 to 10.69)	79 (52–114)	34 (19–54)	− 45 (− 54 to − 36)
North Macedonia	1.47 (1.03–2.11)	0.84 (0.52–1.26)	− 0.63 (− 1.61 to 0.35)	4.90 (3.41–6.98)	5.07 (3.34–7.32)	0.17 (− 3.54 to 3.88)	201 (140–288)	74 (46–111)	− 127 (− 140 to − 115)
Poland	1.67 (1.53–1.82)	0.86 (0.72–1.05)	− 0.81 (− 0.89 to − 0.72)	5.93 (5.42–6.46)	7.64 (6.73–9.07)	1.71 (1.18–2.23)	4156 (3805–4523)	1300 (1079–1574)	− 2856 (− 2873 to − 2839)
Romania	1.14 (0.90–1.40)	0.98 (0.69–1.34)	− 0.15 (− 0.37 to 0.06)	2.53 (2.02–3.11)	4.78 (3.48–6.31)	2.26 (1.51–3.01)	1784 (1421–2197)	825 (582–1126)	− 959 (− 985 to − 933)
Serbia	1.89 (1.14–2.97)	0.42 (0.23–0.73)	− 1.48 (− 2.06 to − 0.90)	5.79 (3.50–8.99)	4.27 (2.48–7.35)	− 1.52 (− 4.24 to 1.20)	1087 (656–1708)	163 (91–285)	− 924 (− 959 to − 890)
Slovakia	1.18 (0.77–1.75)	0.60 (0.35–0.95)	− 0.58 (− 1.18 to 0.03)	4.65 (3.05–6.91)	4.62 (2.86–6.99)	− 0.04 (− 2.85 to 2.77)	426 (280–630)	128 (75–202)	− 297 (− 317 to − 277)
Slovenia	2.03 (1.53–2.61)	1.21 (0.81–1.70)	− 0.82 (− 1.82 to 0.17)	10.49 (8.14–13.04)	11.12 (7.63–15.31)	0.63 (− 5.63 to 6.89)	234 (176–301)	92 (62–130)	− 142 (− 152 to − 131)
Eastern Europe	2.80 (2.68–2.94)	1.95 (1.72–2.24)	− 0.86 (− 0.89 to − 0.82)	6.43 (6.14–6.72)	8.04 (7.36–9.07)	1.62 (1.51–1.72)	35,294 (33,685–36,983)	17,334 (15,337–19,919)	− 17,960 (− 17,998 to − 17,922)
Belarus	2.28 (1.81–2.79)	1.21 (0.78–1.77)	− 1.07 (− 1.53 to − 0.61)	6.00 (4.83–7.35)	7.46 (5.04–10.27)	1.45 (− 0.35 to 3.25)	1332 (1058–1630)	465 (298–680)	− 867 (− 890 to − 844)
Estonia	3.88 (2.91–5.08)	0.95 (0.63–1.38)	− 2.94 (− 4.29 to − 1.58)	8.40 (6.30–11.01)	7.77 (5.27–11.02)	− 0.63 (− 6.60 to 5.33)	330 (247–432)	52 (35–76)	− 278 (− 289 to − 267)
Latvia	2.72 (2.09–3.43)	0.80 (0.52–1.19)	− 1.91 (− 2.87 to − 0.96)	5.31 (4.06–6.65)	5.41 (3.66–7.77)	0.11 (− 3.74 to 3.95)	359 (277–454)	61 (40–91)	− 298 (− 309 to − 286)
Lithuania	2.37 (1.87–2.97)	1.65 (1.17–2.27)	− 0.72 (− 1.57 to 0.13)	6.18 (4.90–7.74)	9.22 (6.80–12.46)	3.04 (− 0.33 to 6.41)	476 (377–597)	164 (117–226)	− 312 (− 325 to − 299)
Republic of Moldova	1.96 (1.52–2.46)	1.24 (0.85–1.74)	− 0.72 (− 1.36 to − 0.08)	4.15 (3.23–5.19)	5.20 (3.69–7.29)	1.05 (− 0.92 to 3.02)	577 (446–723)	194 (132–272)	− 382 (− 397 to − 367)
Russian Federation	3.12 (2.99–3.25)	1.91 (1.67–2.24)	− 1.21 (− 1.26 to − 1.16)	6.98 (6.69–7.28)	8.30 (7.59–9.61)	1.32 (1.17–1.47)	26,316 (25,250–27,467)	12,111 (10,592–14,211)	− 14,206 (− 14,240 to − 14,171)
Ukraine	2.07 (1.72–2.48)	2.41 (1.77–3.19)	0.34 (0.13–0.54)	5.02 (4.23–5.99)	7.66 (5.72–10.04)	2.63 (2.11–3.16)	5905 (4916–7063)	4286 (3150–5669)	− 1618 (− 1665 to − 1572)
High-income	1.09 (1.05–1.18)	1.04 (0.97–1.11)	− 0.05 (− 0.06 to − 0.04)	4.64 (4.45–5.02)	9.04 (8.51–9.66)	4.40 (4.35–4.45)	52,734 (50,602–57,028)	49,153 (46,068–52,560)	− 3581 (− 3621 to − 3541)
Australasia	0.92 (0.77–1.10)	0.83 (0.67–1.03)	− 0.09 (− 0.21 to 0.03)	4.72 (3.96–5.59)	8.68 (6.95–10.65)	3.96 (3.04–4.89)	1070 (897–1270)	1161 (929–1430)	91 (73–109)
Australia	0.77 (0.61–0.97)	0.74 (0.56–0.96)	− 0.03 (− 0.19 to 0.13)	4.13 (3.26–5.20)	8.05 (6.09–10.50)	3.92 (2.66–5.17)	736 (581–924)	860 (648–1118)	124 (104–144)
New Zealand	1.66 (1.35–2.02)	1.30 (1.04–1.60)	− 0.35 (− 0.77 to 0.07)	6.86 (5.57–8.35)	11.21 (8.96–13.79)	4.34 (1.89–6.79)	334 (271–407)	301 (241–370)	− 33 (− 44 to − 23)
High-income Asia Pacific	0.99 (0.86–1.38)	1.00 (0.88–1.12)	0.01 (− 0.04 to 0.06)	4.25 (3.73–5.93)	12.59 (11.10–14.07)	8.34 (8.00–8.69)	9863 (8644–13,771)	6158 (5461–6908)	− 3705 (− 3758 to − 3651)
Brunei Darussalam	0.47 (0.26–0.81)	0.37 (0.23–0.57)	− 0.10 (− 1.92 to 1.72)	1.01 (0.57–1.74)	1.50 (0.95–2.24)	0.49 (− 3.39 to 4.37)	9 (5–16)	9 (6–14)	0 (− 5 to 4)
Japan	0.79 (0.74–0.84)	0.98 (0.91–1.06)	0.20 (0.17–0.22)	5.52 (5.23–5.86)	13.31 (12.43–14.29)	7.79 (7.54–8.04)	5253 (4977–5582)	4144 (3853–4453)	− 1109 (− 1121 to − 1097)
Republic of Korea	1.40 (1.05–2.54)	1.07 (0.70–1.44)	− 0.33 (− 0.58 to − 0.09)	3.32 (2.49–5.99)	12.22 (8.13–16.53)	8.90 (7.44–10.37)	4382 (3278–7953)	1887 (1246–2545)	− 2495 (− 2570 to − 2420)
Singapore	1.26 (0.98–1.58)	0.71 (0.54–0.95)	− 0.55 (− 1.02 to − 0.07)	5.64 (4.41–7.10)	6.86 (5.20–9.02)	1.22 (− 1.74 to 4.18)	218 (170–274)	118 (89–157)	− 100 (− 109 to − 90)
High-income North America	1.63 (1.57–1.70)	1.63 (1.53–1.74)	0.00 (− 0.01 to 0.01)	6.45 (6.19–6.73)	11.73 (11.00–12.52)	5.28 (5.20–5.36)	24,208 (23,198–25,261)	29,253 (27,412–31,232)	5046 (5020–5071)
Canada	1.87 (1.57–2.20)	1.68 (1.32–2.07)	− 0.19 (− 0.36 to − 0.03)	8.49 (7.15–9.95)	14.69 (11.59–18.14)	6.20 (5.13–7.26)	2703 (2270–3173)	2681 (2113–3307)	− 22 (− 50 to 6)
Greenland	16.45 (11.62–21.59)	7.49 (5.13–10.57)	− 8.95 (− 24.73 to 6.82)	29.96 (20.84–38.52)	33.02 (24.36–41.53)	3.06 (− 29.57 to 35.68)	49 (35–65)	22 (15–30)	− 28 (− 33 to − 22)
United States of America	1.60 (1.54–1.67)	1.63 (1.52–1.74)	0.02 (0.01–0.04)	6.25 (6.01–6.53)	11.49 (10.79–12.27)	5.24 (5.16–5.32)	21,455 (20,611–22,391)	26,550 (24,861–28,375)	5095 (5071–5119)
Southern Latin America	1.08 (0.94–1.22)	1.37 (1.10–1.70)	0.30 (0.21–0.38)	3.17 (2.76–3.61)	6.63 (5.33–8.22)	3.46 (3.13–3.79)	3932 (3416–4467)	5171 (4150–6410)	1240 (1205–1275)
Argentina	0.99 (0.82–1.18)	1.48 (1.13–1.91)	0.49 (0.37–0.62)	2.84 (2.35–3.40)	6.57 (4.98–8.46)	3.72 (3.27–4.17)	2490 (2058–2978)	3930 (3004–5078)	1441 (1404–1478)
Chile	1.30 (1.03–1.61)	1.10 (0.82–1.41)	− 0.20 (− 0.41 to 0.02)	4.03 (3.21–5.04)	6.88 (5.20–8.88)	2.85 (1.89–3.81)	1192 (948–1485)	1022 (763–1314)	− 170 (− 193 to − 147)
Uruguay	1.19 (0.89–1.56)	1.21 (0.86–1.63)	0.02 (− 0.55 to 0.59)	3.66 (2.75–4.80)	6.65 (4.80–8.89)	2.99 (0.73–5.25)	249 (187–327)	219 (155–294)	− 31 (− 43 to − 18)
Western Europe	0.73 (0.68–0.78)	0.41 (0.37–0.47)	− 0.32 (− 0.33 to − 0.30)	3.57 (3.34–3.84)	4.81 (4.32–5.43)	1.24 (1.13–1.35)	13,662 (12,753–14,646)	7409 (6654–8389)	− 6253 (− 6278 to − 6228)
Andorra	0.53 (0.32–0.85)	0.24 (0.15–0.36)	− 0.30 (− 4.51 to 3.92)	3.70 (2.36–5.69)	3.29 (2.20–4.79)	− 0.41 (− 28.19 to 27.38)	1 (1–2)	1 (0–1)	− 1 (− 2 to 1)
Austria	1.18 (0.93–1.47)	0.61 (0.44–0.80)	− 0.57 (− 0.89 to − 0.25)	7.28 (5.72–9.07)	7.00 (5.13–9.11)	− 0.27 (− 2.76 to 2.22)	392 (309–489)	197 (143–258)	− 195 (− 207 to − 183)
Belgium	1.10 (0.87–1.38)	0.67 (0.47–0.91)	− 0.43 (− 0.71 to − 0.15)	5.44 (4.30–6.79)	7.89 (5.57–10.59)	2.46 (0.26–4.65)	512 (405–640)	335 (235–451)	− 177 (− 192 to − 161)
Cyprus	0.32 (0.18–0.53)	0.18 (0.11–0.29)	− 0.14 (− 1.05 to 0.77)	2.03 (1.12–3.35)	1.77 (1.09–2.75)	− 0.26 (− 5.46 to 4.95)	16 (9–26)	9 (6–15)	− 7 (− 12 to − 1)
Denmark	0.97 (0.72–1.28)	0.25 (0.15–0.37)	− 0.72 (− 1.11 to − 0.33)	5.14 (3.82–6.81)	3.72 (2.28–5.51)	− 1.42 (− 4.58 to 1.74)	240 (178–317)	64 (40–96)	− 176 (− 187 to − 165)
Finland	1.44 (1.11–1.82)	0.66 (0.48–0.88)	− 0.79 (− 1.21 to − 0.36)	7.68 (5.93–9.71)	7.11 (5.25–9.47)	− 0.56 (− 3.49 to 2.36)	358 (277–452)	154 (114–208)	− 204 (− 215 to − 192)
France	0.79 (0.65–0.96)	0.53 (0.39–0.73)	− 0.26 (− 0.35 to − 0.17)	4.08 (3.34–4.94)	6.45 (4.72–8.74)	2.36 (1.60–3.13)	2324 (1901–2815)	1686 (1227–2288)	− 638 (− 669 to − 606)
Germany	0.96 (0.80–1.15)	0.49 (0.37–0.65)	− 0.47 (− 0.55 to − 0.38)	5.09 (4.23–6.09)	5.91 (4.38–7.73)	0.81 (0.11–1.52)	3059 (2539–3667)	1440 (1069–1895)	− 1619 (− 1648 to − 1590)
Greece	0.30 (0.22–0.40)	0.18 (0.12–0.26)	− 0.12 (− 0.29 to 0.06)	1.63 (1.21–2.17)	1.85 (1.27–2.60)	0.22 (− 1.02 to 1.45)	174 (129–232)	73 (50–104)	− 102 (− 111 to − 92)
Iceland	1.03 (0.74–1.37)	0.42 (0.28–0.61)	− 0.60 (− 2.30 to 1.10)	5.88 (4.25–7.77)	5.59 (3.89–7.79)	− 0.29 (− 12.45 to 11.86)	16 (12–22)	7 (5–11)	− 9 (− 12 to − 6)
Ireland	0.72 (0.54–0.94)	0.52 (0.37–0.74)	− 0.20 (− 0.57 to 0.17)	3.89 (2.94–5.14)	6.64 (4.71–9.49)	2.75 (− 0.42 to 5.92)	194 (146–255)	136 (97–194)	− 58 (− 69 to − 46)
Israel	0.57 (0.44–0.73)	0.40 (0.29–0.54)	− 0.17 (− 0.40 to 0.05)	3.38 (2.60–4.32)	4.07 (2.90–5.61)	0.69 (− 0.88 to 2.25)	217 (167–278)	236 (170–323)	18 (6–31)
Italy	0.45 (0.41–0.49)	0.25 (0.23–0.29)	− 0.20 (− 0.23 to − 0.17)	2.27 (2.09–2.46)	2.95 (2.61–3.41)	0.67 (0.39–0.96)	1223 (1122–1325)	556 (494–640)	− 667 (− 675 to − 659)
Luxembourg	0.85 (0.63–1.13)	0.38 (0.26–0.58)	− 0.47 (− 1.94 to 1.00)	4.40 (3.27–5.88)	4.69 (3.30–6.60)	0.29 (− 8.77 to 9.34)	14 (10–18)	10 (6–15)	− 4 (− 7 to − 1)
Malta	0.23 (0.16–0.31)	0.27 (0.18–0.40)	0.04 (− 1.04 to 1.13)	1.95 (1.35–2.66)	2.39 (1.58–3.44)	0.44 (− 7.27 to 8.15)	5 (4–7)	4 (3–6)	− 1 (− 3 to 1)
Monaco	0.64 (0.40–0.99)	0.40 (0.26–0.61)	− 0.24 (− 8.06 to 7.58)	4.17 (2.70–6.08)	4.34 (2.85–6.34)	0.16 (− 45.69 to 46.01)	1 (0–1)	1 (0–1)	0 (− 1 to 1)
The Netherlands	0.79 (0.61–0.98)	0.48 (0.35–0.64)	− 0.31 (− 0.50 to − 0.12)	3.85 (2.99–4.81)	5.63 (4.11–7.52)	1.78 (0.29–3.26)	542 (422–675)	345 (253–465)	− 196 (− 212 to − 181)
Norway	2.59 (2.37–2.79)	0.96 (0.85–1.07)	− 1.63 (− 1.83 to − 1.43)	13.17 (12.15–14.18)	11.79 (10.53–13.16)	− 1.37 (− 2.94 to 0.20)	523 (480–563)	233 (207–260)	− 290 (− 295 to − 285)
Portugal	0.83 (0.65–1.06)	0.23 (0.16–0.32)	− 0.60 (− 0.81 to − 0.40)	2.22 (1.73–2.82)	2.54 (1.78–3.47)	0.31 (− 1.02 to 1.65)	535 (417–682)	93 (65–127)	− 442 (− 455 to − 429)
San Marino	0.77 (0.47–1.18)	0.44 (0.28–0.67)	− 0.33 (− 7.75 to 7.09)	4.68 (2.87–7.04)	4.59 (3.09–6.52)	− 0.10 (− 41.48 to 41.28)	1 (1–2)	1 (0–1)	0 (− 1 to 1)
Spain	0.74 (0.60–0.91)	0.31 (0.22–0.41)	− 0.43 (− 0.52 to − 0.34)	3.11 (2.50–3.84)	3.57 (2.58–4.77)	0.46 (− 0.26 to 1.18)	1811 (1458–2229)	578 (419–773)	− 1232 (− 1255 to − 1209)
Sweden	0.91 (0.73–1.12)	0.62 (0.49–0.76)	− 0.29 (− 0.52 to − 0.06)	5.68 (4.61–7.03)	7.77 (6.14–9.51)	2.09 (0.25–3.93)	341 (277–424)	283 (224–348)	− 58 (− 69 to − 48)
Switzerland	1.16 (0.91–1.47)	0.61 (0.44–0.81)	− 0.55 (− 0.90 to − 0.20)	6.11 (4.78–7.73)	7.15 (5.20–9.64)	1.04 (− 1.61 to 3.69)	331 (259–420)	196 (142–262)	− 135 (− 147 to − 123)
United Kingdom	0.31 (0.29–0.33)	0.25 (0.23–0.27)	− 0.06 (− 0.08 to − 0.04)	1.59 (1.49–1.71)	2.90 (2.69–3.14)	1.30 (1.16–1.45)	822 (767–880)	764 (710–830)	− 57 (− 63 to − 51)
Latin America and Caribbean	0.89 (0.83–0.96)	1.24 (1.09–1.42)	0.35 (0.33–0.36)	1.49 (1.39–1.61)	3.66 (3.39–4.01)	2.17 (2.15–2.20)	31,236 (29,051–33,926)	45,817 (40,247–52,477)	14,581 (14,519–14,644)
Andean Latin America	1.12 (0.90–1.50)	1.73 (1.12–2.29)	0.61 (0.46–0.75)	1.37 (1.10–1.84)	4.90 (3.30–6.19)	3.53 (3.26–3.79)	3971 (3176–5305)	7530 (4898–9977)	3559 (3492–3625)
Bolivia (Plurinational State of)	1.77 (1.14–2.61)	1.49 (0.92–2.25)	− 0.28 (− 0.78 to 0.22)	1.90 (1.22–2.72)	3.94 (2.49–5.79)	2.04 (1.32–2.77)	1070 (690–1576)	1261 (779–1901)	191 (150–232)
Ecuador	1.49 (1.13–1.97)	3.99 (2.21–5.55)	2.51 (2.06–2.95)	2.08 (1.59–2.78)	9.68 (5.36–12.96)	7.59 (6.78–8.41)	1393 (1063–1844)	5036 (2785–7005)	3643 (3581–3705)
Peru	0.76 (0.52–1.15)	0.55 (0.34–0.82)	− 0.21 (− 0.39 to − 0.02)	0.91 (0.64–1.41)	1.76 (1.21–2.50)	0.86 (0.57–1.15)	1508 (1028–2302)	1233 (753–1844)	− 276 (− 320 to − 231)
Caribbean	1.41 (1.17–1.73)	0.99 (0.72–1.34)	− 0.42 (− 0.55 to − 0.30)	2.23 (1.84–2.70)	1.90 (1.43–2.51)	− 0.32 (− 0.51 to − 0.14)	3824 (3177–4687)	2870 (2098–3893)	− 954 (− 994 to − 913)
Antigua and Barbuda	0.15 (0.11–0.19)	0.13 (0.09–0.18)	− 0.01 (− 1.38 to 1.35)	0.55 (0.41–0.72)	0.58 (0.42–0.79)	0.03 (− 4.40 to 4.46)	1 (0–1)	1 (0–1)	0 (− 1 to 1)
Bahamas	0.17 (0.12–0.24)	0.14 (0.09–0.21)	− 0.03 (− 0.79 to 0.73)	0.49 (0.36–0.67)	0.34 (0.23–0.49)	− 0.15 (− 1.74 to 1.43)	3 (3–5)	3 (2–5)	0 (− 2 to 2)
Barbados	0.46 (0.34–0.61)	0.25 (0.16–0.37)	− 0.21 (− 1.46 to 1.03)	1.11 (0.84–1.48)	1.04 (0.72–1.47)	− 0.07 (− 3.38–3.23)	8 (6–10)	4 (2–5)	− 4 (− 6 to − 2)
Belize	0.66 (0.48–0.89)	0.39 (0.27–0.56)	− 0.28 (− 1.48 to 0.93)	1.45 (1.06–1.92)	1.31 (0.93–1.86)	− 0.15 (− 2.72 to 2.43)	12 (9–17)	13 (9–19)	1 (− 3 to 4)
Bermuda	0.31 (0.23–0.43)	0.15 (0.10–0.21)	− 0.16 (− 2.57 to 2.24)	1.57 (1.15–2.12)	1.28 (0.93–1.77)	− 0.28 (− 12.74 to 12.18)	1 (1–1)	0 (0–1)	− 1 (− 1 to 0)
Cuba	1.80 (1.43–2.16)	0.65 (0.46–0.90)	− 1.15 (− 1.44 to − 0.86)	4.38 (3.48–5.23)	3.34 (2.47–4.47)	− 1.03 (− 1.98 to − 0.09)	1099 (875–1317)	295 (207–407)	− 804 (− 822 to − 787)
Dominica	0.40 (0.28–0.57)	0.42 (0.26–0.66)	0.02 (− 2.99 to 3.02)	0.81 (0.56–1.13)	0.79 (0.53–1.17)	− 0.01 (− 4.23 to 4.20)	3 (2–4)	2 (1–3)	− 1 (− 2 to 1)
Dominican Republic	0.62 (0.43–0.90)	0.54 (0.32–0.83)	− 0.08 (− 0.38 to 0.22)	1.21 (0.86–1.73)	1.42 (0.90–2.09)	0.21 (− 0.31 to 0.73)	397 (276–571)	393 (233–605)	− 5 (− 28 to 19)
Grenada	0.46 (0.33–0.63)	0.26 (0.18–0.36)	− 0.20 (− 2.07 to 1.66)	1.10 (0.79–1.47)	0.97 (0.68–1.35)	− 0.13 (− 4.85 to 4.59)	4 (3–5)	2 (1–2)	− 2 (− 3 to − 1)
Guyana	3.52 (2.52–4.64)	2.47 (1.67–3.50)	− 1.05 (− 2.85 to 0.76)	4.47 (3.30–5.83)	4.67 (3.27–6.27)	0.20 (− 2.04 to 2.44)	248 (177–326)	130 (87–184)	− 118 (− 131 to − 106)
Haiti	2.25 (1.35–3.46)	1.61 (0.96–2.52)	− 0.64 (− 1.23 to − 0.04)	1.70 (1.00–2.60)	1.76 (1.06–2.66)	0.06 (− 0.35 to 0.47)	1320 (792–2031)	1648 (986–2574)	328 (277–378)
Jamaica	0.20 (0.15–0.27)	0.33 (0.22–0.48)	0.12 (− 0.21 to 0.46)	0.60 (0.44–0.80)	1.49 (1.04–2.05)	0.89 (− 0.15 to 1.92)	43 (31–57)	56 (38–83)	14 (7–21)
Puerto Rico	0.54 (0.39–0.72)	0.22 (0.14–0.33)	− 0.32 (− 0.67 to 0.03)	2.26 (1.65–3.04)	2.17 (1.50–3.13)	− 0.09 (− 2.20 to 2.03)	144 (105–192)	35 (23–53)	− 109 (− 117 to − 100)
Saint Kitts and Nevis	0.50 (0.37–0.66)	0.22 (0.15–0.33)	− 0.28 (− 2.92 to 2.35)	0.92 (0.68–1.20)	0.75 (0.50–1.07)	− 0.17 (− 5.65 to 5.32)	2 (1–2)	1 (0–1)	− 1 (− 2 to 0)
Saint Lucia	0.44 (0.31–0.61)	0.36 (0.24–0.52)	− 0.08 (− 1.82 to 1.65)	1.30 (0.94–1.76)	1.26 (0.87–1.79)	− 0.04 (− 4.45 to 4.38)	6 (4–8)	3 (2–5)	− 2 (− 4 to 0)
Saint Vincent and the Grenadines	0.50 (0.35–0.69)	0.40 (0.28–0.57)	− 0.10 (− 2.07 to 1.86)	1.13 (0.80–1.54)	1.02 (0.72–1.42)	− 0.11 (− 3.94 to 3.71)	5 (4–7)	3 (2–4)	− 3 (− 5 to − 1)
Suriname	3.32 (1.66–4.74)	2.39 (1.57–3.51)	− 0.94 (− 4.06 to 2.19)	5.46 (2.75–7.75)	5.17 (3.55–7.35)	− 0.29 (− 4.80 to 4.22)	103 (52–147)	91 (60–134)	− 12 (− 24 to 0)
Trinidad and Tobago	3.01 (2.28–3.86)	1.28 (0.86–1.83)	− 1.73 (− 2.90 to − 0.56)	5.82 (4.41–7.42)	4.66 (3.39–6.33)	− 1.16 (− 3.72 to 1.41)	291 (221–374)	93 (62–132)	− 199 (− 210 to − 187)
United States Virgin Islands	0.83 (0.54–1.24)	0.26 (0.17–0.41)	− 0.56 (− 3.46 to 2.33)	1.52 (1.03–2.24)	1.34 (0.88–2.06)	− 0.19 (− 7.77 to 7.39)	7 (4–10)	1 (1–2)	− 5 (− 8 to − 3)
Central Latin America	0.90 (0.83–0.97)	1.40 (1.19–1.63)	0.50 (0.47–0.53)	1.52 (1.41–1.63)	4.43 (4.05–4.93)	2.91 (2.86–2.96)	13,803 (12,816–14,907)	23,529 (20,053–27,463)	9726 (9676–9777)
Colombia	0.92 (0.75–1.11)	1.64 (1.14–2.27)	0.72 (0.57–0.88)	1.56 (1.28–1.87)	5.54 (4.12–7.40)	3.98 (3.62–4.34)	2530 (2069–3066)	4660 (3227–6443)	2130 (2080–2180)
Costa Rica	0.98 (0.76–1.27)	1.07 (0.75–1.52)	0.09 (− 0.39 to 0.56)	3.27 (2.50–4.19)	5.10 (3.82–6.74)	1.83 (0.37–3.29)	255 (196–328)	294 (205–419)	39 (25–54)
El Salvador	2.84 (2.09–3.70)	1.38 (0.87–2.01)	− 1.47 (− 2.01 to − 0.93)	3.58 (2.62–4.67)	4.11 (2.80–5.80)	0.53 (− 0.44 to 1.50)	1496 (1102–1947)	583 (368–853)	− 913 (− 942 to − 884)
Guatemala	0.61 (0.44–0.80)	0.76 (0.53–1.09)	0.15 (− 0.05 to 0.35)	0.45 (0.33–0.59)	1.67 (1.23–2.21)	1.21 (0.98–1.44)	476 (343–623)	1097 (768–1573)	622 (595–649)
Honduras	0.77 (0.46–1.35)	0.19 (0.10–0.46)	− 0.58 (− 1.04 to − 0.11)	0.82 (0.49–1.50)	0.60 (0.32–1.44)	− 0.23 (− 0.83 to 0.37)	378 (225–663)	158 (81–378)	− 219 (− 251 to − 187)
Mexico	0.70 (0.64–0.76)	1.65 (1.45–1.87)	0.96 (0.93–0.99)	1.32 (1.23–1.43)	5.59 (5.19–6.05)	4.26 (4.20–4.33)	5722 (5261–6216)	13,907 (12,160–15,686)	8184 (8153–8216)
Nicaragua	2.13 (1.54–2.79)	1.54 (1.08–2.11)	− 0.59 (− 1.07 to − 0.11)	4.63 (3.35–6.00)	5.88 (4.33–7.81)	1.25 (0.12–2.39)	869 (629–1138)	776 (547–1062)	− 93 (− 118 to − 68)
Panama	0.90 (0.65–1.23)	0.79 (0.54–1.12)	− 0.11 (− 0.61 to 0.38)	2.12 (1.56–2.86)	2.73 (1.97–3.77)	0.61 (− 0.50 to 1.73)	183 (133–250)	221 (153–316)	38 (24–52)
Venezuela (Bolivarian Republic of)	1.12 (0.91–1.37)	1.00 (0.67–1.44)	− 0.12 (− 0.30 to 0.06)	2.28 (1.85–2.80)	2.85 (2.05–3.91)	0.58 (0.22–0.93)	1894 (1540–2320)	1833 (1231–2639)	− 61 (− 98 to − 24)
Tropical Latin America	0.71 (0.65–0.77)	0.92 (0.81–1.03)	0.21 (0.19–0.23)	1.33 (1.22–1.43)	2.86 (2.59–3.17)	1.54 (1.49–1.58)	9638 (8785–10,405)	11,888 (10,487–13,398)	2250 (2219–2281)
Brazil	0.69 (0.63–0.75)	0.88 (0.77–0.99)	0.19 (0.17–0.21)	1.28 (1.17–1.39)	2.74 (2.46–3.03)	1.45 (1.40–1.50)	9157 (8299–9923)	10,950 (9649–12,312)	1794 (1764–1824)
Paraguay	1.30 (0.92–1.72)	1.85 (1.19–2.67)	0.55 (0.03–1.06)	3.52 (2.52–4.64)	6.20 (4.28–8.38)	2.68 (1.48–3.87)	481 (341–636)	938 (603–1356)	457 (429–484)
North Africa and Middle East	1.19 (0.80–1.47)	0.50 (0.38–0.63)	− 0.69 (− 0.73 to − 0.66)	1.32 (0.89–1.63)	1.24 (0.98–1.47)	− 0.08 (− 0.12 to − 0.05)	38,565 (25,820–47,722)	21,437 (16,502–27,128)	− 17,128 (− 17,258 to − 16,998)
Afghanistan	0.87 (0.26–1.51)	0.48 (0.18–0.85)	− 0.39 (− 0.77 to − 0.01)	0.58 (0.18–1.02)	0.67 (0.25–1.15)	0.09 (− 0.14 to 0.31)	924 (281–1606)	1798 (664–3196)	873 (801–946)
Algeria	1.79 (1.07–2.73)	0.57 (0.34–0.83)	− 1.23 (− 1.46 to − 0.99)	1.72 (1.03–2.57)	1.63 (1.02–2.35)	− 0.08 (− 0.38 to 0.21)	4470 (2675–6805)	1511 (917–2227)	− 2959 (− 3028 to − 2890)
Bahrain	0.36 (0.22–0.57)	0.24 (0.15–0.39)	− 0.11 (− 1.12 to 0.89)	0.86 (0.52–1.38)	1.22 (0.77–1.89)	0.37 (− 2.19 to 2.92)	12 (8–20)	15 (10–25)	3 (− 2 to 8)
Egypt	1.22 (0.75–1.81)	0.44 (0.26–0.71)	− 0.78 (− 0.92 to − 0.65)	1.15 (0.71–1.71)	1.09 (0.69–1.75)	− 0.06 (− 0.21 to 0.09)	6056 (3729–8947)	3488 (2054–5672)	− 2568 (− 2657 to − 2479)
Iran (Islamic Republic of)	1.84 (1.22–2.21)	0.56 (0.46–0.66)	− 1.28 (− 1.37 to − 1.19)	1.51 (1.00–1.80)	1.64 (1.36–1.92)	0.13 (0.02–0.23)	10,653 (7024–12,778)	2732 (2247–3208)	− 7920 (− 7978 to − 7863)
Iraq	0.54 (0.31–0.95)	0.21 (0.13–0.37)	− 0.33 (− 0.53 to − 0.13)	0.60 (0.35–1.07)	0.56 (0.35–1.00)	− 0.04 (− 0.25 to 0.18)	928 (533–1643)	703 (430–1270)	− 225 (− 273 to − 178)
Jordan	0.38 (0.24–0.59)	0.17 (0.10–0.27)	− 0.21 (− 0.49 to 0.07)	0.79 (0.49–1.24)	0.70 (0.43–1.08)	− 0.09 (− 0.64 to 0.47)	144 (89–222)	163 (97–261)	20 (3–36)
Kuwait	0.33 (0.23–0.49)	0.21 (0.14–0.30)	− 0.12 (− 0.53 to 0.29)	0.47 (0.32–0.69)	1.06 (0.73–1.49)	0.59 (− 0.33 to 1.52)	41 (28–61)	42 (28–61)	1 (− 6 to 8)
Lebanon	0.60 (0.37–0.96)	0.29 (0.17–0.48)	− 0.31 (− 0.80 to 0.18)	0.94 (0.58–1.52)	1.48 (0.89–2.33)	0.53 (− 0.59 to 1.66)	159 (98–255)	91 (54–149)	− 68 (− 84 to − 53)
Libya	0.62 (0.39–0.92)	0.32 (0.20–0.49)	− 0.30 (− 0.65 to 0.05)	1.34 (0.83–1.97)	1.01 (0.64–1.54)	− 0.34 (− 1.06 to 0.39)	269 (171–399)	136 (86–209)	− 133 (− 150 to − 116)
Morocco	1.12 (0.64–1.80)	0.59 (0.36–0.92)	− 0.53 (− 0.76 to − 0.29)	1.77 (1.04–2.76)	2.08 (1.28–3.17)	0.31 (− 0.10 to 0.72)	2566 (1461–4131)	1451 (893–2264)	− 1115 (− 1177 to − 1052)
Oman	0.58 (0.27–0.99)	0.32 (0.15–0.56)	− 0.26 (− 1.00 to 0.48)	0.84 (0.39–1.43)	0.95 (0.44–1.66)	0.11 (− 1.06 to 1.29)	104 (48–177)	68 (32–119)	− 36 (− 52 to − 20)
Palestine	0.36 (0.21–0.57)	0.21 (0.13–0.33)	− 0.15 (− 0.55 to 0.24)	0.64 (0.38–0.97)	0.91 (0.56–1.36)	0.27 (− 0.46 to 1.01)	73 (42–114)	96 (59–148)	23 (11–35)
Qatar	0.61 (0.37–0.96)	0.29 (0.18–0.46)	− 0.32 (− 1.67 to 1.03)	1.08 (0.68–1.67)	1.25 (0.79–1.92)	0.17 (− 2.09 to 2.43)	16 (10–25)	27 (16–42)	11 (5–17)
Saudi Arabia	0.21 (0.12–0.35)	0.10 (0.06–0.16)	− 0.11 (− 0.24 to 0.02)	0.36 (0.22–0.58)	0.56 (0.35–0.89)	0.20 (− 0.10 to 0.50)	309 (180–514)	183 (113–289)	− 126 (− 148 to − 103)
Sudan	1.26 (0.61–2.20)	0.83 (0.48–1.40)	− 0.43 (− 0.74 to − 0.12)	1.12 (0.57–1.90)	1.78 (1.10–2.76)	0.67 (0.41–0.93)	2408 (1166–4216)	3182 (1852–5391)	774 (688–861)
Syrian Arab Republic	0.28 (0.16–0.49)	0.20 (0.11–0.33)	− 0.08 (− 0.27 to 0.10)	0.39 (0.23–0.66)	0.44 (0.26–0.72)	0.05 (− 0.19 to 0.29)	376 (208–655)	254 (147–419)	− 122 (− 150 to − 94)
Tunisia	0.93 (0.57–1.42)	0.38 (0.23–0.60)	− 0.54 (− 0.88 to − 0.20)	1.63 (1.00–2.51)	1.58 (1.01–2.40)	− 0.05 (− 0.77 to 0.67)	692 (428–1063)	255 (154 to 397)	− 437 (− 465 to − 409)
Turkey	1.21 (0.51–2.06)	0.60 (0.39–0.85)	− 0.61 (− 0.80 to − 0.43)	2.40 (1.00–4.10)	2.81 (1.93–3.88)	0.41 (0.04–0.78)	6505 (2729–11,087)	2616 (1727–3731)	− 3889 (− 3998 to − 3780)
United Arab Emirates	0.48 (0.28–0.79)	0.18 (0.11–0.33)	− 0.30 (− 0.95 to 0.36)	0.85 (0.49–1.39)	0.81 (0.50–1.48)	− 0.04 (− 1.21 to 1.14)	53 (31–88)	55 (33–99)	2 (− 10 to 13)
Yemen	1.21 (0.63–2.02)	0.82 (0.47–1.33)	− 0.40 (− 0.71 to − 0.08)	1.16 (0.62–1.80)	1.10 (0.68–1.65)	− 0.06 (− 0.29 to 0.17)	1781 (928–2961)	2549 (1464–4154)	768 (698–839)
South Asia	3.68 (2.78–4.59)	2.05 (1.60–2.52)	− 1.63 (− 1.66 to − 1.60)	2.84 (2.17–3.53)	3.54 (2.84–4.27)	0.70 (0.67–0.73)	357,970 (270,284–446,208)	282,175 (220,020–345,911)	− 75,795 (− 76,165 to − 75,425)
Bangladesh	4.50 (1.81–7.24)	2.13 (1.30–3.34)	− 2.37 (− 2.61 to − 2.13)	2.95 (1.21–4.73)	3.31 (2.04–5.06)	0.36 (0.19–0.53)	46,519 (18,725–74,754)	25,056 (15,286–39,235)	− 21,463 (− 21,758 to − 21,169)
Bhutan	0.58 (0.09–1.35)	0.55 (0.24–1.29)	− 0.03 (− 2.55 to 2.49)	0.60 (0.10–1.36)	1.53 (0.66–3.49)	0.93 (− 2.29 to 4.15)	34 (5–79)	28 (12–66)	− 6 (− 22 to 10)
India	3.95 (2.97–4.91)	2.11 (1.63–2.60)	− 1.84 (− 1.87 to − 1.80)	3.00 (2.29–3.71)	3.93 (3.14–4.81)	0.94 (0.90–0.97)	292,803 (220,235–363,614)	215,452 (166,371–264,841)	− 77,351 (− 77,684 to − 77,019)
Nepal	2.00 (0.96–4.13)	1.01 (0.53–2.16)	− 0.99 (− 1.52 to − 0.46)	1.78 (0.83–3.68)	2.55 (1.38–5.49)	0.77 (0.21–1.33)	3622 (1732–7473)	2470 (1301–5289)	− 1152 (− 1274 to − 1030)
Pakistan	1.38 (0.78–2.28)	1.86 (1.05–3.10)	0.48 (0.34–0.62)	1.42 (0.80–2.37)	2.38 (1.38–3.84)	0.96 (0.84–1.08)	14,992 (8439–24,767)	39,170 (22,196–65,282)	24,177 (23,927–24,428)
Southeast Asia, East Asia, and Oceania	2.66 (1.99–3.05)	0.68 (0.57–0.78)	− 1.98 (− 2.00 to − 1.97)	3.90 (2.95–4.36)	2.30 (1.95–2.61)	− 1.60 (− 1.63 to − 1.57)	329,349 (246,613–377,691)	68,522 (57,828–78,490)	− 260,826 (− 261,063 to − 260,590)
East Asia	3.38 (2.59–3.92)	0.81 (0.69–0.96)	− 2.57 (− 2.59 to − 2.54)	5.89 (4.54–6.57)	3.83 (3.25–4.41)	− 2.06 (− 2.12 to − 2.01)	275,498 (210,730–319,353)	45,620 (38,499–54,010)	− 229,878 (− 230,093 to − 229,663)
China	3.45 (2.64–4.02)	0.82 (0.69–0.96)	− 2.64 (− 2.66 to − 2.61)	5.98 (4.59–6.67)	3.84 (3.26–4.43)	− 2.13 (− 2.19 to − 2.08)	271,246 (206,980–315,499)	44,021 (36,965–51,980)	− 227,225 (− 227,440 to − 227,009)
Democratic People’s Republic of Korea	2.61 (1.47–4.24)	1.02 (0.61–1.53)	− 1.59 (− 2.04 to − 1.13)	4.37 (2.78–6.30)	3.60 (2.22–5.35)	− 0.78 (− 1.50 to − 0.05)	3531 (1997–5743)	1288 (775–1941)	− 2243 (− 2313 to − 2174)
Taiwan Province of China	0.47 (0.37–0.60)	0.40 (0.27–0.58)	− 0.06 (− 0.23 to 0.10)	1.30 (1.02–1.65)	3.05 (2.21–4.15)	1.75 (0.94–2.55)	721 (566–919)	311 (209–446)	− 410 (− 428 to − 392)
Oceania	1.47 (1.09–2.10)	1.02 (0.75–1.65)	− 0.44 (− 0.82 to − 0.07)	1.67 (1.24–2.40)	1.52 (1.16–2.43)	− 0.14 (− 0.53 to 0.24)	875 (650–1256)	1093 (798–1761)	218 (183–253)
American Samoa	1.26 (0.81–1.86)	0.99 (0.61–1.51)	− 0.27 (− 5.64 to 5.10)	3.69 (2.37–5.38)	3.88 (2.53–5.81)	0.19 (− 14.01 to 14.39)	5 (3–8)	5 (3–7)	− 1 (− 3 to 2)
Cook Islands	3.72 (2.46–5.19)	1.23 (0.78–1.86)	− 2.49 (− 14.84 to 9.86)	9.18 (6.24–12.60)	9.61 (6.23–14.19)	0.43 (− 50.34 to 51.20)	6 (4–8)	1 (1–2)	− 5 (− 7 to − 3)
Fiji	2.74 (1.75–4.11)	2.54 (1.65–3.84)	− 0.20 (− 2.27 to 1.87)	4.38 (2.87–6.36)	4.67 (3.13–6.70)	0.29 (− 2.34 to 2.91)	188 (120–282)	165 (108–250)	− 23 (− 38 to − 7)
Guam	3.88 (2.57–5.33)	3.18 (2.20–4.50)	− 0.70 (− 5.85 to 4.46)	10.34 (7.02–14.13)	12.34 (8.65–16.39)	1.99 (− 10.81 to 14.80)	36 (24–49)	35 (24–50)	− 1 (− 7 to 5)
Kiribati	6.18 (3.97–9.08)	4.40 (2.61–6.76)	− 1.78 (− 10.75 to 7.19)	5.43 (3.64–7.90)	6.43 (4.08–9.69)	1.00 (− 6.67 to 8.67)	37 (24–55)	44 (26–67)	6 (− 1 to 14)
Marshall Islands	3.08 (2.00–4.45)	3.21 (2.02–4.81)	0.13 (− 8.03 to 8.29)	5.98 (3.88–8.55)	7.06 (4.64–9.92)	1.08 (− 11.67 to 13.83)	16 (10–22)	15 (9–22)	− 1 (− 5 to 4)
Micronesia (Federated States of)	4.25 (2.63–6.40)	2.69 (0.60–4.12)	− 1.56 (− 9.40 to 6.29)	5.96 (3.79–8.86)	6.11 (1.41–9.03)	0.15 (− 11.98 to 12.27)	47 (29–71)	23 (5–35)	− 24 (− 32 to − 15)
Nauru	4.28 (2.80–6.33)	3.88 (2.43–5.86)	− 0.40 (− 22.35 to 21.55)	6.81 (4.56–9.99)	7.23 (4.59–10.49)	0.42 (− 28.82 to 29.66)	4 (2–6)	4 (2–5)	0 (− 2 to 2)
Niue	3.00 (1.92–4.49)	2.70 (1.63–4.14)	− 0.30 (− 49.35 to 48.76)	6.80 (4.50–10.01)	7.33 (4.73–11.12)	0.53 (− 95.37 to 96.43)	1 (0–1)	0 (0–0)	0 (− 1 to 1)
Northern Mariana Islands	1.80 (1.14–2.65)	2.14 (1.37–3.11)	0.34 (− 8.64 to 9.32)	6.29 (4.09–9.00)	9.00 (5.99–12.83)	2.71 (− 24.57 to 29.98)	5 (3–7)	5 (3–7)	0 (− 3 to 2)
Palau	2.49 (1.52–3.79)	1.68 (1.10–2.49)	− 0.81 (− 15.51 to 13.89)	4.49 (2.78–6.57)	4.62 (3.15–6.88)	0.13 (− 25.69 to 25.94)	3 (2–5)	2 (1–2)	− 1 (− 3 to 0)
Papua New Guinea	0.64 (0.33–1.51)	0.53 (0.28–1.30)	− 0.11 (− 0.89 to 0.67)	0.65 (0.35–1.50)	0.74 (0.40–1.77)	0.10 (− 0.46 to 0.65)	241 (123–569)	423 (221–1028)	182 (134–230)
Samoa	3.39 (2.07–5.07)	1.90 (1.16–2.92)	− 1.48 (− 5.63 to 2.67)	5.87 (3.76–8.57)	6.11 (3.99–8.97)	0.24 (− 6.85 to 7.33)	59 (36–88)	39 (23–59)	− 20 (− 29 to − 11)
Solomon Islands	3.52 (1.63–5.99)	3.23 (1.59–5.41)	− 0.29 (− 4.41 to 3.83)	3.64 (1.69–6.06)	4.84 (2.45–7.92)	1.20 (− 2.49 to 4.88)	123 (57–209)	191 (94–319)	68 (47–89)
Tokelau	2.51 (1.61–3.67)	1.37 (0.80–2.26)	− 1.14 (− 45.70 to 43.42)	5.77 (3.80–8.19)	5.96 (3.74–9.32)	0.18 (− 111.95 to 112.32)	0 (0–1)	0 (0–0)	0 (− 1 to 0)
Tonga	0.93 (0.59–1.38)	0.83 (0.52–1.25)	− 0.10 (− 3.29 to 3.09)	2.48 (1.58–3.62)	3.00 (1.99–4.40)	0.52 (− 7.29 to 8.33)	9 (6–14)	7 (5–11)	− 2 (− 5 to 2)
Tuvalu	5.56 (3.33–8.28)	2.31 (1.45–3.59)	− 3.25 (− 28.59 to 22.09)	6.37 (3.94–9.14)	6.43 (4.35–9.29)	0.05 (− 30.07 to 30.18)	4 (2–6)	2 (1–3)	− 2 (− 4 to 1)
Vanuatu	3.13 (1.84–4.93)	3.09 (1.89–4.63)	− 0.04 (− 4.78 to 4.70)	4.98 (3.02–7.58)	6.22 (3.86–9.13)	1.24 (− 4.95 to 7.43)	45 (26–70)	82 (50–123)	37 (27− 47)
Southeast Asia	1.27 (0.85–1.56)	0.50 (0.39–0.59)	− 0.77 (− 0.80 to − 0.74)	1.43 (0.97–1.75)	1.27 (1.01–1.47)	− 0.15 (− 0.19 to − 0.12)	52,976 (35,428–65,243)	21,809 (17,091–25,853)	− 31,166 (− 31,306 to − 31,027)
Cambodia	1.63 (0.84–2.65)	0.64 (0.33–1.04)	− 0.99 (− 1.44 to − 0.55)	1.20 (0.61–1.92)	1.40 (0.74–2.26)	0.20 (− 0.23 to 0.63)	1592 (821–2581)	771 (403–1263)	− 821 (− 874 to − 768)
Indonesia	0.83 (0.55–1.08)	0.40 (0.31–0.49)	− 0.43 (− 0.48 to − 0.39)	0.88 (0.58–1.15)	1.07 (0.84–1.29)	0.18 (0.13–0.24)	14,127 (9304–18,395)	7087 (5505–8758)	− 7039 (− 7123 to − 6956)
Lao People’s Democratic Republic	2.75 (1.65–4.11)	0.81 (0.47–1.25)	− 1.94 (− 2.65 to − 1.23)	1.63 (0.97–2.41)	1.59 (0.99–2.38)	− 0.04 (− 0.59 to 0.51)	1104 (663–1648)	441 (253–681)	− 663 (− 698 to − 628)
Malaysia	0.27 (0.16–0.54)	0.16 (0.08–0.35)	− 0.11 (− 0.32 to 0.10)	0.58 (0.33–1.15)	0.59 (0.33–1.29)	0.01 (− 0.38 to 0.41)	413 (237–821)	311 (164–685)	− 102 (− 142 to − 62)
Maldives	0.71 (0.38–1.17)	0.18 (0.11–0.30)	− 0.53 (− 2.42 to 1.37)	0.86 (0.46–1.40)	0.88 (0.55–1.43)	0.03 (− 3.20 to 3.26)	15 (8–25)	4 (3–7)	− 11 (− 16 to − 6)
Mauritius	2.75 (2.21–3.47)	1.10 (0.77–1.54)	− 1.65 (− 2.70 to − 0.60)	7.42 (5.89–9.34)	5.56 (4.09–7.40)	− 1.86 (− 5.21 to 1.50)	247 (198–312)	68 (48–95)	− 179 (− 188 to − 170)
Myanmar	1.86 (0.80–3.36)	0.68 (0.34–1.07)	− 1.18 (− 1.48 to − 0.89)	1.24 (0.53–2.15)	1.33 (0.70–2.00)	0.09 (− 0.13 to 0.30)	6696 (2866–12,079)	2590 (1310–4083)	− 4105 (− 4228 to − 3983)
Philippines	0.89 (0.66–1.05)	0.55 (0.39–0.67)	− 0.34 (− 0.40 to − 0.29)	1.00 (0.74–1.17)	1.10 (0.80–1.25)	0.10 (0.03− 0.17)	5233 (3885–6166)	4665 (3301–5672)	− 568 (− 614 to − 522)
Seychelles	0.28 (0.17–0.45)	0.26 (0.15–0.43)	− 0.03 (− 2.79 to 2.73)	0.85 (0.52–1.33)	1.00 (0.59–1.73)	0.14 (− 7.52 to 7.81)	2 (1–3)	1 (1–2)	0 (− 2 to 1)
Sri Lanka	4.96 (3.49–6.92)	1.25 (0.79–1.92)	− 3.71 (− 4.13 to − 3.29)	6.26 (4.45–8.71)	4.91 (3.23–7.12)	− 1.34 (− 2.09 to − 0.60)	7085 (4982–9881)	1730 (1087–2654)	− 5355 (− 5423 to − 5287)
Thailand	2.35 (1.30–3.49)	0.51 (0.33–0.77)	− 1.84 (− 2.04 to − 1.64)	3.23 (1.77–4.75)	1.48 (1.01–2.24)	− 1.76 (− 2.05 to − 1.46)	10,849 (5990–16,095)	1549 (1005–2309)	− 9299 (− 9400 to − 9199)
Timor-Leste	1.40 (0.71–2.31)	0.64 (0.36–1.00)	− 0.77 (− 2.35 to 0.82)	1.24 (0.67–1.94)	1.53 (0.89–2.35)	0.28 (− 1.16 to 1.72)	88 (45–145)	80 (45–126)	− 8 (− 22 to 6)
Viet Nam	0.89 (0.52–1.39)	0.46 (0.28–0.72)	− 0.43 (− 0.56 to − 0.31)	1.74 (1.07–2.62)	1.73 (1.08–2.65)	0.00 (− 0.26 to 0.26)	5455 (3185–8479)	2482 (1514–3875)	− 2973 (− 3058 to − 2889)
Sub-Saharan Africa	1.76 (1.27–2.15)	1.24 (0.94–1.59)	− 0.53 (− 0.56 to − 0.50)	1.32 (0.97–1.60)	1.65 (1.29–2.02)	0.32 (0.30–0.35)	83,337 (60,070–101,537)	130,065 (98,466–166,859)	46,728 (46,495–46,961)
Central Sub-Saharan Africa	2.11 (1.42–3.00)	1.41 (0.88–2.34)	− 0.70 (− 0.86 to − 0.54)	1.49 (1.01–2.08)	1.79 (1.18–2.85)	0.30 (0.19–0.41)	10,919 (7357–15,532)	18,252 (11,445–30,373)	7332 (7175–7490)
Angola	2.61 (1.38–4.37)	1.41 (0.81–2.29)	− 1.19 (− 1.74 to − 0.65)	1.45 (0.80–2.42)	1.95 (1.23–3.07)	0.50 (0.21–0.78)	2522 (1334–4233)	4323 (2489–7007)	1801 (1713–1889)
Central African Republic	2.99 (1.64–4.84)	2.38 (1.38–3.89)	− 0.61 (− 1.79 to 0.57)	1.64 (0.91–2.62)	1.57 (0.90–2.60)	− 0.07 (− 0.60 to 0.47)	739 (406–1195)	1201 (698–1963)	462 (416–508)
Congo	1.71 (0.78–3.03)	1.06 (0.59–1.79)	− 0.65 (− 1.71 to 0.41)	1.52 (0.69–2.65)	1.62 (0.93–2.71)	0.09 (− 0.72 to 0.91)	409 (186–723)	497 (276–837)	87 (51–123)
Democratic Republic of the Congo	1.94 (1.10–3.07)	1.39 (0.75–2.50)	− 0.55 (− 0.80 to − 0.31)	1.49 (0.89–2.31)	1.78 (0.98–3.16)	0.29 (0.12–0.46)	6987 (3944–11,073)	11,967 (6455–21,583)	4979 (4820–5138)
Equatorial Guinea	2.70 (1.49–4.58)	0.85 (0.38–1.74)	− 1.85 (− 4.64 to 0.93)	1.60 (0.91–2.63)	1.26 (0.60–2.51)	− 0.34 (− 1.85 to 1.17)	105 (58–178)	124 (56–254)	18 (− 2 to 39)
Gabon	1.76 (0.87–2.96)	0.99 (0.54–1.72)	− 0.77 (− 2.43 to 0.90)	1.78 (0.90–2.98)	1.92 (1.06–3.37)	0.14 (− 1.53 to 1.81)	157 (78–264)	141 (77–246)	− 15 (− 35 to 5)
Eastern Sub-Saharan Africa	2.41 (1.59–3.01)	1.40 (1.03–1.78)	− 1.01 (− 1.07 to − 0.95)	1.50 (1.01–1.87)	1.92 (1.44–2.37)	0.42 (0.37–0.46)	46,153 (30,530–57,647)	57,265 (42,028–73,055)	11,113 (10,935–11,290)
Burundi	3.18 (1.87–5.16)	1.99 (1.19–3.19)	− 1.19 (− 1.97 to − 0.41)	1.85 (1.10–2.91)	2.23 (1.36–3.47)	0.37 (− 0.05 to 0.79)	1647 (965–2666)	2333 (1395–3745)	687 (624–750)
Comoros	1.75 (0.30–3.23)	1.29 (0.63–2.27)	− 0.46 (− 3.67 to 2.75)	1.53 (0.27–2.77)	2.14 (1.14–3.55)	0.61 (− 1.89 to 3.10)	82 (14–151)	76 (37–133)	− 6 (− 25 to 12)
Djibouti	1.52 (0.74–2.61)	1.49 (0.73–2.66)	− 0.04 (− 2.39 to 2.32)	1.48 (0.83–2.46)	2.26 (1.23–3.83)	0.78 (− 1.11 to 2.68)	71 (35–122)	139 (68–249)	67 (49–86)
Eritrea	2.11 (1.11–3.64)	1.68 (0.87–2.91)	− 0.43 (− 1.45 to 0.59)	0.63 (0.32–1.09)	2.43 (1.31–4.03)	1.80 (1.22–2.38)	633 (331–1089)	1065 (549–1843)	433 (384–482)
Ethiopia	3.22 (1.89–4.52)	1.26 (0.87–1.76)	− 1.96 (− 2.15 to − 1.77)	1.36 (0.79–1.90)	2.25 (1.59–2.99)	0.89 (0.79–1.00)	16,751 (9861–23,548)	13,559 (9355–19,030)	− 3191 (− 3323 to − 3060)
Kenya	1.12 (0.74–1.48)	0.99 (0.67–1.42)	− 0.13 (− 0.28 to 0.03)	1.51 (1.03–1.96)	1.61 (1.13–2.28)	0.09 (− 0.08 to 0.27)	2728 (1813–3608)	4787 (3235–6875)	2060 (1998–2121)
Madagascar	3.75 (1.90–6.13)	1.40 (0.84–2.28)	− 2.35 (− 2.93 to − 1.76)	2.00 (1.01–3.25)	1.93 (1.19–3.03)	− 0.07 (− 0.37 to 0.24)	4365 (2210–7136)	3565 (2130–5803)	− 800 (− 895 to − 705)
Malawi	2.74 (1.54–4.44)	1.59 (0.89–2.64)	− 1.15 (− 1.71 to − 0.58)	1.91 (1.10–3.04)	1.79 (1.03–2.93)	− 0.12 (− 0.47 to 0.22)	2494 (1399–4038)	3184 (1778–5267)	690 (611–770)
Mozambique	2.17 (1.17–3.52)	1.57 (0.83–2.74)	− 0.61 (− 1.05 to − 0.16)	1.46 (0.83–2.34)	1.34 (0.73–2.31)	− 0.12 (− 0.36 to 0.12)	2915 (1565–4728)	4715 (2495–8247)	1799 (1699–1900)
Rwanda	3.19 (1.85–5.24)	1.38 (0.80–2.29)	− 1.81 (− 2.50 to − 1.12)	1.71 (0.99–2.77)	2.55 (1.60–4.02)	0.84 (0.36–1.31)	2209 (1282–3624)	1660 (957–2748)	− 548 (− 614 to − 483)
Somalia	1.70 (0.88–2.95)	2.20 (1.11–3.74)	0.50 (− 0.09 to 1.09)	0.86 (0.45–1.46)	1.85 (0.97–3.13)	0.99 (0.71− 1.27)	1342 (693–2323)	4493 (2266–7614)	3151 (3061–3240)
South Sudan	1.16 (0.65–1.98)	1.28 (0.71–2.38)	0.12 (− 0.47 to 0.72)	0.99 (0.57–1.53)	1.66 (0.99–2.74)	0.68 (0.29− 1.07)	673 (378–1150)	1285 (712–2386)	612 (558–666)
Uganda	1.32 (0.54–2.44)	1.31 (0.71–2.18)	− 0.01 (− 0.39 to 0.37)	1.21 (0.51–2.23)	1.76 (0.98–2.86)	0.55 (0.27–0.83)	2222 (916–4117)	5669 (3070–9461)	3447 (3340–3553)
United Republic of Tanzania	2.35 (1.35–3.87)	1.47 (0.87–2.32)	− 0.88 (− 1.19 to − 0.57)	2.27 (1.33–3.57)	2.23 (1.40–3.47)	− 0.04 (− 0.28 to 0.21)	6081 (3488–10,017)	7998 (4720–12,631)	1916 (1797–2035)
Zambia	2.32 (1.34–3.82)	1.51 (0.89–2.51)	− 0.81 (− 1.36 to − 0.25)	1.70 (1.01–2.80)	1.81 (1.10–3.03)	0.11 (− 0.25 to 0.48)	1906 (1105–3144)	2691 (1576–4468)	785 (713–856)
Southern Sub-Saharan Africa	0.33 (0.25–0.43)	0.23 (0.15–0.33)	− 0.10 (− 0.15 to − 0.04)	0.48 (0.37–0.62)	0.29 (0.19–0.42)	− 0.19 (− 0.25 to − 0.12)	1603 (1236–2094)	1327 (871–1918)	− 277 (− 312 to − 242)
Botswana	0.42 (0.24–0.68)	0.44 (0.26–0.71)	0.02 (− 0.64 to 0.69)	0.56 (0.33–0.92)	0.70 (0.43–1.13)	0.14 (− 0.60 to 0.89)	58 (34–94)	77 (45–124)	20 (8–32)
Eswatini	0.50 (0.29–0.80)	0.51 (0.30–0.83)	0.01 (− 0.91 to 0.93)	0.54 (0.32–0.88)	0.49 (0.29–0.79)	− 0.05 (− 0.80 to 0.70)	43 (25–69)	51 (31–83)	8 (− 1 to 18)
Lesotho	0.58 (0.32–0.95)	0.63 (0.36–1.03)	0.05 (− 0.71 to 0.81)	0.58 (0.34–0.93)	0.50 (0.29–0.82)	− 0.07 (− 0.59 to 0.44)	106 (59–174)	108 (62–176)	2 (− 13 to 17)
Namibia	0.35 (0.13–0.58)	0.30 (0.16–0.51)	− 0.04 (− 0.73 to 0.64)	0.41 (0.15–0.71)	0.42 (0.22–0.69)	0.01 (− 0.66 to 0.67)	48 (18–79)	60 (31–102)	13 (0–25)
South Africa	0.26 (0.20–0.36)	0.05 (0.02–0.10)	− 0.21 (− 0.27 to − 0.14)	0.40 (0.32–0.56)	0.07 (0.04–0.15)	− 0.33 (− 0.41 to − 0.25)	828 (655–1174)	178 (86–363)	− 650 (− 676 to − 623)
Zimbabwe	0.47 (0.25–0.80)	0.60 (0.32–0.97)	0.13 (− 0.15 to 0.41)	0.64 (0.34–1.09)	0.54 (0.30–0.88)	− 0.10 (− 0.36 to 0.15)	521 (269–878)	851 (450–1371)	330 (289–370)
Western Sub-Saharan Africa	1.37 (0.97–1.80)	1.17 (0.87–1.53)	− 0.20 (− 0.25 to − 0.14)	1.15 (0.83–1.48)	1.55 (1.20–1.94)	0.40 (0.36–0.44)	24,662 (17,526–32,490)	53,221 (39,266–69,427)	28,560 (28,401–28,718)
Benin	1.73 (0.99–2.77)	1.49 (0.80–2.57)	− 0.24 (− 0.89 to 0.40)	1.49 (0.89–2.31)	1.88 (1.16–2.97)	0.39 (− 0.05 to 0.83)	804 (461–1285)	1829 (983–3151)	1025 (968–1082)
Burkina Faso	1.79 (0.94–3.01)	1.78 (0.89–3.15)	0.00 (− 0.53 to 0.52)	1.40 (0.75–2.23)	1.91 (1.05–3.11)	0.50 (0.19–0.82)	1752 (919–2951)	3961 (1979–6992)	2209 (2118–2301)
Cabo Verde	1.10 (0.60–1.80)	0.88 (0.50–1.47)	− 0.22 (− 2.41 to 1.97)	2.25 (1.22–3.69)	2.79 (1.59–4.53)	0.54 (− 3.71 to 4.80)	37 (20–61)	35 (20–59)	− 2 (− 11 to 7)
Cameroon	1.43 (0.79–2.35)	1.55 (0.86–2.60)	0.12 (− 0.30 to 0.54)	1.31 (0.74–2.07)	1.81 (1.07–2.86)	0.50 (0.21− 0.80)	1409 (777–2307)	4452 (2461–7468)	3043 (2959–3126)
Chad	1.56 (0.86–2.56)	1.96 (1.07–3.32)	0.40 (− 0.19 to 0.99)	1.18 (0.67–1.87)	1.90 (1.15–3.04)	0.72 (0.38–1.07)	889 (487–1454)	3336 (1824–5654)	2447 (2374–2519)
Côte d’Ivoire	2.14 (1.17–3.51)	1.75 (0.92–3.00)	− 0.38 (− 0.88 to 0.11)	1.92 (1.09–3.05)	2.04 (1.18–3.41)	0.13 (− 0.22 to 0.48)	2430 (1330–3995)	4138 (2174–7094)	1708 (1616–1800)
Gambia	1.16 (0.59–2.02)	0.99 (0.50–1.69)	− 0.18 (− 1.53 to 1.18)	1.19 (0.64–1.97)	1.81 (0.94− 3.04)	0.62 (− 0.62 to 1.87)	110 (56–190)	218 (111–373)	108 (87–129)
Ghana	1.19 (0.68–1.93)	1.14 (0.63–1.90)	− 0.05 (− 0.37 to 0.28)	1.13 (0.69–1.73)	1.67 (1.00–2.68)	0.54 (0.28–0.80)	1687 (963–2734)	3117 (1724–5177)	1430 (1356–1504)
Guinea	1.72 (0.94–2.90)	1.84 (0.98–3.03)	0.12 (− 0.54 to 0.78)	1.24 (0.67–2.08)	1.76 (0.99–2.77)	0.52 (0.13–0.92)	937 (513–1577)	2307 (1229–3795)	1370 (1307–1432)
Guinea-Bissau	2.54 (1.38–4.39)	1.69 (0.94–2.82)	− 0.85 (− 2.72 to 1.01)	1.37 (0.75–2.28)	1.74 (0.99–2.92)	0.37 (− 0.60 to 1.33)	252 (137–436)	307 (171–513)	54 (28–81)
Liberia	1.70 (0.94–2.72)	1.21 (0.67–1.98)	− 0.49 (− 1.44 to 0.46)	0.74 (0.42–1.15)	1.60 (0.96–2.47)	0.86 (0.35–1.36)	339 (188–542)	563 (311–919)	224 (193–255)
Mali	1.76 (0.99–2.88)	1.48 (0.77–2.56)	− 0.28 (− 0.78 to 0.23)	1.14 (0.64–1.84)	1.62 (0.92–2.61)	0.48 (0.19–0.77)	1418 (799–2319)	3261 (1684–5617)	1844 (1766–1922)
Mauritania	1.28 (0.71–2.13)	0.80 (0.41–1.51)	− 0.48 (− 1.42 to 0.46)	1.14 (0.63–1.86)	1.53 (0.85–2.66)	0.39 (− 0.43 to 1.21)	244 (136–406)	322 (166–607)	78 (49–108)
Niger	1.82 (0.89–3.11)	1.44 (0.74–2.51)	− 0.38 (− 0.95 to 0.18)	1.22 (0.60–2.01)	1.65 (0.93–2.62)	0.43 (0.12–0.73)	1425 (696–2430)	3519 (1812–6134)	2094 (2009–2178)
Nigeria	1.01 (0.63–1.51)	0.79 (0.53–1.20)	− 0.22 (− 0.32 to − 0.13)	0.90 (0.59–1.32)	1.20 (0.82–1.76)	0.29 (0.22–0.37)	8392 (5182–12,503)	17,418 (11,679–26,461)	9026 (8891–9161)
Sao Tome and Principe	0.54 (0.32–0.90)	0.24 (0.14–0.41)	− 0.30 (− 2.47 to 1.87)	0.49 (0.30–0.79)	0.64 (0.38–1.08)	0.15 (− 2.29 to 2.59)	7 (4–12)	5 (3–8)	− 2 (− 6 to 1)
Senegal	1.92 (1.11–3.05)	1.49 (0.83–2.46)	− 0.43 (− 0.96 to 0.10)	1.79 (1.04–2.79%)	2.49 (1.50–3.99)	0.70 (0.23–1.18)	1436 (827–2279)	2178 (1211–3587)	742 (679–804)
Sierra Leone	1.60 (0.80–2.71)	1.62 (0.83–2.76)	0.03 (− 0.84 to 0.89)	1.23 (0.64–2.08)	1.66 (0.93–2.65)	0.42 (− 0.10 to 0.95)	507 (254–861)	1246 (639–2119)	740 (691–788)
Togo	1.59 (0.97–2.55)	1.38 (0.76–2.25)	− 0.21 (− 0.90 to 0.48)	1.44 (0.89–2.22)	1.70 (1.04–2.65)	0.27 (− 0.24 to 0.77)	585 (358–941)	1009 (555–1648)	423 (382–464)
Africa	1.66 (1.21–2.00)	1.14 (0.87–1.44)	− 0.52 (− 0.55 to − 0.50)	1.33 (0.98–1.58)	1.63 (1.29–1.97)	0.30 (0.28–0.32)	100,011 (72,821–120,318)	140,109 (107,467–177,307)	40,098 (39,863–40,333)
America	1.11 (1.06–1.16)	1.37 (1.25–1.50)	0.26 (0.25–0.27)	2.29 (2.19–2.40)	5.10 (4.77–5.50)	2.81 (2.79–2.83)	59,046 (56,400–61,756)	80,085 (73,161–87,704)	21,039 (20,984–21,094)
Asia	2.90 (2.29–3.33)	1.41 (1.16–1.67)	− 1.49 (− 1.50 to − 1.47)	3.16 (2.51–3.60)	3.16 (2.63–3.68)	0.00 (− 0.02 to 0.02)	727,092 (575,821–834,769)	384,336 (315,341–455,401)	− 342,756 (− 343,127 to − 342,385)
Europe	1.52 (1.42–1.63)	0.87 (0.79–0.97)	− 0.65 (− 0.67 to − 0.64)	4.59 (4.29–4.92)	5.72 (5.30–6.38)	1.14 (1.08–1.19)	68,923 (64,478–74,040)	31,331 (28,589–35,091)	− 37,593 (− 37,643 to − 37,542)

Data are presented as country/territory-wise comparison of death rates (per 100,000 individuals), death percentage in all-cause mortality, and years of life lost (YLLs), together with 95% uncertainty intervals (UIs)-1990 and 2019

The portion of suicide-related deaths in all-cause mortality differed substantially. The highest fraction was observed in Greenland (33.02%; 95% UI = 24.36%–41.53%), Canada (14.69%; 95% UI = 11.59%–18.14%), Japan (13.31%; 95% UI = 12.43%–14.29%), Kazakhstan (12.44%; 95% UI = 9.59%–15.82%), and Guam (12.34%; 95% UI = 8.65%–16.39%). The lowest values were found in South Africa (0.07%; 95% UI = 0.04%–0.15%), Bahamas (0.34%; 95% UI = 0.23%–0.49%), Namibia (0.42%; 95% UI = 0.22%–0.69%), Syria (0.44%; 95% UI = 0.26%–0.72%), and Eswatini (0.49%; 95% UI = 0.29%–0.79%).

Worldwide, there were approximately 636,196 YLLs due to suicide [95% UI = 540,383–740,009; males 381,075 (95% UI = 299,773–465,128), females 255,121 (95% UI = 217,770–295,046); Table 1, Supplementary Tables 3 and 4]. The rate of YLLs per 100,000 globally was 99.07 (95% UI = 84.15–115.23), and it ranged from 3.69 (95% UI = 1.79–7.52) in South Africa to 572.07 (95% UI = 391.57–806.93) in Greenland. The contribution of suicide-related YLLs to all-cause YLLs in this age group ranged from 0.07% (95% UI = 0.04%–0.15%) in South Africa to 33.02% (95% UI = 24.36%–41.53%) in Greenland.

**Fig. 1:**
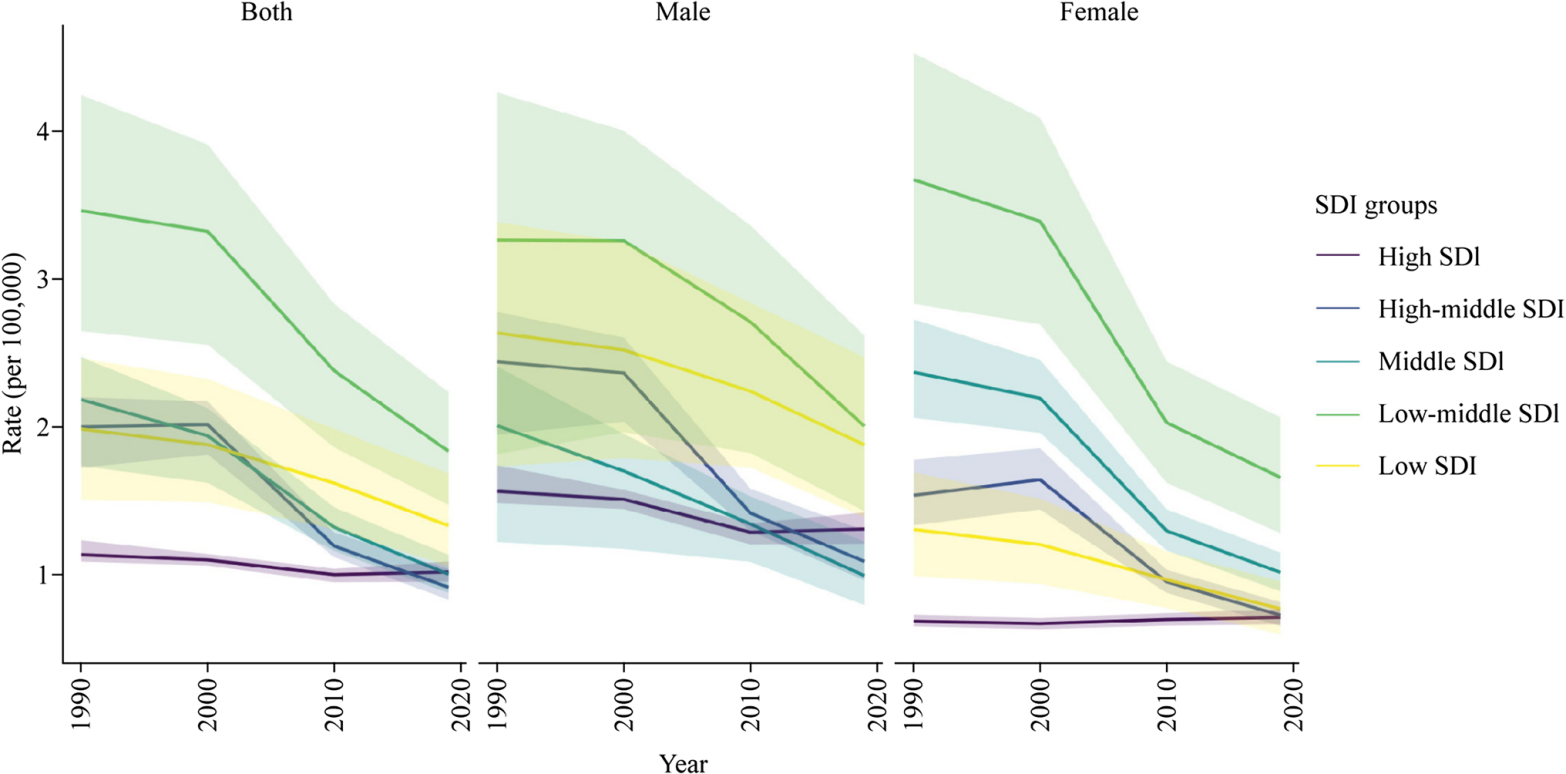
Mortality rate per 100,000 due to intentional suicide among children aged 10–14 years in 1990–2019 by sociodemographic index (SDI) group

There was a positive association between the SDI and the contribution of suicide-related deaths to all-cause deaths (Fig. 1; Table 2). In high-SDI countries, the contribution was 8.95% (95% UI = 8.38%–9.52%), high-middle SDI 4.36% (95% UI = 4.02%–4.90%), middle SDI 2.80% (95% UI = 2.46%–3.16%), low-middle SDI 3.13% (95% UI = 2.52%–3.74%), and low SDI 1.81% (95% UI = 1.46%–2.20%).

**Table 2:** Deaths due to suicide among children aged 10–14 years

Location	Death rate 1990 (95% UI)	Death rate 2019 (95% UI)	Change (95% UI)	Death percentage 1990 (95% UI), %	Death percentage 2019 (95% UI), %	Change (95% UI), %	YLLs 1990 (95% UI)	YLLs 2019 (95% UI)	Change (95% UI)
Low SDI	1.98 (1.51–2.46)	1.33 (1.06–1.68)	− 0.65 (− 0.68 to − 0.62)	1.38 (1.05–1.70)	1.81 (1.46–2.20)	0.43 (0.40–0.45)	99,766 (75,729–123,847)	146,517 (116,338–185,216)	46,750 (46,519–46,981)
Low-middle SDI	3.46 (2.65–4.24)	1.83 (1.47–2.24)	− 1.63 (− 1.65 to − 1.60)	2.85 (2.18–3.51)	3.13 (2.52–3.74)	0.29 (0.26–0.31)	351,171 (268,541–430,487)	247,851 (198,727–301,912)	− 103,320 (− 103,656 to − 102,984)
Middle SDI	2.18 (1.74–2.47)	1.00 (0.88–1.14)	− 1.18 (− 1.19 to − 1.17)	2.90 (2.31–3.24)	2.80 (2.46–3.16)	− 0.10 (− 0.12 to − 0.08)	304,765 (242,292–344,803)	141,352 (123,558–159,885)	− 163,413 (− 163,618 to − 163,208)
High-middle SDI	2.00 (1.72–2.20)	0.92 (0.83–1.04)	− 1.08 (− 1.10 to − 1.07)	4.36 (3.78–4.79)	4.36 (4.02–4.90)	− 0.01 (− 0.04 to 0.03)	149,983 (129,284–165,107)	56,476 (51,250–64,058)	− 93,507 (− 93,612 to − 93,402)
High SDI	1.14 (1.09–1.23)	1.02 (0.95–1.09)	− 0.12 (− 0.12 to − 0.11)	4.60 (4.39–5.03)	8.95 (8.38–9.53)	4.36 (4.31–4.41)	49,587 (47,538–53,867)	43,748 (40,948–46,813)	− 5839 (− 5878 to − 5799)

Data are presented as socio-demographic index (SDI)-wise comparison of death rates (per 100,000 individuals), death percentage in all-cause mortality, and years of life lost (YLLs), together with 95% uncertainty intervals (UIs)-1990 and 2019

Between 1990 and 2019, worldwide, the suicide-related mortality rate per 100,000 decreased from 2.33 (95% UI = 1.90–2.62) to 1.30 (95% UI = 1.10–1.51) (Table 1). The slightest improvement was observed in countries with high SDI, from 1.14 (95% UI = 1.09–1.23) in 1990 to 1.02 (95% UI = 0.95–1.09) in 2019. During the same time, globally, the percentage of deaths caused by suicide in the 10–14 years age group increased from 2.76% (95% UI = 2.25%–3.09%) to 2.78% (95% UI = 2.38%–3.15%). The most substantial change was observed in countries with a high SDI, where the fraction increased from 4.60% (95% UI = 4.39%–5.02%) to 8.95% (95% UI = 8.38%–9.53%).

## Discussion

### Significance of this study

This study represents a pioneering effort in addressing a pressing gap in the literature by investigating the magnitude of suicide-related deaths among children aged 10–14 years, shedding light on an issue that has not been extensively explored within this context. The importance of this study lies not only in its novel approach but also in its rigorous methodology, utilizing comprehensive data from multiple sources, including vital registration systems and population-based surveys. By employing a robust analytical framework, this study provides valuable insights into the global burden of suicide-related deaths and its contribution to all-cause mortality among early adolescents. The findings of this study hold meaningful implications for policymakers, healthcare providers, and researchers, urging them to recognize the distinct needs and vulnerabilities of this age group and develop targeted strategies to mitigate the impact of suicide and promote mental well-being.

### Global burden of intentional suicide-related deaths

The study’s results further elucidate the variations in mortality rates across different regions. Notably, countries such as Greenland, Mongolia, Kiribati, Uzbekistan, and Ecuador reported the highest rates of suicide-related deaths. This indicates that certain geographical contexts may be more prone to this issue, suggesting the presence of specific risk factors or sociocultural influences that contribute to suicide among children in these regions. Understanding these factors is crucial for tailoring interventions and prevention strategies to address the unique challenges faced by each region.

Additionally, the study revealed significant disparities in the proportion of suicide-related deaths in relation to all-cause mortality. Greenland, Canada, Japan, Kazakhstan, and Guam exhibited particularly high percentages, indicating the disproportionate impact of suicide on the overall mortality rates in these regions. This highlights the need for targeted interventions and comprehensive support systems to address the complex underlying factors driving suicide among children aged 10–14 years. When seeking effective suicide prevention measures, it becomes evident that there is no one-size-fits-all strategy that outshines the rest. Instead, it is imperative to evaluate combinations of evidence-based approaches, both at the individual and population levels, using rigorous research methodologies [19]. Efforts must focus not only on preventing suicide but also on improving mental health awareness, enhancing access to mental health services, and fostering a supportive environment that reduces stigma and promotes early detection and intervention.

### Socioeconomic factors and mental health

The association between the SDI and the contribution of suicide-related deaths to all-cause deaths provides important insights into the interplay between socioeconomic factors and mental health outcomes. The presented findings reveal a positive relationship between SDI and the proportion of suicide-related deaths among early adolescents. Specifically, countries with higher SDI exhibited a greater contribution of suicide-related deaths to all-cause deaths compared to countries with lower SDI.

These results emphasize the critical role of socioeconomic factors in shaping mental health outcomes. The observed association can be attributed to factors such as improved access to healthcare and mental health services, which lead to better detection and reporting of suicide cases. Additionally, higher levels of societal pressure, educational competition, and psychological distress in these countries may increase the risk of suicide among children. Furthermore, the increased access to the internet and the pervasive influence of social media platforms in high-SDI countries may also contribute to the prevalence of cyberbullying [20–22], potentially exacerbating the risk of suicide among children aged 10–14 years.

### Trends and changes over time

The findings reveal a promising decrease in the suicide-related mortality rate globally from 1990 to 2019. However, it is concerning that the slightest improvement was observed in countries with high SDI, indicating the need for further investigation into the factors hindering progress in these regions. Moreover, the increase in the percentage of deaths caused by suicide in the 10–14 years age group, particularly in high-SDI countries, necessitates a critical examination of the effectiveness of current interventions and the implementation of comprehensive strategies to address this worrisome trend.

### Implications for psychiatry and policy

It is essential for policymakers to acknowledge the gravity of the problem and prioritize the development and implementation of comprehensive policies and interventions that address children’s mental health needs and create safe and supportive environments. By exploring the implications of the presented study, both psychiatrists and policymakers can collaborate to mitigate the global burden of intentional suicide among children aged 10–14 years. This collaboration should focus on identifying and addressing the psychological and social determinants that contribute to suicide, such as underlying mental health conditions, adverse childhood experiences, social isolation, and limited access to mental healthcare services. Furthermore, efforts should be directed toward raising awareness, promoting mental health literacy, fostering early detection and intervention, and strengthening support systems within families, schools, and communities.

Importantly, it is crucial to recognize that psychiatric interventions play a pivotal role in preventing suicide-related deaths in adolescents. Extensive scientific literature supports the significance of psychiatric research in this context [23]. Reviews of descriptive epidemiological data reveal that psychiatric disorders are prevalent in a substantial percentage of adolescent suicide victims, with affective disorders such as major depressive disorder and bipolar disorder being common in these cases. Severe mental illness (SMI) stands out as a prominent and preventable predictor of suicide in adolescents. Notably, the risk of suicide associated with SMI is not significantly altered by adjustments in psychosocial or psychological factors alone.

Recent research has indicated the potential impact of advanced psychiatric treatments, including clozapine, electroconvulsive therapy, and lithium, in reducing excess suicide mortality rates among male adolescents [24]. These findings are consistent with existing evidence of the protective effects of such treatments in adults and prior studies demonstrating their efficacy in reducing suicidal behavior and nonfatal suicide attempts in adolescents [25–27].

The inclusion of medical treatments in discussions surrounding comprehensive strategies for self-harm prevention is of paramount importance, especially for an already vulnerable and underserved population. Therefore, collaboration between the fields of psychiatry and policy should aim to integrate psychiatric research and interventions into holistic approaches that address the multifaceted nature of self-harm and suicide in children aged 10–14 years. Collaboration can involve jointly developing and implementing evidence-based programs, establishing accessible mental health services in schools, and regularly evaluating the effectiveness of these interventions. Additionally, policymakers can allocate resources and funding to support mental health initiatives, research, and community programs aimed at suicide prevention in this age group.

### Limitations of the study

The study has several limitations specific to the topic of suicide among children aged 10–14 years. These include the availability of primary data and the challenge of representing uncertainty in locations with sparse or absent data. Additionally, the study’s reliance on predictive models introduces inherent limitations. Furthermore, the study’s findings should be interpreted with caution, as there may be cultural factors in certain countries that contribute to the underreporting of suicide among children [28]. One of these factors is cultural stigma linked with suicide and help-seeking, which varies by region [29–32]. Additionally, geographical differences in reporting standards across countries and territories may impact the accuracy and completeness of suicide data. A somewhat connected limitation pertains to the reliance of this study on ICD-10 codes for confirmed suicide deaths. It is essential to acknowledge that some suicide deaths among early adolescents might be categorized as “undetermined intent” (ICD-10 Y10-Y34), as discussed in a pharmacoepidemiological study of suicide-related deaths in adolescents [33], and the share of such categorization might differ by country. Considering this, it is plausible that accounting for cases classified under “undetermined intent” could contribute to a more comprehensive understanding of the burden of self-harm and suicide in this age group. This highlights the need for cultural sensitivity and further investigation to account for potential discrepancies in reporting practices and to obtain a more comprehensive understanding of the global burden of suicide in this age group.

In conclusion, the findings of this study reveal a concerning global burden of suicide-related deaths among children aged 10–14 years. Despite progress in reducing mortality rates, many children continue to die due to suicide, with males showing higher mortality rates than females. While there was an overall decline in suicide-related mortality rates between 1990 and 2019, the increase in the percentage of deaths caused by suicide within the 10–14 years age group demands urgent attention. The substantial change observed in countries with a high SDI underscores the need for tailored interventions to address this concern and prevent further increases. Geographical variations in suicide mortality rates emphasize the need for targeted interventions in regions with higher rates. The proportion of suicide-related deaths in all-cause mortality highlights the magnitude of this issue, particularly in Greenland, Canada, Japan, Kazakhstan, and Guam. Substantial YLLs due to suicide underscore the long-term impact of these tragic deaths. Efforts should focus on providing adequate mental health support, early intervention, and preventive measures to reduce the global burden of suicide-related YLLs.

### Supplementary Information

Below is the link to the electronic supplementary material.

Supplementary file 1 (PDF 509 KB)

## Data Availability

Data analyzed in this study were a re-analysis of existing data, which are openly available at locations cited in the Methods section.
