# Supplementary material for: Unveiling the silent crisis: global burden of suicide-related deaths among children aged 10–14 years
Source: World J Pediatr. 2024 Jan 19;20(4):371–91. doi: 10.1007/s12519-023-00781-z (PMC11052841; doi:10.1007/s12519-023-00781-z)
Supplement: Supplementary file 1 — (PDF 509 KB) [file 12519_2023_781_MOESM1_ESM.pdf]

Supplementary Table 1. Deaths due to suicide among boys aged 10-14 years: country/territory-wise comparison of death rates (per 100,000 individuals) together with 95% uncertainty intervals (UIs)– 1990-2019

Location	Death rate (95% UI) 1990	2000	2010	2019
Global	2.43 (1.66 to 2.84)	2.30 (1.67 to 2.64)	1.90 (1.46 to 2.20)	1.51 (1.18 to 1.84)
Central Europe, Eastern Europe, and Central Asia	3.73 (3.55 to 3.92)	4.57 (4.37 to 4.78)	3.31 (3.07 to 3.55)	2.71 (2.35 to 3.22)
Central Asia	3.84 (3.36 to 4.33)	4.69 (4.11 to 5.31)	4.47 (3.80 to 5.19)	3.97 (3.13 to 5.11)
Armenia	0.42 (0.27 to 0.60)	0.47 (0.31 to 0.71)	1.27 (0.85 to 1.85)	0.82 (0.49 to 1.32)
Azerbaijan	0.58 (0.33 to 1.39)	0.72 (0.43 to 1.46)	0.96 (0.54 to 1.56)	0.70 (0.34 to 1.27)
Georgia	0.77 (0.53 to 1.10)	0.72 (0.50 to 1.03)	0.74 (0.47 to 1.05)	0.66 (0.43 to 0.98)
Kazakhstan	7.39 (5.96 to 9.06)	9.41 (7.66 to 11.39)	7.94 (6.52 to 9.44)	5.24 (3.66 to 7.08)
Kyrgyzstan	6.33 (4.85 to 7.93)	5.61 (4.31 to 6.94)	3.92 (3.09 to 4.90)	4.57 (3.25 to 6.13)
Mongolia	8.27 (2.78 to 14.07)	8.19 (2.82 to 13.33)	10.98 (3.53 to 17.39)	6.46 (2.20 to 10.84)
Tajikistan	1.41 (0.93 to 2.12)	1.32 (0.82 to 2.12)	1.21 (0.71 to 1.93)	1.19 (0.66 to 1.99)
Turkmenistan	2.35 (1.67 to 3.24)	3.83 (2.86 to 4.98)	2.14 (1.52 to 2.90)	1.63 (0.97 to 2.49)
Uzbekistan	3.38 (2.66 to 4.21)	4.97 (3.92 to 6.19)	5.44 (4.20 to 6.92)	5.48 (3.61 to 7.68)
Central Europe	2.53 (2.30 to 2.79)	2.32 (2.13 to 2.54)	1.55 (1.41 to 1.72)	1.20 (1.00 to 1.45)
Albania	1.01 (0.56 to 1.77)	1.45 (0.80 to 2.16)	1.67 (0.65 to 2.92)	1.53 (0.60 to 2.76)
Bosnia and Herzegovina	2.94 (1.25 to 4.30)	1.81 (0.82 to 2.99)	1.08 (0.55 to 1.77)	1.53 (0.76 to 2.64)
Bulgaria	4.67 (3.65 to 5.80)	3.07 (2.36 to 3.94)	1.97 (1.37 to 2.72)	1.44 (0.89 to 2.26)
Croatia	2.77 (2.00 to 3.65)	2.45 (1.75 to 3.32)	1.41 (0.99 to 1.94)	1.11 (0.67 to 1.71)
Czechia	2.69 (2.02 to 3.45)	1.90 (1.44 to 2.46)	1.37 (0.97 to 1.85)	0.79 (0.49 to 1.20)
Hungary	2.63 (2.04 to 3.37)	2.01 (1.45 to 2.64)	1.11 (0.80 to 1.50)	0.90 (0.56 to 1.38)
Montenegro	2.63 (1.49 to 4.27)	3.51 (2.05 to 5.34)	1.68 (0.99 to 2.65)	1.78 (0.89 to 3.08)
North Macedonia	1.46 (0.80 to 2.50)	1.34 (0.78 to 2.24)	0.79 (0.45 to 1.31)	1.02 (0.53 to 1.74)
Poland	2.72 (2.46 to 2.99)	2.46 (2.25 to 2.68)	1.78 (1.60 to 1.96)	1.30 (1.04 to 1.61)
Romania	1.76 (1.34 to 2.27)	2.76 (2.15 to 3.44)	2.00 (1.52 to 2.54)	1.55 (1.02 to 2.20)
Serbia	2.79 (1.34 to 4.91)	1.76 (1.04 to 3.06)	0.71 (0.40 to 1.52)	0.61 (0.29 to 1.19)
Slovakia	1.72 (0.98 to 2.84)	1.65 (1.04 to 2.59)	1.16 (0.70 to 1.86)	0.88 (0.42 to 1.52)
Slovenia	2.99 (2.10 to 3.99)	2.76 (2.02 to 3.66)	1.36 (0.94 to 1.84)	1.90 (1.18 to 2.82)
Eastern Europe	4.44 (4.20 to 4.68)	5.64 (5.39 to 5.90)	3.54 (3.36 to 3.73)	2.62 (2.24 to 3.10)
Belarus	3.40 (2.58 to 4.38)	4.42 (3.43 to 5.60)	3.04 (2.22 to 4.05)	1.71 (0.97 to 2.71)
Estonia	6.44 (4.65 to 8.59)	3.84 (2.69 to 5.31)	2.04 (1.44 to 2.86)	1.52 (0.95 to 2.32)
Latvia	4.09 (2.96 to 5.42)	2.64 (1.86 to 3.55)	2.22 (1.51 to 3.08)	1.19 (0.69 to 1.92)
Lithuania	3.44 (2.52 to 4.53)	3.84 (2.89 to 5.00)	2.59 (1.84 to 3.53)	2.44 (1.61 to 3.55)
Republic of Moldova	2.73 (1.92 to 3.66)	3.03 (2.15 to 4.14)	2.41 (1.69 to 3.36)	1.85 (1.16 to 2.76)
Russian Federation	4.96 (4.73 to 5.22)	6.36 (6.13 to 6.62)	4.05 (3.86 to 4.26)	2.48 (2.11 to 3.00)
Ukraine	3.30 (2.68 to 4.09)	4.16 (3.37 to 5.03)	2.35 (1.87 to 2.87)	3.48 (2.35 to 5.02)
High-income	1.49 (1.42 to 1.64)	1.48 (1.42 to 1.54)	1.33 (1.25 to 1.40)	1.32 (1.22 to 1.43)
Australasia	1.31	1.35	1.00	0.95

	(1.04 to 1.61)	(1.11 to 1.64)	(0.82 to 1.22)	(0.71 to 1.27)
Australia	1.10 (0.82 to 1.44)	1.08 (0.82 to 1.41)	0.82 (0.62 to 1.08)	0.86 (0.58 to 1.23)
New Zealand	2.31 (1.75 to 2.95)	2.57 (1.91 to 3.23)	1.81 (1.40 to 2.23)	1.42 (1.01 to 1.91)
High-income Asia Pacific	1.17 (0.98 to 1.84)	1.32 (1.19 to 1.50)	1.54 (1.20 to 1.77)	1.22 (1.03 to 1.42)
Brunei Darussalam	0.70 (0.33 to 1.37)	0.46 (0.25 to 0.86)	0.35 (0.20 to 0.56)	0.42 (0.21 to 0.76)
Japan	0.96 (0.90 to 1.04)	1.33 (1.25 to 1.42)	1.49 (1.38 to 1.59)	1.30 (1.18 to 1.42)
Republic of Korea	1.63 (1.04 to 3.71)	1.30 (0.94 to 1.81)	1.70 (0.74 to 2.31)	1.12 (0.49 to 1.73)
Singapore	1.16 (0.79 to 1.62)	1.36 (0.94 to 1.87)	0.89 (0.60 to 1.27)	0.63 (0.40 to 0.94)
High-income North America	2.36 (2.24 to 2.48)	2.18 (2.08 to 2.29)	1.80 (1.69 to 1.90)	2.12 (1.95 to 2.29)
Canada	2.59 (2.08 to 3.15)	2.32 (1.90 to 2.80)	1.59 (1.27 to 1.94)	1.77 (1.22 to 2.43)
Greenland	18.96 (9.75 to 26.98)	14.17 (8.62 to 19.02)	11.36 (6.52 to 15.37)	8.82 (5.06 to 13.49)
United States of America	2.33 (2.21 to 2.45)	2.16 (2.06 to 2.26)	1.82 (1.71 to 1.92)	2.15 (2.00 to 2.33)
Southern Latin America	1.33 (1.12 to 1.58)	1.49 (1.25 to 1.77)	1.90 (1.56 to 2.27)	1.53 (1.11 to 2.04)
Argentina	1.18 (0.91 to 1.50)	1.54 (1.22 to 1.92)	2.03 (1.60 to 2.51)	1.67 (1.11 to 2.36)
Chile	1.68 (1.25 to 2.22)	1.36 (1.05 to 1.80)	1.57 (1.20 to 2.02)	1.15 (0.74 to 1.64)
Uruguay	1.62 (1.09 to 2.27)	1.60 (1.10 to 2.24)	1.72 (1.16 to 2.39)	1.51 (0.94 to 2.25)
Western Europe	1.02 (0.94 to 1.12)	0.90 (0.83 to 0.98)	0.67 (0.61 to 0.74)	0.55 (0.48 to 0.64)
Andorra	0.66 (0.32 to 1.15)	0.42 (0.22 to 0.73)	0.36 (0.21 to 0.57)	0.28 (0.15 to 0.45)
Austria	1.63 (1.18 to 2.18)	1.46 (1.06 to 1.96)	0.91 (0.67 to 1.21)	0.77 (0.50 to 1.10)
Belgium	1.60 (1.17 to 2.11)	1.57 (1.16 to 2.04)	1.36 (0.97 to 1.81)	0.98 (0.62 to 1.40)
Cyprus	0.49 (0.22 to 0.89)	0.59 (0.28 to 1.10)	0.14 (0.07 to 0.24)	0.27 (0.14 to 0.48)
Denmark	1.48 (1.05 to 2.01)	1.03 (0.71 to 1.44)	0.36 (0.24 to 0.51)	0.38 (0.23 to 0.56)
Finland	2.27 (1.66 to 2.97)	1.15 (0.83 to 1.58)	0.84 (0.57 to 1.15)	0.84 (0.54 to 1.21)
France	1.13 (0.88 to 1.41)	1.24 (0.98 to 1.51)	1.01 (0.80 to 1.27)	0.75 (0.50 to 1.10)
Germany	1.42 (1.12 to 1.76)	1.24 (1.00 to 1.52)	0.79 (0.61 to 1.00)	0.61 (0.40 to 0.86)
Greece	0.44 (0.30 to 0.62)	0.29 (0.19 to 0.44)	0.27 (0.18 to 0.40)	0.26 (0.16 to 0.41)
Iceland	1.57 (1.05 to 2.27)	0.63 (0.41 to 0.89)	0.82 (0.53 to 1.16)	0.64 (0.39 to 0.99)
Ireland	1.02 (0.70 to 1.44)	1.34 (0.93 to 1.78)	1.06 (0.74 to 1.44)	0.77 (0.50 to 1.17)
Israel	0.72 (0.50 to 1.01)	0.88 (0.61 to 1.23)	0.70 (0.49 to 0.98)	0.56 (0.35 to 0.82)
Italy	0.63 (0.56 to 0.70)	0.55 (0.49 to 0.61)	0.41 (0.36 to 0.45)	0.37 (0.32 to 0.44)
Luxembourg	0.98 (0.63 to 1.47)	1.17 (0.77 to 1.70)	0.94 (0.59 to 1.43)	0.54 (0.32 to 0.86)
Malta	0.29 (0.18 to 0.44)	0.30 (0.18 to 0.46)	0.61 (0.38 to 0.90)	0.42 (0.25 to 0.65)
Monaco	0.70 (0.36 to 1.25)	0.67 (0.36 to 1.15)	0.56 (0.31 to 0.90)	0.47 (0.26 to 0.83)
Netherlands	1.03 (0.74 to 1.36)	1.01 (0.74 to 1.34)	0.89 (0.65 to 1.18)	0.65 (0.41 to 0.93)
Norway	3.66 (3.34 to 4.01)	2.23 (1.94 to 2.58)	1.62 (1.45 to 1.79)	1.22 (1.06 to 1.40)
Portugal	1.00 (0.68 to 1.40)	0.52 (0.34 to 0.76)	0.44 (0.29 to 0.63)	0.28 (0.17 to 0.43)
San Marino	1.20 (0.65 to 1.99)	0.83 (0.47 to 1.34)	0.70 (0.40 to 1.17)	0.66 (0.37 to 1.05)
Spain	1.10 (0.84 to 1.42)	0.64 (0.47 to 0.84)	0.52 (0.37 to 0.69)	0.44 (0.29 to 0.63)
Sweden	0.91 (0.67 to 1.22)	0.82 (0.61 to 1.08)	0.78 (0.57 to 1.04)	0.57 (0.40 to 0.79)
Switzerland	1.55 (1.11 to 2.10)	1.48 (1.06 to 2.02)	0.88 (0.62 to 1.22)	0.78 (0.50 to 1.15)
United Kingdom	0.37	0.32	0.27	0.34

	(0.34 to 0.41)	(0.29 to 0.34)	(0.25 to 0.30)	(0.31 to 0.37)
Latin America and Caribbean	0.91 (0.81 to 1.03)	1.17 (1.07 to 1.26)	1.44 (1.25 to 1.57)	1.28 (1.07 to 1.51)
Andean Latin America	1.02 (0.72 to 1.78)	1.49 (0.94 to 1.93)	2.23 (0.84 to 3.00)	1.73 (0.62 to 2.62)
Bolivia (Plurinational State of)	1.57 (0.78 to 2.68)	1.81 (0.91 to 3.05)	1.64 (0.80 to 2.91)	1.40 (0.69 to 2.48)
Ecuador	1.41 (0.95 to 2.19)	2.76 (1.18 to 3.94)	5.33 (1.35 to 7.72)	4.18 (0.94 to 6.77)
Peru	0.68 (0.37 to 1.44)	0.78 (0.51 to 1.22)	0.76 (0.49 to 1.10)	0.52 (0.27 to 0.91)
Caribbean	1.09 (0.72 to 1.47)	0.98 (0.70 to 1.29)	0.90 (0.60 to 1.27)	0.93 (0.58 to 1.40)
Antigua and Barbuda	0.16 (0.10 to 0.22)	0.32 (0.21 to 0.45)	0.19 (0.13 to 0.27)	0.19 (0.12 to 0.27)
Bahamas	0.17 (0.11 to 0.27)	0.23 (0.15 to 0.35)	0.20 (0.12 to 0.30)	0.19 (0.10 to 0.31)
Barbados	0.43 (0.27 to 0.66)	0.30 (0.19 to 0.47)	0.40 (0.24 to 0.63)	0.28 (0.16 to 0.47)
Belize	0.59 (0.38 to 0.87)	0.81 (0.52 to 1.19)	0.62 (0.40 to 0.94)	0.52 (0.31 to 0.80)
Bermuda	0.21 (0.14 to 0.32)	0.20 (0.13 to 0.29)	0.19 (0.12 to 0.28)	0.17 (0.11 to 0.27)
Cuba	0.86 (0.61 to 1.20)	0.85 (0.59 to 1.16)	0.64 (0.44 to 0.89)	0.62 (0.36 to 0.98)
Dominica	0.38 (0.22 to 0.61)	0.47 (0.28 to 0.73)	0.60 (0.35 to 0.97)	0.47 (0.25 to 0.84)
Dominican Republic	0.62 (0.36 to 1.07)	0.63 (0.39 to 1.00)	0.65 (0.36 to 1.07)	0.54 (0.28 to 0.97)
Grenada	0.33 (0.20 to 0.51)	0.47 (0.28 to 0.70)	0.45 (0.28 to 0.66)	0.29 (0.17 to 0.46)
Guyana	2.97 (2.00 to 4.18)	3.15 (2.19 to 4.41)	3.45 (2.42 to 4.71)	2.68 (1.64 to 3.98)
Haiti	1.99 (0.55 to 3.45)	1.46 (0.50 to 2.61)	1.38 (0.50 to 2.58)	1.47 (0.55 to 2.66)
Jamaica	0.24 (0.15 to 0.35)	0.26 (0.17 to 0.39)	0.34 (0.22 to 0.52)	0.36 (0.20 to 0.58)
Puerto Rico	0.78 (0.52 to 1.14)	0.58 (0.39 to 0.84)	0.38 (0.25 to 0.57)	0.31 (0.19 to 0.49)
Saint Kitts and Nevis	0.41 (0.25 to 0.61)	0.39 (0.23 to 0.60)	0.60 (0.36 to 0.93)	0.25 (0.13 to 0.41)
Saint Lucia	0.58 (0.36 to 0.88)	0.54 (0.34 to 0.82)	0.49 (0.30 to 0.74)	0.46 (0.26 to 0.75)
Saint Vincent and the Grenadines	0.44 (0.26 to 0.70)	0.89 (0.54 to 1.36)	0.56 (0.35 to 0.83)	0.50 (0.28 to 0.82)
Suriname	1.79 (1.00 to 2.78)	2.19 (1.30 to 3.35)	1.39 (0.85 to 2.15)	2.12 (1.10 to 3.58)
Trinidad and Tobago	2.14 (1.42 to 3.03)	1.72 (1.15 to 2.44)	1.30 (0.86 to 1.91)	1.05 (0.59 to 1.79)
United States Virgin Islands	1.09 (0.61 to 1.85)	1.22 (0.72 to 1.89)	0.36 (0.21 to 0.59)	0.30 (0.16 to 0.55)
Central Latin America	1.01 (0.92 to 1.11)	1.51 (1.38 to 1.64)	1.75 (1.60 to 1.90)	1.48 (1.23 to 1.77)
Colombia	1.03 (0.80 to 1.31)	1.71 (1.33 to 2.11)	2.27 (1.80 to 2.79)	1.60 (0.94 to 2.52)
Costa Rica	1.22 (0.86 to 1.71)	1.15 (0.81 to 1.57)	1.33 (0.92 to 1.85)	1.30 (0.82 to 2.05)
El Salvador	1.54 (0.90 to 2.69)	1.13 (0.68 to 1.87)	1.02 (0.65 to 1.59)	0.93 (0.42 to 1.69)
Guatemala	0.68 (0.43 to 0.98)	0.84 (0.57 to 1.21)	0.65 (0.45 to 0.90)	0.53 (0.31 to 0.87)
Honduras	0.90 (0.44 to 2.06)	0.56 (0.23 to 1.50)	0.36 (0.15 to 1.03)	0.27 (0.11 to 0.79)
Mexico	0.93 (0.84 to 1.02)	1.57 (1.44 to 1.71)	2.10 (1.91 to 2.29)	1.87 (1.60 to 2.19)
Nicaragua	1.54 (0.84 to 2.44)	1.73 (1.02 to 2.50)	1.74 (0.95 to 2.44)	1.28 (0.73 to 2.13)
Panama	0.91 (0.57 to 1.35)	1.46 (0.97 to 2.08)	1.26 (0.86 to 1.81)	0.96 (0.57 to 1.55)
Venezuela (Bolivarian Republic of)	1.24 (0.93 to 1.61)	1.71 (1.35 to 2.16)	1.06 (0.78 to 1.38)	1.09 (0.61 to 1.76)
Tropical Latin America	0.72 (0.64 to 0.80)	0.71 (0.63 to 0.78)	0.91 (0.80 to 1.01)	0.95 (0.81 to 1.10)
Brazil	0.72 (0.64 to 0.80)	0.69 (0.61 to 0.77)	0.89 (0.78 to 0.99)	0.93 (0.80 to 1.07)
Paraguay	0.84 (0.48 to 1.28)	1.10 (0.49 to 1.65)	1.49 (0.48 to 2.24)	1.36 (0.43 to 2.33)
North Africa and Middle East	1.26 (0.56 to 1.73)	1.17 (0.51 to 1.54)	0.72 (0.37 to 0.93)	0.49 (0.28 to 0.66)
Afghanistan	1.44	1.38	1.05	0.80

	(0.32 to 2.75)	(0.31 to 2.50)	(0.27 to 1.92)	(0.22 to 1.53)
Algeria	1.65 (0.64 to 2.99)	1.10 (0.51 to 2.00)	0.67 (0.34 to 1.19)	0.50 (0.26 to 0.89)
Bahrain	0.46 (0.21 to 0.89)	0.37 (0.20 to 0.68)	0.39 (0.23 to 0.63)	0.29 (0.15 to 0.53)
Egypt	0.98 (0.37 to 1.78)	0.72 (0.31 to 1.34)	0.57 (0.26 to 1.08)	0.42 (0.20 to 0.79)
Iran (Islamic Republic of)	1.66 (0.47 to 2.26)	1.68 (0.46 to 2.16)	1.08 (0.38 to 1.37)	0.45 (0.23 to 0.58)
Iraq	0.69 (0.28 to 1.46)	0.63 (0.28 to 1.40)	0.42 (0.22 to 0.97)	0.26 (0.13 to 0.58)
Jordan	0.42 (0.19 to 0.77)	0.40 (0.20 to 0.75)	0.17 (0.09 to 0.32)	0.21 (0.11 to 0.40)
Kuwait	0.43 (0.25 to 0.72)	0.35 (0.22 to 0.52)	0.28 (0.18 to 0.41)	0.26 (0.15 to 0.43)
Lebanon	0.61 (0.27 to 1.09)	0.48 (0.22 to 0.93)	0.36 (0.17 to 0.72)	0.29 (0.14 to 0.60)
Libya	0.61 (0.31 to 1.06)	0.44 (0.22 to 0.79)	0.48 (0.24 to 0.86)	0.29 (0.15 to 0.53)
Morocco	1.04 (0.37 to 1.91)	1.52 (0.67 to 2.72)	0.88 (0.43 to 1.54)	0.53 (0.27 to 0.96)
Oman	0.93 (0.37 to 1.73)	0.75 (0.29 to 1.29)	0.61 (0.22 to 1.10)	0.53 (0.21 to 1.01)
Palestine	0.44 (0.20 to 0.79)	0.34 (0.18 to 0.55)	0.26 (0.15 to 0.42)	0.28 (0.13 to 0.49)
Qatar	0.67 (0.32 to 1.21)	0.55 (0.29 to 0.98)	0.49 (0.29 to 0.82)	0.31 (0.16 to 0.59)
Saudi Arabia	0.28 (0.13 to 0.51)	0.23 (0.13 to 0.38)	0.19 (0.11 to 0.30)	0.13 (0.07 to 0.24)
Sudan	1.22 (0.34 to 2.58)	1.16 (0.37 to 2.31)	0.97 (0.35 to 2.02)	0.80 (0.35 to 1.52)
Syrian Arab Republic	0.39 (0.18 to 0.76)	0.30 (0.17 to 0.59)	0.28 (0.15 to 0.49)	0.28 (0.13 to 0.53)
Tunisia	0.90 (0.44 to 1.63)	0.70 (0.33 to 1.32)	0.53 (0.26 to 0.98)	0.39 (0.19 to 0.73)
Turkey	1.97 (0.63 to 3.59)	2.11 (0.62 to 3.64)	0.87 (0.35 to 1.28)	0.54 (0.27 to 0.86)
United Arab Emirates	0.72 (0.36 to 1.32)	0.74 (0.39 to 1.32)	0.37 (0.20 to 0.62)	0.27 (0.14 to 0.56)
Yemen	1.10 (0.32 to 2.21)	1.02 (0.41 to 1.96)	0.79 (0.35 to 1.57)	0.71 (0.32 to 1.31)
South Asia	3.15 (1.88 to 4.21)	3.32 (2.17 to 4.21)	2.79 (2.01 to 3.60)	2.05 (1.48 to 2.79)
Bangladesh	6.55 (2.06 to 11.60)	5.95 (2.67 to 9.36)	5.93 (3.28 to 8.97)	3.61 (2.03 to 5.93)
Bhutan	0.70 (0.11 to 1.93)	0.83 (0.34 to 2.31)	0.79 (0.33 to 2.17)	0.75 (0.29 to 2.14)
India	2.84 (1.75 to 3.78)	3.07 (2.05 to 3.98)	2.44 (1.73 to 3.10)	1.73 (1.17 to 2.47)
Nepal	3.13 (1.18 to 7.35)	2.01 (0.87 to 4.91)	1.74 (0.79 to 4.35)	1.60 (0.73 to 3.79)
Pakistan	2.11 (1.05 to 3.76)	2.81 (1.42 to 4.98)	2.90 (1.39 to 5.21)	2.87 (1.45 to 5.13)
Southeast Asia, East Asia, and Oceania	2.60 (1.39 to 3.16)	1.85 (1.17 to 2.17)	1.19 (0.91 to 1.41)	0.79 (0.60 to 0.99)
East Asia	3.20 (1.74 to 3.90)	2.18 (1.41 to 2.59)	1.53 (1.21 to 1.85)	0.95 (0.72 to 1.22)
China	3.26 (1.77 to 3.98)	2.21 (1.41 to 2.61)	1.54 (1.22 to 1.86)	0.95 (0.71 to 1.22)
Democratic People's Republic of Korea	2.67 (1.28 to 4.77)	2.12 (1.02 to 3.70)	1.65 (0.84 to 2.78)	1.23 (0.63 to 2.15)
Taiwan, Province of China	0.51 (0.37 to 0.71)	0.47 (0.33 to 0.64)	0.53 (0.38 to 0.73)	0.49 (0.29 to 0.78)
Oceania	1.92 (1.27 to 3.13)	1.86 (1.26 to 3.16)	1.55 (1.04 to 2.79)	1.41 (0.95 to 2.58)
American Samoa	1.53 (0.81 to 2.50)	1.39 (0.84 to 2.11)	1.28 (0.76 to 2.04)	1.18 (0.62 to 2.10)
Cook Islands	4.38 (2.45 to 6.72)	3.34 (2.00 to 5.30)	1.92 (1.14 to 3.16)	1.48 (0.80 to 2.54)
Fiji	2.69 (1.44 to 4.42)	3.54 (2.01 to 5.47)	2.83 (1.70 to 4.23)	2.98 (1.54 to 5.09)
Guam	5.23 (3.12 to 7.82)	6.68 (4.17 to 9.63)	3.76 (2.48 to 5.40)	3.25 (1.92 to 5.16)
Kiribati	9.22 (5.20 to 14.40)	8.80 (5.27 to 14.07)	7.49 (4.11 to 12.23)	6.63 (3.58 to 11.02)
Marshall Islands	3.52 (1.79 to 5.74)	5.10 (2.55 to 8.57)	4.70 (2.23 to 7.90)	3.97 (2.10 to 6.50)
Micronesia (Federated States of)	5.38 (2.72 to 9.05)	4.67 (2.34 to 7.79)	4.03 (2.25 to 6.53)	3.39 (0.91 to 5.68)
Nauru	5.32	6.98	6.76	4.90

	(2.82 to 8.58)	(3.11 to 11.59)	(2.97 to 11.43)	(2.39 to 8.30)
Niue	3.97 (2.22 to 6.54)	4.27 (2.29 to 7.05)	3.93 (2.01 to 6.70)	3.60 (1.92 to 6.20)
Northern Mariana Islands	2.64 (1.43 to 4.27)	2.16 (1.32 to 3.39)	2.61 (1.58 to 3.98)	3.27 (1.91 to 5.17)
Palau	2.69 (1.38 to 4.67)	1.12 (0.61 to 1.91)	1.98 (1.13 to 3.43)	1.73 (0.97 to 2.91)
Papua New Guinea	0.85 (0.35 to 2.53)	0.78 (0.31 to 2.41)	0.78 (0.29 to 2.30)	0.73 (0.30 to 2.18)
Samoa	4.45 (2.22 to 7.17)	3.58 (1.87 to 5.88)	3.01 (1.58 to 5.00)	2.47 (1.33 to 4.07)
Solomon Islands	6.40 (2.74 to 11.08)	6.71 (2.84 to 11.39)	6.59 (2.85 to 11.48)	5.85 (2.62 to 10.00)
Tokelau	2.88 (1.64 to 4.66)	2.58 (1.42 to 4.37)	2.03 (1.13 to 3.48)	1.55 (0.82 to 2.79)
Tonga	0.93 (0.48 to 1.57)	1.20 (0.64 to 2.05)	1.00 (0.55 to 1.74)	0.90 (0.48 to 1.54)
Tuvalu	6.77 (3.01 to 11.32)	4.95 (2.47 to 7.98)	3.70 (1.93 to 6.20)	2.82 (1.59 to 4.70)
Vanuatu	4.19 (2.02 to 7.11)	4.58 (2.19 to 7.81)	4.48 (2.15 to 7.69)	3.97 (2.01 to 6.66)
Southeast Asia	1.43 (0.68 to 1.91)	1.08 (0.56 to 1.38)	0.70 (0.43 to 0.87)	0.57 (0.38 to 0.71)
Cambodia	2.04 (0.69 to 3.83)	1.64 (0.57 to 2.89)	1.14 (0.43 to 2.04)	0.88 (0.35 to 1.62)
Indonesia	0.89 (0.44 to 1.23)	0.73 (0.38 to 0.96)	0.55 (0.30 to 0.70)	0.42 (0.26 to 0.57)
Lao People's Democratic Republic	2.48 (0.70 to 4.56)	1.77 (0.59 to 3.21)	1.26 (0.54 to 2.19)	0.89 (0.42 to 1.61)
Malaysia	0.36 (0.16 to 0.88)	0.27 (0.14 to 0.71)	0.22 (0.12 to 0.61)	0.23 (0.10 to 0.61)
Maldives	0.82 (0.24 to 1.65)	0.43 (0.18 to 0.81)	0.29 (0.15 to 0.51)	0.26 (0.13 to 0.47)
Mauritius	1.39 (0.93 to 2.00)	1.21 (0.78 to 1.78)	0.75 (0.49 to 1.11)	0.52 (0.30 to 0.83)
Myanmar	2.70 (0.69 to 5.63)	2.20 (0.65 to 4.27)	1.37 (0.47 to 2.52)	0.99 (0.39 to 1.70)
Philippines	0.98 (0.57 to 1.24)	0.78 (0.48 to 0.98)	0.67 (0.38 to 0.80)	0.65 (0.35 to 0.84)
Seychelles	0.33 (0.17 to 0.64)	0.14 (0.06 to 0.29)	0.35 (0.19 to 0.61)	0.40 (0.19 to 0.76)
Sri Lanka	4.43 (2.26 to 6.98)	2.45 (1.66 to 3.39)	1.24 (0.78 to 2.09)	1.06 (0.52 to 1.97)
Thailand	2.85 (1.09 to 4.85)	2.31 (0.97 to 3.69)	0.82 (0.52 to 1.30)	0.57 (0.32 to 1.05)
Timor-Leste	1.09 (0.38 to 2.14)	0.89 (0.40 to 1.61)	0.66 (0.30 to 1.15)	0.68 (0.31 to 1.21)
Viet Nam	0.94 (0.41 to 1.66)	0.76 (0.40 to 1.30)	0.56 (0.32 to 0.90)	0.54 (0.28 to 0.99)
Sub-Saharan Africa	2.63 (1.68 to 3.32)	2.42 (1.65 to 3.07)	2.18 (1.55 to 2.69)	1.85 (1.31 to 2.46)
Central Sub-Saharan Africa	3.28 (2.00 to 4.95)	3.02 (1.82 to 4.81)	2.74 (1.68 to 4.39)	2.19 (1.27 to 4.03)
Angola	4.18 (1.77 to 7.57)	3.58 (1.43 to 6.39)	3.12 (1.69 to 5.26)	2.28 (1.20 to 4.01)
Central African Republic	4.82 (2.37 to 8.21)	4.13 (1.95 to 7.27)	4.27 (2.17 to 7.44)	3.84 (1.92 to 6.79)
Congo	2.78 (0.95 to 5.29)	2.61 (1.15 to 4.57)	2.10 (1.03 to 3.86)	1.65 (0.78 to 3.12)
Democratic Republic of the Congo	2.97 (1.47 to 5.04)	2.85 (1.36 to 5.20)	2.58 (1.30 to 4.53)	2.12 (0.99 to 4.37)
Equatorial Guinea	4.42 (2.13 to 8.11)	2.77 (1.29 to 5.10)	1.63 (0.73 to 3.20)	1.25 (0.52 to 2.69)
Gabon	2.92 (1.20 to 5.38)	2.25 (1.06 to 4.16)	2.20 (1.15 to 3.90)	1.71 (0.85 to 3.27)
Eastern Sub-Saharan Africa	3.52 (1.98 to 4.65)	3.04 (1.83 to 3.86)	2.48 (1.67 to 3.06)	2.05 (1.36 to 2.79)
Burundi	4.80 (2.44 to 8.53)	4.10 (2.01 to 7.22)	3.41 (1.72 to 5.94)	2.88 (1.44 to 5.23)
Comoros	2.44 (0.45 to 4.93)	2.15 (0.74 to 4.12)	1.33 (0.53 to 2.58)	1.66 (0.68 to 3.39)
Djibouti	2.02 (0.83 to 3.84)	2.60 (1.02 to 5.11)	2.29 (0.94 to 4.50)	2.01 (0.82 to 3.97)
Eritrea	3.19 (1.36 to 6.15)	3.24 (1.36 to 6.26)	2.88 (1.27 to 5.28)	2.45 (1.01 to 4.70)
Ethiopia	4.53 (2.00 to 6.95)	3.42 (1.56 to 5.05)	2.13 (1.20 to 2.96)	1.65 (0.97 to 2.45)
Kenya	1.58 (0.93 to 2.16)	1.55 (0.94 to 2.07)	1.92 (1.20 to 2.51)	1.52 (0.93 to 2.37)
Madagascar	5.37	2.85	2.33	1.90

	(1.82 to 9.86)	(1.30 to 4.90)	(1.21 to 3.89)	(0.98 to 3.37)
Malawi	4.16 (1.91 to 7.13)	4.23 (2.30 to 7.05)	3.39 (1.91 to 5.48)	2.61 (1.32 to 4.67)
Mozambique	3.22 (1.44 to 5.62)	2.81 (1.42 to 4.76)	3.37 (1.80 to 5.74)	2.55 (1.22 to 4.79)
Rwanda	4.73 (2.30 to 8.54)	4.30 (2.14 to 7.37)	2.41 (1.24 to 4.44)	2.04 (1.03 to 3.67)
Somalia	2.25 (0.86 to 4.29)	3.28 (1.26 to 6.44)	3.49 (1.41 to 6.66)	3.10 (1.28 to 5.89)
South Sudan	1.57 (0.71 to 2.82)	1.98 (0.86 to 3.70)	1.63 (0.79 to 3.08)	1.80 (0.86 to 3.60)
Uganda	2.15 (0.72 to 4.43)	3.20 (1.36 to 5.78)	2.33 (1.08 to 4.19)	2.04 (0.93 to 3.61)
United Republic of Tanzania	3.68 (1.82 to 6.62)	2.71 (1.56 to 4.27)	2.46 (1.35 to 4.12)	2.22 (1.17 to 3.75)
Zambia	3.36 (1.58 to 6.08)	3.89 (2.02 to 6.52)	3.60 (2.02 to 6.08)	2.45 (1.28 to 4.36)
Southern Sub-Saharan Africa	0.45 (0.33 to 0.64)	0.55 (0.43 to 0.76)	0.51 (0.37 to 0.70)	0.33 (0.19 to 0.53)
Botswana	0.64 (0.34 to 1.15)	0.79 (0.40 to 1.35)	0.61 (0.31 to 1.12)	0.64 (0.32 to 1.13)
Eswatini	0.77 (0.39 to 1.36)	0.84 (0.43 to 1.47)	0.90 (0.45 to 1.58)	0.79 (0.42 to 1.38)
Lesotho	0.89 (0.43 to 1.61)	0.91 (0.44 to 1.67)	1.08 (0.54 to 2.00)	1.00 (0.49 to 1.75)
Namibia	0.53 (0.19 to 0.96)	0.55 (0.26 to 0.97)	0.58 (0.30 to 1.00)	0.48 (0.23 to 0.87)
South Africa	0.35 (0.26 to 0.56)	0.55 (0.43 to 0.83)	0.38 (0.29 to 0.54)	0.07 (0.03 to 0.17)
Zimbabwe	0.63 (0.28 to 1.20)	0.42 (0.17 to 0.83)	0.72 (0.34 to 1.31)	0.82 (0.37 to 1.57)
Western Sub-Saharan Africa	2.05 (1.23 to 2.81)	2.05 (1.32 to 2.81)	2.00 (1.40 to 2.64)	1.77 (1.22 to 2.38)
Benin	2.56 (1.27 to 4.36)	2.69 (1.31 to 4.54)	2.55 (1.30 to 4.28)	2.24 (1.07 to 4.27)
Burkina Faso	2.67 (1.15 to 4.92)	2.62 (1.21 to 4.55)	2.65 (1.26 to 4.78)	2.63 (1.08 to 5.11)
Cabo Verde	1.88 (0.97 to 3.21)	2.15 (1.14 to 3.70)	1.64 (0.93 to 2.67)	1.58 (0.84 to 2.67)
Cameroon	2.08 (0.96 to 3.60)	2.84 (1.37 to 4.96)	2.88 (1.51 to 4.86)	2.34 (1.19 to 4.24)
Chad	2.31 (1.01 to 4.15)	2.99 (1.31 to 5.41)	3.08 (1.40 to 5.63)	2.91 (1.39 to 5.32)
Côte d'Ivoire	3.52 (1.75 to 6.23)	3.85 (2.03 to 6.74)	3.43 (1.83 to 5.77)	2.77 (1.37 to 5.06)
Gambia	1.64 (0.64 to 3.21)	1.78 (0.74 to 3.41)	1.72 (0.69 to 3.35)	1.46 (0.62 to 2.80)
Ghana	1.78 (0.92 to 3.07)	1.41 (0.78 to 2.56)	2.14 (1.29 to 3.41)	1.85 (0.95 to 3.29)
Guinea	2.31 (0.95 to 4.26)	2.29 (1.03 to 4.21)	2.90 (1.24 to 5.14)	2.64 (1.19 to 4.82)
Guinea-Bissau	3.93 (1.63 to 7.46)	3.89 (1.73 to 7.08)	3.44 (1.62 to 6.05)	2.66 (1.27 to 4.79)
Liberia	2.41 (1.15 to 4.26)	2.13 (1.02 to 3.69)	1.93 (0.94 to 3.36)	1.68 (0.82 to 2.96)
Mali	2.24 (0.92 to 4.34)	2.05 (0.84 to 3.69)	1.95 (0.84 to 3.65)	2.02 (0.86 to 3.79)
Mauritania	1.78 (0.77 to 3.30)	1.59 (0.72 to 2.94)	1.33 (0.60 to 2.43)	1.11 (0.45 to 2.48)
Niger	2.65 (0.92 to 5.02)	2.40 (0.98 to 4.45)	1.93 (0.84 to 3.62)	2.03 (0.88 to 3.99)
Nigeria	1.50 (0.63 to 2.54)	1.49 (0.69 to 2.51)	1.39 (0.76 to 2.29)	1.19 (0.71 to 1.96)
Sao Tome and Principe	0.84 (0.44 to 1.47)	0.46 (0.23 to 0.81)	0.38 (0.19 to 0.69)	0.33 (0.16 to 0.66)
Senegal	2.99 (1.43 to 5.20)	2.48 (1.20 to 4.28)	2.44 (1.34 to 4.14)	2.22 (1.10 to 3.95)
Sierra Leone	2.41 (0.93 to 4.59)	2.47 (1.02 to 4.71)	2.43 (1.04 to 4.39)	2.26 (1.01 to 4.24)
Togo	2.30 (1.16 to 4.01)	2.56 (1.22 to 4.42)	2.46 (1.26 to 4.19)	2.11 (1.02 to 3.79)
Africa	2.30 (1.45 to 2.91)	2.13 (1.43 to 2.68)	1.94 (1.37 to 2.39)	1.65 (1.16 to 2.16)
America	1.34 (1.27 to 1.43)	1.49 (1.41 to 1.56)	1.58 (1.45 to 1.67)	1.56 (1.41 to 1.74)
Asia	2.70 (1.63 to 3.25)	2.45 (1.65 to 2.89)	2.01 (1.50 to 2.45)	1.48 (1.12 to 1.90)
Europe	2.33 (2.15 to 2.55)	2.68 (2.49 to 2.89)	1.44 (1.35 to 1.54)	1.15 (1.02 to 1.31)

Supplementary Table 2. Deaths due to suicide among girls aged 10-14 years: country/territory-wise comparison of death rates (per 100,000 individuals) together with 95% uncertainty intervals (UIs)– 1990-2019

Location	Death rate (95% UI) 1990	2000	2010	2019
Global	2.23 (1.93 to 2.57)	2.11 (1.87 to 2.38)	1.34 (1.16 to 1.51)	1.07 (0.92 to 1.24)
Central Europe, Eastern Europe, and Central Asia	1.02 (0.97 to 1.08)	1.24 (1.17 to 1.31)	1.38 (1.28 to 1.49)	1.22 (1.06 to 1.40)
Central Asia	1.17 (1.01 to 1.35)	1.37 (1.21 to 1.57)	1.90 (1.60 to 2.20)	1.83 (1.43 to 2.33)
Armenia	0.21 (0.14 to 0.30)	0.14 (0.09 to 0.22)	0.42 (0.30 to 0.59)	0.32 (0.20 to 0.48)
Azerbaijan	0.21 (0.13 to 0.32)	0.17 (0.10 to 0.27)	0.25 (0.13 to 0.43)	0.20 (0.10 to 0.35)
Georgia	0.40 (0.25 to 0.59)	0.29 (0.18 to 0.45)	0.40 (0.24 to 0.60)	0.25 (0.14 to 0.40)
Kazakhstan	1.56 (1.20 to 1.97)	2.51 (2.00 to 3.13)	2.97 (2.35 to 3.67)	2.16 (1.53 to 2.92)
Kyrgyzstan	1.98 (1.42 to 2.56)	1.36 (0.99 to 1.81)	1.31 (0.95 to 1.72)	1.28 (0.86 to 1.77)
Mongolia	4.47 (2.57 to 7.19)	3.06 (1.64 to 4.81)	3.53 (2.12 to 5.65)	2.70 (1.55 to 4.47)
Tajikistan	0.71 (0.47 to 1.06)	0.62 (0.39 to 0.94)	0.56 (0.30 to 0.92)	0.66 (0.34 to 1.14)
Turkmenistan	1.05 (0.73 to 1.42)	1.35 (0.98 to 1.81)	1.06 (0.75 to 1.45)	0.83 (0.54 to 1.23)
Uzbekistan	1.07 (0.83 to 1.35)	1.45 (1.13 to 1.84)	2.59 (1.99 to 3.28)	2.76 (1.87 to 3.81)
Central Europe	0.76 (0.68 to 0.83)	0.61 (0.56 to 0.67)	0.55 (0.50 to 0.61)	0.39 (0.33 to 0.46)
Albania	0.79 (0.50 to 1.17)	0.89 (0.59 to 1.28)	1.39 (0.85 to 2.14)	1.03 (0.58 to 1.67)
Bosnia and Herzegovina	1.09 (0.73 to 1.58)	0.60 (0.35 to 0.96)	0.49 (0.29 to 0.72)	0.69 (0.41 to 1.06)
Bulgaria	1.87 (1.38 to 2.46)	1.38 (1.00 to 1.84)	0.95 (0.67 to 1.33)	0.60 (0.38 to 0.90)
Croatia	0.98 (0.70 to 1.33)	0.74 (0.51 to 1.01)	0.60 (0.43 to 0.84)	0.43 (0.28 to 0.63)
Czechia	0.56 (0.39 to 0.76)	0.52 (0.37 to 0.71)	0.33 (0.23 to 0.45)	0.22 (0.14 to 0.34)
Hungary	0.89 (0.64 to 1.21)	0.55 (0.37 to 0.76)	0.33 (0.23 to 0.47)	0.26 (0.17 to 0.40)
Montenegro	1.14 (0.69 to 1.78)	1.40 (0.89 to 2.17)	0.94 (0.58 to 1.36)	0.49 (0.28 to 0.82)
North Macedonia	1.49 (0.94 to 2.15)	1.06 (0.70 to 1.54)	0.63 (0.40 to 0.92)	0.65 (0.37 to 1.01)
Poland	0.58 (0.51 to 0.64)	0.52 (0.47 to 0.58)	0.59 (0.53 to 0.66)	0.41 (0.34 to 0.50)
Romania	0.48 (0.35 to 0.65)	0.49 (0.35 to 0.65)	0.51 (0.36 to 0.69)	0.38 (0.25 to 0.55)
Serbia	0.98 (0.55 to 1.55)	0.61 (0.40 to 0.90)	0.30 (0.19 to 0.44)	0.19 (0.11 to 0.32)
Slovakia	0.62 (0.37 to 0.96)	0.45 (0.29 to 0.65)	0.43 (0.29 to 0.64)	0.32 (0.18 to 0.50)
Slovenia	1.02 (0.69 to 1.42)	0.86 (0.62 to 1.18)	0.48 (0.35 to 0.66)	0.48 (0.32 to 0.69)
Eastern Europe	1.12 (1.05 to 1.19)	1.48 (1.40 to 1.57)	1.51 (1.43 to 1.60)	1.24 (1.09 to 1.42)
Belarus	1.11 (0.82 to 1.47)	1.06 (0.77 to 1.45)	0.91 (0.65 to 1.22)	0.68 (0.42 to 1.00)
Estonia	1.22 (0.86 to 1.71)	0.82 (0.59 to 1.14)	0.45 (0.31 to 0.63)	0.34 (0.22 to 0.52)
Latvia	1.30 (0.90 to 1.80)	0.92 (0.65 to 1.27)	0.64 (0.44 to 0.92)	0.39 (0.24 to 0.61)
Lithuania	1.26 (0.90 to 1.74)	1.18 (0.82 to 1.63)	0.91 (0.65 to 1.22)	0.82 (0.55 to 1.17)
Republic of Moldova	1.17 (0.82 to 1.57)	0.73 (0.50 to 1.03)	0.83 (0.57 to 1.15)	0.60 (0.40 to 0.86)
Russian Federation	1.22 (1.15 to 1.29)	1.67 (1.59 to 1.76)	1.84 (1.74 to 1.95)	1.30 (1.13 to 1.53)
Ukraine	0.80 (0.65 to 1.00)	1.10 (0.86 to 1.38)	0.79 (0.61 to 1.00)	1.28 (0.85 to 1.79)
High-income	0.67 (0.63 to 0.70)	0.70 (0.66 to 0.73)	0.75 (0.70 to 0.79)	0.74 (0.69 to 0.81)
Australasia	0.52	0.70	0.72	0.71

	(0.41 to 0.65)	(0.58 to 0.85)	(0.60 to 0.88)	(0.54 to 0.91)
Australia	0.42<br>(0.31 to 0.57)	0.51<br>(0.38 to 0.68)	0.55<br>(0.42 to 0.71)	0.61<br>(0.43 to 0.85)
New Zealand	0.98<br>(0.75 to 1.26)	1.58<br>(1.24 to 1.95)	1.53<br>(1.19 to 1.84)	1.18<br>(0.89 to 1.50)
High-income Asia Pacific	0.79<br>(0.69 to 0.92)	0.89<br>(0.78 to 1.01)	1.08<br>(0.95 to 1.22)	0.75<br>(0.65 to 0.87)
Brunei Darussalam	0.23<br>(0.13 to 0.38)	0.24<br>(0.15 to 0.36)	0.36<br>(0.24 to 0.56)	0.31<br>(0.18 to 0.49)
Japan	0.60<br>(0.56 to 0.64)	0.62<br>(0.57 to 0.66)	0.66<br>(0.60 to 0.71)	0.65<br>(0.59 to 0.70)
Republic of Korea	1.16<br>(0.88 to 1.50)	1.48<br>(1.14 to 1.85)	1.92<br>(1.55 to 2.32)	1.01<br>(0.70 to 1.39)
Singapore	1.37<br>(1.00 to 1.85)	1.19<br>(0.83 to 1.59)	0.71<br>(0.51 to 0.95)	0.80<br>(0.55 to 1.10)
High-income North America	0.87<br>(0.82 to 0.93)	0.83<br>(0.77 to 0.89)	0.92<br>(0.86 to 0.99)	1.12<br>(1.03 to 1.23)
Canada	1.11<br>(0.87 to 1.39)	1.44<br>(1.14 to 1.75)	1.33<br>(1.07 to 1.60)	1.58<br>(1.16 to 2.04)
Greenland	13.83<br>(8.49 to 18.90)	30.41<br>(21.10 to 39.66)	7.93<br>(5.71 to 10.24)	6.10<br>(3.77 to 8.63)
United States of America	0.85<br>(0.79 to 0.90)	0.76<br>(0.70 to 0.81)	0.88<br>(0.82 to 0.94)	1.08<br>(0.99 to 1.18)
Southern Latin America	0.82<br>(0.68 to 0.98)	1.17<br>(0.99 to 1.38)	1.43<br>(1.18 to 1.69)	1.21<br>(0.91 to 1.55)
Argentina	0.80<br>(0.62 to 1.00)	1.31<br>(1.06 to 1.60)	1.55<br>(1.23 to 1.90)	1.29<br>(0.90 to 1.75)
Chile	0.90<br>(0.66 to 1.20)	0.84<br>(0.64 to 1.09)	1.17<br>(0.88 to 1.49)	1.05<br>(0.73 to 1.41)
Uruguay	0.74<br>(0.53 to 1.03)	1.09<br>(0.79 to 1.50)	1.02<br>(0.73 to 1.40)	0.89<br>(0.60 to 1.28)
Western Europe	0.42<br>(0.38 to 0.45)	0.38<br>(0.35 to 0.42)	0.28<br>(0.25 to 0.32)	0.26<br>(0.23 to 0.30)
Andorra	0.39<br>(0.20 to 0.69)	0.27<br>(0.14 to 0.45)	0.23<br>(0.13 to 0.38)	0.19<br>(0.10 to 0.32)
Austria	0.69<br>(0.49 to 0.93)	0.75<br>(0.55 to 1.01)	0.45<br>(0.32 to 0.63)	0.43<br>(0.29 to 0.61)
Belgium	0.57<br>(0.42 to 0.77)	0.61<br>(0.43 to 0.80)	0.44<br>(0.31 to 0.59)	0.35<br>(0.23 to 0.51)
Cyprus	0.14<br>(0.08 to 0.24)	0.10<br>(0.06 to 0.15)	0.08<br>(0.05 to 0.13)	0.09<br>(0.05 to 0.15)
Denmark	0.44<br>(0.31 to 0.62)	0.23<br>(0.16 to 0.32)	0.13<br>(0.09 to 0.19)	0.11<br>(0.07 to 0.17)
Finland	0.58<br>(0.41 to 0.79)	0.81<br>(0.57 to 1.10)	0.54<br>(0.38 to 0.73)	0.47<br>(0.32 to 0.67)
France	0.44<br>(0.34 to 0.58)	0.47<br>(0.36 to 0.60)	0.38<br>(0.28 to 0.50)	0.31<br>(0.21 to 0.43)
Germany	0.48<br>(0.37 to 0.62)	0.47<br>(0.35 to 0.60)	0.36<br>(0.27 to 0.48)	0.37<br>(0.26 to 0.52)
Greece	0.14<br>(0.10 to 0.20)	0.11<br>(0.07 to 0.15)	0.10<br>(0.07 to 0.15)	0.09<br>(0.06 to 0.13)
Iceland	0.46<br>(0.32 to 0.66)	0.32<br>(0.21 to 0.44)	0.22<br>(0.15 to 0.32)	0.19<br>(0.12 to 0.29)
Ireland	0.40<br>(0.27 to 0.55)	0.49<br>(0.34 to 0.68)	0.30<br>(0.21 to 0.43)	0.25<br>(0.16 to 0.36)
Israel	0.42<br>(0.29 to 0.57)	0.36<br>(0.26 to 0.49)	0.25<br>(0.18 to 0.34)	0.23<br>(0.15 to 0.32)
Italy	0.27<br>(0.24 to 0.30)	0.24<br>(0.22 to 0.27)	0.15<br>(0.13 to 0.16)	0.13<br>(0.11 to 0.15)
Luxembourg	0.71<br>(0.49 to 1.04)	0.37<br>(0.25 to 0.53)	0.28<br>(0.19 to 0.39)	0.21<br>(0.13 to 0.33)
Malta	0.15<br>(0.11 to 0.22)	0.19<br>(0.13 to 0.28)	0.11<br>(0.07 to 0.16)	0.10<br>(0.06 to 0.15)
Monaco	0.58<br>(0.30 to 1.00)	0.46<br>(0.24 to 0.78)	0.40<br>(0.22 to 0.68)	0.33<br>(0.18 to 0.55)
Netherlands	0.54<br>(0.38 to 0.72)	0.33<br>(0.24 to 0.46)	0.26<br>(0.19 to 0.35)	0.30<br>(0.20 to 0.43)
Norway	1.45<br>(1.29 to 1.60)	0.95<br>(0.86 to 1.06)	0.79<br>(0.71 to 0.87)	0.68<br>(0.60 to 0.78)
Portugal	0.66<br>(0.46 to 0.91)	0.42<br>(0.30 to 0.59)	0.22<br>(0.16 to 0.31)	0.18<br>(0.12 to 0.27)
San Marino	0.34<br>(0.18 to 0.58)	0.24<br>(0.13 to 0.39)	0.23<br>(0.12 to 0.38)	0.22<br>(0.11 to 0.36)
Spain	0.35<br>(0.26 to 0.47)	0.25<br>(0.18 to 0.35)	0.17<br>(0.12 to 0.23)	0.17<br>(0.11 to 0.25)
Sweden	0.90<br>(0.66 to 1.16)	0.70<br>(0.53 to 0.90)	0.66<br>(0.50 to 0.84)	0.66<br>(0.50 to 0.85)
Switzerland	0.74<br>(0.54 to 1.00)	0.76<br>(0.55 to 1.04)	0.39<br>(0.27 to 0.53)	0.42<br>(0.28 to 0.60)
United Kingdom	0.24	0.18	0.17	0.16

	(0.22 to 0.27)	(0.17 to 0.19)	(0.15 to 0.18)	(0.15 to 0.18)
Latin America and Caribbean	0.87 (0.81 to 0.94)	1.11 (1.03 to 1.20)	1.30 (1.21 to 1.41)	1.19 (1.04 to 1.36)
Andean Latin America	1.22 (0.93 to 1.60)	1.80 (1.42 to 2.26)	2.15 (1.72 to 2.67)	1.72 (1.27 to 2.29)
Bolivia (Plurinational State of)	1.98 (1.03 to 3.44)	2.45 (1.28 to 4.04)	2.08 (1.09 to 3.51)	1.58 (0.81 to 2.73)
Ecuador	1.56 (1.11 to 2.09)	3.38 (2.49 to 4.38)	4.46 (3.43 to 5.66)	3.80 (2.60 to 5.28)
Peru	0.84 (0.53 to 1.28)	0.82 (0.55 to 1.18)	0.84 (0.54 to 1.20)	0.58 (0.30 to 0.98)
Caribbean	1.73 (1.38 to 2.22)	1.15 (0.86 to 1.58)	1.04 (0.76 to 1.39)	1.04 (0.70 to 1.50)
Antigua and Barbuda	0.14 (0.08 to 0.20)	0.10 (0.06 to 0.16)	0.07 (0.04 to 0.12)	0.07 (0.04 to 0.12)
Bahamas	0.17 (0.11 to 0.24)	0.14 (0.09 to 0.20)	0.09 (0.06 to 0.13)	0.09 (0.06 to 0.15)
Barbados	0.49 (0.34 to 0.70)	0.27 (0.18 to 0.39)	0.24 (0.16 to 0.34)	0.22 (0.14 to 0.35)
Belize	0.74 (0.48 to 1.04)	0.73 (0.50 to 1.03)	0.36 (0.25 to 0.52)	0.26 (0.17 to 0.39)
Bermuda	0.42 (0.27 to 0.63)	0.26 (0.18 to 0.38)	0.16 (0.10 to 0.23)	0.13 (0.08 to 0.20)
Cuba	2.79 (2.13 to 3.47)	1.22 (0.92 to 1.60)	0.80 (0.58 to 1.08)	0.68 (0.45 to 0.98)
Dominica	0.44 (0.27 to 0.71)	0.43 (0.26 to 0.69)	0.37 (0.22 to 0.60)	0.36 (0.20 to 0.62)
Dominican Republic	0.63 (0.39 to 0.95)	0.53 (0.34 to 0.83)	0.61 (0.36 to 0.93)	0.54 (0.28 to 0.93)
Grenada	0.60 (0.38 to 0.87)	0.28 (0.18 to 0.41)	0.22 (0.15 to 0.32)	0.23 (0.15 to 0.33)
Guyana	4.06 (2.58 to 5.99)	2.46 (1.55 to 3.51)	3.42 (2.11 to 4.86)	2.26 (1.32 to 3.59)
Haiti	2.50 (1.32 to 4.45)	1.97 (1.04 to 3.38)	1.73 (0.89 to 2.97)	1.76 (0.91 to 3.06)
Jamaica	0.17 (0.11 to 0.24)	0.14 (0.10 to 0.21)	0.32 (0.21 to 0.46)	0.30 (0.18 to 0.45)
Puerto Rico	0.29 (0.20 to 0.40)	0.20 (0.14 to 0.28)	0.17 (0.12 to 0.24)	0.12 (0.07 to 0.18)
Saint Kitts and Nevis	0.60 (0.41 to 0.86)	0.36 (0.24 to 0.52)	0.22 (0.14 to 0.33)	0.19 (0.12 to 0.30)
Saint Lucia	0.30 (0.20 to 0.45)	0.36 (0.24 to 0.52)	0.31 (0.21 to 0.45)	0.25 (0.16 to 0.37)
Saint Vincent and the Grenadines	0.57 (0.38 to 0.83)	0.44 (0.29 to 0.62)	0.40 (0.26 to 0.57)	0.31 (0.19 to 0.45)
Suriname	4.90 (2.06 to 7.47)	1.30 (0.74 to 2.07)	3.63 (2.35 to 5.40)	2.67 (1.51 to 4.37)
Trinidad and Tobago	3.90 (2.73 to 5.26)	2.77 (1.93 to 3.73)	1.34 (0.90 to 1.88)	1.52 (0.95 to 2.30)
United States Virgin Islands	0.56 (0.32 to 0.95)	0.35 (0.22 to 0.54)	0.26 (0.14 to 0.43)	0.22 (0.12 to 0.38)
Central Latin America	0.79 (0.71 to 0.87)	1.25 (1.15 to 1.36)	1.49 (1.38 to 1.62)	1.31 (1.10 to 1.55)
Colombia	0.81 (0.62 to 1.02)	1.99 (1.60 to 2.42)	1.97 (1.59 to 2.40)	1.69 (1.09 to 2.40)
Costa Rica	0.74 (0.52 to 0.99)	0.77 (0.55 to 1.07)	0.99 (0.71 to 1.32)	0.83 (0.54 to 1.26)
El Salvador	4.18 (2.98 to 5.62)	3.57 (2.70 to 4.58)	3.08 (2.24 to 4.05)	1.85 (1.13 to 2.86)
Guatemala	0.54 (0.35 to 0.75)	0.83 (0.60 to 1.14)	1.11 (0.83 to 1.43)	1.00 (0.64 to 1.47)
Honduras	0.63 (0.35 to 1.07)	0.31 (0.15 to 0.53)	0.18 (0.09 to 0.32)	0.11 (0.06 to 0.19)
Mexico	0.46 (0.42 to 0.51)	0.91 (0.84 to 1.00)	1.49 (1.37 to 1.63)	1.44 (1.22 to 1.69)
Nicaragua	2.73 (1.82 to 3.85)	2.73 (2.02 to 3.57)	2.56 (1.95 to 3.31)	1.81 (1.14 to 2.68)
Panama	0.89 (0.58 to 1.28)	0.72 (0.50 to 1.02)	0.72 (0.51 to 1.02)	0.60 (0.39 to 0.89)
Venezuela (Bolivarian Republic of)	0.98 (0.74 to 1.27)	1.23 (0.96 to 1.56)	0.96 (0.76 to 1.22)	0.90 (0.58 to 1.34)
Tropical Latin America	0.69 (0.62 to 0.77)	0.74 (0.67 to 0.82)	0.87 (0.79 to 0.95)	0.88 (0.76 to 1.02)
Brazil	0.66 (0.59 to 0.74)	0.68 (0.62 to 0.74)	0.80 (0.72 to 0.89)	0.82 (0.71 to 0.95)
Paraguay	1.77 (1.16 to 2.56)	2.52 (1.83 to 3.41)	2.52 (1.82 to 3.29)	2.36 (1.47 to 3.63)
North Africa and Middle East	1.12 (0.89 to 1.38)	0.93 (0.78 to 1.12)	0.68 (0.57 to 0.84)	0.50 (0.39 to 0.64)
Afghanistan	0.28	0.24	0.19	0.15

	(0.14 to 0.52)	(0.12 to 0.41)	(0.10 to 0.31)	(0.08 to 0.25)
Algeria	1.94 (0.95 to 3.50)	1.24 (0.64 to 2.10)	0.83 (0.43 to 1.39)	0.63 (0.32 to 1.11)
Bahrain	0.25 (0.14 to 0.41)	0.36 (0.23 to 0.55)	0.26 (0.16 to 0.40)	0.20 (0.11 to 0.33)
Egypt	1.48 (0.74 to 2.55)	0.89 (0.46 to 1.62)	0.61 (0.30 to 1.10)	0.46 (0.23 to 0.88)
Iran (Islamic Republic of)	2.03 (1.66 to 2.43)	2.02 (1.73 to 2.33)	1.29 (1.11 to 1.51)	0.68 (0.56 to 0.82)
Iraq	0.37 (0.19 to 0.68)	0.37 (0.20 to 0.67)	0.25 (0.14 to 0.41)	0.15 (0.08 to 0.25)
Jordan	0.34 (0.20 to 0.55)	0.32 (0.19 to 0.53)	0.10 (0.06 to 0.15)	0.12 (0.07 to 0.20)
Kuwait	0.23 (0.15 to 0.33)	0.22 (0.15 to 0.31)	0.19 (0.13 to 0.27)	0.15 (0.10 to 0.23)
Lebanon	0.59 (0.31 to 1.04)	0.51 (0.26 to 0.88)	0.38 (0.19 to 0.66)	0.29 (0.14 to 0.51)
Libya	0.64 (0.33 to 1.08)	0.53 (0.28 to 0.90)	0.42 (0.22 to 0.73)	0.35 (0.19 to 0.60)
Morocco	1.20 (0.62 to 2.11)	1.15 (0.59 to 2.09)	0.91 (0.48 to 1.53)	0.66 (0.33 to 1.17)
Oman	0.20 (0.10 to 0.35)	0.15 (0.08 to 0.24)	0.13 (0.07 to 0.22)	0.10 (0.05 to 0.17)
Palestine	0.28 (0.15 to 0.48)	0.21 (0.13 to 0.34)	0.17 (0.10 to 0.26)	0.15 (0.08 to 0.24)
Qatar	0.54 (0.30 to 0.93)	0.56 (0.31 to 0.94)	0.36 (0.21 to 0.59)	0.28 (0.15 to 0.47)
Saudi Arabia	0.14 (0.07 to 0.26)	0.10 (0.06 to 0.16)	0.08 (0.05 to 0.12)	0.06 (0.03 to 0.11)
Sudan	1.30 (0.61 to 2.37)	1.21 (0.54 to 2.31)	1.07 (0.48 to 2.09)	0.86 (0.42 to 1.64)
Syrian Arab Republic	0.16 (0.09 to 0.26)	0.13 (0.08 to 0.20)	0.10 (0.06 to 0.17)	0.11 (0.06 to 0.18)
Tunisia	0.95 (0.50 to 1.64)	0.72 (0.38 to 1.22)	0.54 (0.27 to 0.95)	0.38 (0.19 to 0.68)
Turkey	0.40 (0.21 to 0.67)	0.31 (0.17 to 0.53)	0.89 (0.63 to 1.20)	0.65 (0.40 to 0.99)
United Arab Emirates	0.23 (0.11 to 0.41)	0.20 (0.11 to 0.35)	0.11 (0.06 to 0.20)	0.08 (0.04 to 0.15)
Yemen	1.34 (0.64 to 2.56)	1.28 (0.60 to 2.35)	1.06 (0.51 to 1.91)	0.93 (0.46 to 1.65)
South Asia	4.26 (3.21 to 5.39)	4.07 (3.19 to 4.97)	2.40 (1.87 to 2.91)	2.06 (1.56 to 2.58)
Bangladesh	2.41 (1.24 to 3.92)	1.92 (1.20 to 2.96)	1.37 (0.86 to 2.07)	0.65 (0.34 to 1.14)
Bhutan	0.46 (0.07 to 1.00)	0.53 (0.26 to 0.95)	0.39 (0.20 to 0.69)	0.35 (0.17 to 0.63)
India	5.16 (3.81 to 6.54)	4.95 (3.87 to 6.05)	2.87 (2.23 to 3.50)	2.54 (1.92 to 3.22)
Nepal	0.82 (0.41 to 1.49)	0.53 (0.27 to 0.97)	0.55 (0.28 to 1.03)	0.40 (0.20 to 0.74)
Pakistan	0.57 (0.33 to 0.92)	0.73 (0.44 to 1.12)	0.77 (0.48 to 1.20)	0.78 (0.46 to 1.30)
Southeast Asia, East Asia, and Oceania	2.73 (2.36 to 3.17)	2.36 (2.10 to 2.63)	0.95 (0.86 to 1.04)	0.55 (0.48 to 0.63)
East Asia	3.58 (3.04 to 4.27)	3.09 (2.74 to 3.48)	1.30 (1.17 to 1.45)	0.66 (0.56 to 0.77)
China	3.66 (3.09 to 4.38)	3.15 (2.79 to 3.54)	1.32 (1.19 to 1.47)	0.66 (0.56 to 0.77)
Democratic People's Republic of Korea	2.55 (1.22 to 4.56)	1.68 (0.85 to 2.95)	1.18 (0.62 to 2.01)	0.79 (0.41 to 1.35)
Taiwan, Province of China	0.42 (0.30 to 0.55)	0.49 (0.36 to 0.64)	0.40 (0.28 to 0.54)	0.31 (0.21 to 0.47)
Oceania	0.97 (0.71 to 1.32)	0.96 (0.72 to 1.28)	0.64 (0.47 to 0.87)	0.59 (0.43 to 0.79)
American Samoa	0.96 (0.54 to 1.56)	0.91 (0.55 to 1.42)	0.71 (0.42 to 1.14)	0.79 (0.43 to 1.34)
Cook Islands	3.01 (1.71 to 4.81)	2.10 (1.18 to 3.25)	1.26 (0.77 to 1.97)	0.97 (0.55 to 1.62)
Fiji	2.79 (1.47 to 4.59)	3.73 (2.26 to 5.75)	1.80 (1.10 to 2.62)	2.08 (1.15 to 3.45)
Guam	2.47 (1.51 to 3.78)	2.84 (1.76 to 4.14)	2.36 (1.51 to 3.46)	3.11 (1.86 to 4.74)
Kiribati	2.97 (1.71 to 4.81)	2.70 (1.55 to 4.36)	2.43 (1.34 to 4.04)	2.12 (1.07 to 3.64)
Marshall Islands	2.62 (1.48 to 4.29)	3.29 (1.90 to 5.40)	2.97 (1.56 to 5.09)	2.43 (1.27 to 4.02)
Micronesia (Federated States of)	3.02 (1.55 to 5.07)	2.77 (1.51 to 4.51)	2.34 (1.25 to 3.80)	1.95 (0.35 to 3.43)
Nauru	3.20	4.23	3.94	2.78

	(1.69 to 5.34)	(2.29 to 7.04)	(2.07 to 6.72)	(1.48 to 4.87)
Niue	1.90 (1.05 to 3.16)	2.16 (1.16 to 3.63)	1.88 (0.97 to 3.21)	1.63 (0.89 to 2.72)
Northern Mariana Islands	1.06 (0.56 to 1.93)	0.81 (0.46 to 1.31)	0.82 (0.47 to 1.35)	0.95 (0.53 to 1.55)
Palau	2.28 (1.22 to 3.86)	1.87 (1.00 to 3.20)	1.81 (0.99 to 3.09)	1.63 (0.87 to 2.71)
Papua New Guinea	0.40 (0.20 to 0.73)	0.37 (0.18 to 0.66)	0.35 (0.18 to 0.62)	0.32 (0.16 to 0.54)
Samoa	2.15 (1.16 to 3.63)	1.90 (1.01 to 3.14)	1.58 (0.81 to 2.69)	1.30 (0.66 to 2.35)
Solomon Islands	0.40 (0.20 to 0.72)	0.43 (0.21 to 0.76)	0.40 (0.20 to 0.72)	0.38 (0.19 to 0.65)
Tokelau	2.17 (1.11 to 3.64)	1.87 (0.87 to 3.36)	1.40 (0.68 to 2.47)	1.18 (0.57 to 2.10)
Tonga	0.93 (0.50 to 1.60)	1.25 (0.67 to 2.06)	0.88 (0.47 to 1.44)	0.75 (0.40 to 1.26)
Tuvalu	4.25 (2.27 to 7.19)	3.12 (1.71 to 5.05)	2.22 (1.17 to 3.85)	1.72 (0.89 to 3.00)
Vanuatu	1.97 (1.01 to 3.40)	2.61 (1.39 to 4.30)	2.40 (1.28 to 3.91)	2.14 (1.19 to 3.49)
Southeast Asia	1.11 (0.88 to 1.35)	0.73 (0.60 to 0.87)	0.49 (0.41 to 0.57)	0.42 (0.34 to 0.50)
Cambodia	1.22 (0.62 to 2.16)	0.93 (0.48 to 1.61)	0.54 (0.29 to 0.92)	0.38 (0.20 to 0.64)
Indonesia	0.77 (0.55 to 1.04)	0.60 (0.44 to 0.78)	0.45 (0.35 to 0.58)	0.37 (0.29 to 0.47)
Lao People's Democratic Republic	3.03 (1.52 to 5.41)	1.92 (1.02 to 3.33)	1.22 (0.61 to 2.11)	0.73 (0.38 to 1.29)
Malaysia	0.18 (0.10 to 0.30)	0.12 (0.07 to 0.18)	0.07 (0.04 to 0.10)	0.08 (0.04 to 0.14)
Maldives	0.60 (0.30 to 1.07)	0.22 (0.11 to 0.38)	0.12 (0.06 to 0.21)	0.10 (0.05 to 0.16)
Mauritius	4.13 (3.11 to 5.29)	2.62 (1.94 to 3.41)	1.34 (0.97 to 1.81)	1.68 (1.13 to 2.46)
Myanmar	1.01 (0.53 to 1.82)	0.85 (0.43 to 1.48)	0.51 (0.26 to 0.89)	0.36 (0.19 to 0.60)
Philippines	0.81 (0.68 to 0.96)	0.57 (0.48 to 0.66)	0.48 (0.41 to 0.55)	0.44 (0.35 to 0.53)
Seychelles	0.23 (0.13 to 0.40)	0.16 (0.09 to 0.25)	0.10 (0.06 to 0.15)	0.11 (0.06 to 0.18)
Sri Lanka	5.50 (3.75 to 7.59)	2.81 (2.13 to 3.63)	1.49 (1.03 to 2.01)	1.44 (0.83 to 2.28)
Thailand	1.83 (1.02 to 2.99)	1.06 (0.66 to 1.63)	0.56 (0.38 to 0.82)	0.45 (0.26 to 0.70)
Timor-Leste	1.77 (0.77 to 3.28)	1.21 (0.58 to 2.20)	0.69 (0.30 to 1.26)	0.59 (0.27 to 1.03)
Viet Nam	0.84 (0.44 to 1.47)	0.58 (0.31 to 0.96)	0.37 (0.22 to 0.61)	0.37 (0.20 to 0.63)
Sub-Saharan Africa	0.91 (0.70 to 1.16)	0.84 (0.65 to 1.05)	0.80 (0.64 to 0.96)	0.62 (0.49 to 0.78)
Central Sub-Saharan Africa	0.92 (0.56 to 1.47)	0.87 (0.50 to 1.39)	0.86 (0.52 to 1.31)	0.62 (0.39 to 0.95)
Angola	1.03 (0.47 to 1.89)	0.95 (0.40 to 1.76)	0.87 (0.44 to 1.54)	0.57 (0.28 to 0.99)
Central African Republic	1.10 (0.51 to 1.97)	0.95 (0.42 to 1.70)	1.09 (0.54 to 1.87)	0.92 (0.46 to 1.76)
Congo	0.67 (0.22 to 1.30)	0.76 (0.32 to 1.35)	0.75 (0.39 to 1.28)	0.48 (0.24 to 0.85)
Democratic Republic of the Congo	0.90 (0.45 to 1.64)	0.85 (0.39 to 1.56)	0.86 (0.44 to 1.49)	0.64 (0.33 to 1.09)
Equatorial Guinea	0.95 (0.46 to 1.80)	0.78 (0.32 to 1.42)	0.54 (0.22 to 1.03)	0.31 (0.12 to 0.63)
Gabon	0.64 (0.25 to 1.26)	0.52 (0.23 to 0.93)	0.46 (0.22 to 0.83)	0.31 (0.14 to 0.56)
Eastern Sub-Saharan Africa	1.27 (0.94 to 1.68)	1.09 (0.84 to 1.42)	1.00 (0.79 to 1.25)	0.73 (0.56 to 0.94)
Burundi	1.59 (0.80 to 2.78)	1.43 (0.71 to 2.51)	1.36 (0.70 to 2.39)	1.14 (0.60 to 2.00)
Comoros	1.05 (0.13 to 2.30)	1.13 (0.37 to 2.22)	0.70 (0.29 to 1.38)	0.91 (0.39 to 1.68)
Djibouti	0.92 (0.39 to 1.70)	1.07 (0.47 to 1.99)	0.97 (0.43 to 1.81)	0.84 (0.37 to 1.62)
Eritrea	0.97 (0.43 to 1.84)	0.93 (0.45 to 1.76)	0.99 (0.50 to 1.80)	0.89 (0.45 to 1.65)
Ethiopia	1.80 (1.09 to 2.79)	1.64 (1.06 to 2.36)	1.46 (0.98 to 2.08)	0.84 (0.54 to 1.31)
Kenya	0.65 (0.45 to 0.88)	0.45 (0.32 to 0.62)	0.53 (0.39 to 0.69)	0.44 (0.33 to 0.59)
Madagascar	2.12	1.17	1.11	0.90

	(1.09 to 3.72)	(0.64 to 1.98)	(0.55 to 1.91)	(0.43 to 1.60)
Malawi	1.33 (0.62 to 2.44)	1.18 (0.64 to 2.08)	0.76 (0.41 to 1.30)	0.59 (0.30 to 1.07)
Mozambique	1.14 (0.58 to 2.03)	0.81 (0.41 to 1.49)	0.95 (0.47 to 1.62)	0.60 (0.29 to 1.11)
Rwanda	1.71 (0.84 to 3.11)	1.48 (0.74 to 2.53)	0.91 (0.45 to 1.63)	0.74 (0.36 to 1.34)
Somalia	1.04 (0.50 to 1.90)	1.18 (0.52 to 2.19)	1.36 (0.69 to 2.37)	1.24 (0.66 to 2.19)
South Sudan	0.71 (0.34 to 1.30)	0.66 (0.27 to 1.27)	0.65 (0.31 to 1.21)	0.71 (0.34 to 1.38)
Uganda	0.48 (0.15 to 0.98)	0.80 (0.39 to 1.51)	0.58 (0.29 to 1.05)	0.54 (0.25 to 1.00)
United Republic of Tanzania	1.03 (0.51 to 1.82)	0.75 (0.41 to 1.29)	0.80 (0.44 to 1.41)	0.73 (0.36 to 1.32)
Zambia	1.30 (0.64 to 2.34)	1.11 (0.54 to 2.00)	0.94 (0.51 to 1.60)	0.59 (0.30 to 1.03)
Southern Sub-Saharan Africa	0.20 (0.15 to 0.27)	0.22 (0.18 to 0.27)	0.26 (0.20 to 0.34)	0.13 (0.08 to 0.21)
Botswana	0.20 (0.10 to 0.38)	0.20 (0.09 to 0.38)	0.24 (0.12 to 0.42)	0.23 (0.12 to 0.42)
Eswatini	0.23 (0.12 to 0.42)	0.20 (0.10 to 0.36)	0.24 (0.12 to 0.44)	0.21 (0.10 to 0.37)
Lesotho	0.27 (0.12 to 0.52)	0.21 (0.10 to 0.37)	0.28 (0.13 to 0.55)	0.26 (0.13 to 0.50)
Namibia	0.17 (0.05 to 0.35)	0.15 (0.06 to 0.27)	0.14 (0.07 to 0.26)	0.13 (0.05 to 0.23)
South Africa	0.16 (0.12 to 0.21)	0.26 (0.21 to 0.32)	0.25 (0.19 to 0.33)	0.02 (0.01 to 0.05)
Zimbabwe	0.32 (0.15 to 0.60)	0.11 (0.04 to 0.23)	0.31 (0.15 to 0.56)	0.39 (0.17 to 0.71)
Western Sub-Saharan Africa	0.72 (0.50 to 0.97)	0.72 (0.51 to 0.96)	0.68 (0.50 to 0.88)	0.59 (0.42 to 0.79)
Benin	0.84 (0.44 to 1.52)	0.82 (0.40 to 1.40)	0.79 (0.37 to 1.43)	0.73 (0.33 to 1.51)
Burkina Faso	0.87 (0.43 to 1.57)	0.92 (0.49 to 1.53)	0.87 (0.48 to 1.42)	0.92 (0.45 to 1.67)
Cabo Verde	0.31 (0.15 to 0.55)	0.25 (0.12 to 0.44)	0.17 (0.09 to 0.29)	0.17 (0.09 to 0.29)
Cameroon	0.76 (0.37 to 1.35)	0.90 (0.46 to 1.59)	0.91 (0.47 to 1.63)	0.74 (0.36 to 1.34)
Chad	0.83 (0.42 to 1.52)	0.99 (0.50 to 1.79)	1.05 (0.52 to 1.79)	1.02 (0.49 to 1.80)
Côte d'Ivoire	0.70 (0.33 to 1.24)	0.77 (0.35 to 1.38)	0.77 (0.36 to 1.35)	0.64 (0.30 to 1.23)
Gambia	0.69 (0.34 to 1.28)	0.74 (0.36 to 1.29)	0.71 (0.35 to 1.22)	0.53 (0.23 to 0.94)
Ghana	0.57 (0.30 to 1.01)	0.58 (0.33 to 0.95)	0.59 (0.34 to 0.99)	0.41 (0.21 to 0.75)
Guinea	1.10 (0.55 to 2.02)	0.99 (0.49 to 1.75)	1.10 (0.58 to 1.95)	1.03 (0.48 to 1.81)
Guinea-Bissau	1.14 (0.55 to 2.05)	1.14 (0.56 to 1.97)	1.04 (0.50 to 1.86)	0.70 (0.37 to 1.21)
Liberia	1.00 (0.51 to 1.84)	0.96 (0.48 to 1.70)	0.91 (0.47 to 1.61)	0.73 (0.34 to 1.31)
Mali	1.26 (0.65 to 2.29)	1.19 (0.60 to 2.07)	0.99 (0.51 to 1.73)	0.93 (0.42 to 1.74)
Mauritania	0.77 (0.38 to 1.35)	0.77 (0.40 to 1.35)	0.62 (0.31 to 1.08)	0.49 (0.22 to 0.96)
Niger	1.03 (0.50 to 1.87)	1.00 (0.50 to 1.78)	0.81 (0.40 to 1.46)	0.84 (0.39 to 1.61)
Nigeria	0.58 (0.30 to 0.95)	0.56 (0.28 to 0.93)	0.49 (0.26 to 0.79)	0.40 (0.20 to 0.69)
Sao Tome and Principe	0.24 (0.13 to 0.45)	0.33 (0.16 to 0.57)	0.21 (0.11 to 0.36)	0.15 (0.08 to 0.26)
Senegal	0.91 (0.45 to 1.57)	0.78 (0.38 to 1.39)	0.82 (0.41 to 1.47)	0.70 (0.34 to 1.27)
Sierra Leone	0.82 (0.41 to 1.41)	0.95 (0.47 to 1.70)	1.06 (0.52 to 1.86)	0.99 (0.45 to 1.80)
Togo	0.85 (0.45 to 1.48)	0.74 (0.35 to 1.28)	0.67 (0.34 to 1.16)	0.58 (0.28 to 1.12)
Africa	1.02 (0.80 to 1.29)	0.88 (0.71 to 1.08)	0.80 (0.65 to 0.95)	0.62 (0.50 to 0.76)
America	0.87 (0.82 to 0.92)	1.03 (0.98 to 1.10)	1.21 (1.14 to 1.28)	1.17 (1.06 to 1.30)
Asia	3.11 (2.65 to 3.64)	2.88 (2.50 to 3.30)	1.65 (1.39 to 1.91)	1.34 (1.08 to 1.62)
Europe	0.67 (0.63 to 0.71)	0.74 (0.70 to 0.78)	0.67 (0.62 to 0.73)	0.57 (0.51 to 0.64)

Supplementary Table 3. Deaths due to suicide among boys aged 10-14 years: country/territory-wise comparison of years of life lost (YLLs) together with 95% uncertainty intervals (UIs)–1990-2019.

	YLLs (95% UI)			
**Location**	**1990**	**2000**	**2010**	**2019**
Global	509,467 (348,255 to 595,540)	572,381 (415,344 to 656,521)	457,074 (351,814 to 530,696)	381,075 (299,773 to 465,128)
Central Europe, Eastern Europe, and Central Asia	49,329 (46,932 to 51,913)	62,951 (60,254 to 65,834)	30,715 (28,499 to 32,948)	27,359 (23,810 to 32,616)
Central Asia	10,754 (9406 to 12,122)	16,064 (14,092 to 18,207)	13,130 (11,167 to 15,254)	12,524 (9872 to 16,135)
Armenia	50 (33 to 72)	62 (41 to 93)	100 (68 to 146)	64 (38 to 102)
Azerbaijan	163 (92 to 387)	267 (160 to 541)	287 (161 to 468)	213 (104 to 386)
Georgia	132 (92 to 188)	113 (78 to 160)	73 (47 to 104)	59 (38 to 87)
Kazakhstan	4550 (3669 to 5580)	5936 (4832 to 7181)	3638 (2986 to 4327)	2995 (2092 to 4052)
Kyrgyzstan	1177 (901 to 1474)	1335 (1027 to 1653)	842 (664 to 1053)	1069 (759 to 1435)
Mongolia	850 (286 to 1445)	1031 (355 to 1678)	1054 (339 to 1669)	668 (227 to 1121)
Tajikistan	339 (224 to 509)	450 (279 to 725)	423 (249 to 677)	446 (245 to 747)
Turkmenistan	385 (275 to 531)	783 (585 to 1019)	371 (263 to 503)	287 (170 to 438)
Uzbekistan	3109 (2443 to 3868)	6086 (4804 to 7580)	6342 (4892 to 8066)	6724 (4431 to 9428)
Central Europe	10,228 (9297 to 11,257)	8043 (7383 to 8814)	3796 (3464 to 4213)	2876 (2376 to 3459)
Albania	141 (78 to 247)	192 (106 to 287)	165 (65 to 289)	97 (37 to 174)
Bosnia and Herzegovina	429 (182 to 627)	221 (100 to 366)	100 (51 to 164)	100 (50 to 173)
Bulgaria	1164 (911 to 1446)	618 (474 to 792)	243 (169 to 337)	193 (119 to 303)
Croatia	376 (272 to 497)	273 (195 to 370)	138 (97 to 189)	94 (57 to 145)
Czechia	903 (679 to 1157)	486 (369 to 629)	244 (173 to 329)	178 (109 to 270)
Hungary	879 (681 to 1127)	494 (357 to 649)	220 (157 to 297)	174 (109 to 266)
Montenegro	56 (32 to 91)	69 (40 to 105)	29 (17 to 45)	27 (13 to 47)
North Macedonia	102 (56 to 175)	87 (50 to 145)	42 (24 to 70)	46 (24 to 79)
Poland	3456 (3128 to 3799)	2810 (2565 to 3064)	1393 (1254 to 1541)	1000 (800 to 1243)
Romania	1416 (1077 to 1823)	1940 (1514 to 2417)	887 (677 to 1130)	668 (442 to 951)
Serbia	810 (389 to 1422)	456 (269 to 791)	152 (86 to 325)	128 (61 to 250)
Slovakia	317 (181 to 523)	263 (166 to 412)	131 (79 to 211)	96 (46 to 167)
Slovenia	177 (124 to 236)	135 (99 to 179)	50 (35 to 68)	75 (46 to 111)
Eastern Europe	28,347 (26,854 to 29,899)	38,844 (37,119 to 40,678)	13,789 (13,097 to 14,541)	11,959 (10,231 to 14,137)
Belarus	1013 (767 to 1303)	1434 (1113 to 1819)	560 (408 to 745)	337 (192 to 536)
Estonia	279 (202 to 372)	160 (112 to 221)	49 (34 to 68)	43 (27 to 66)
Latvia	274 (199 to 363)	193 (135 to 259)	80 (54 to 111)	47 (27 to 75)
Lithuania	351 (256 to 461)	414 (311 to 539)	179 (127 to 244)	125 (82 to 181)
Republic of Moldova	407 (287 to 546)	453 (321 to 619)	206 (144 to 288)	149 (93 to 222)
Russian Federation	21,245 (20,245 to 22,329)	30,192 (29,124 to 31,459)	10,733 (10,240 to 11,296)	8076 (6852 to 9756)
Ukraine	4778 (3874 to 5922)	5998 (4871 to 7258)	1982 (1581 to 2426)	3184 (2150 to 4600)
High-income	36,994 (35,086 to 40,684)	36,815 (35,281 to 38,341)	32,380 (30,420 to 33,887)	32,004 (29,622 to 34,589)
Australasia	779 (615 to 959)	867 (713 to 1055)	667 (546 to 816)	681 (507 to 906)
Australia	541	575	452	514

	(403 to 709)	(436 to 748)	(339 to 594)	(347 to 735)
New Zealand	238 (180 to 304)	292 (218 to 368)	215 (167 to 266)	168 (119 to 225)
High-income Asia Pacific	6032 (5018 to 9443)	5249 (4748 to 5957)	5853 (4556 to 6723)	3889 (3271 to 4503)
Brunei Darussalam	7 (3 to 14)	6 (3 to 11)	5 (3 to 8)	5 (3 to 10)
Japan	3296 (3077 to 3568)	3491 (3272 to 3727)	3562 (3318 to 3808)	2811 (2560 to 3069)
Republic of Korea	2619 (1678 to 5976)	1639 (1186 to 2278)	2202 (960 to 2981)	1021 (451 to 1585)
Singapore	110 (74 to 152)	114 (79 to 157)	84 (57 to 121)	52 (33 to 78)
High-income North America	17,894 (16,996 to 18,846)	19,287 (18,378 to 20,245)	16,064 (15,079 to 16,907)	19,411 (17,873 to 21,003)
Canada	1923 (1545 to 2336)	1887 (1549 to 2278)	1243 (992 to 1517)	1452 (997 to 1997)
Greenland	29 (15 to 41)	27 (16 to 36)	20 (11 to 27)	13 (7 to 20)
United States of America	15,942 (15,122 to 16,763)	17,372 (16,541 to 18,204)	14,801 (13,931 to 15,613)	17,946 (16,633 to 19,431)
Southern Latin America	2448 (2058 to 2911)	2984 (2494 to 3536)	3782 (3112 to 4508)	2932 (2120 to 3918)
Argentina	1491 (1154 to 1907)	2060 (1641 to 2574)	2829 (2228 to 3491)	2247 (1496 to 3178)
Chile	783 (584 to 1034)	755 (580 to 995)	773 (591 to 996)	545 (351 to 779)
Uruguay	173 (116 to 242)	169 (116 to 236)	180 (122 to 251)	140 (87 to 208)
Western Europe	9842 (9007 to 10,729)	8428 (7715 to 9151)	6015 (5440 to 6624)	5090 (4414 to 5884)
Andorra	1 (0 to 2)	1 (0 to 1)	1 (0 to 1)	0 (0 to 1)
Austria	280 (203 to 375)	269 (194 to 360)	156 (114 to 207)	129 (83 to 182)
Belgium	381 (279 to 501)	377 (278 to 489)	321 (231 to 429)	249 (157 to 356)
Cyprus	13 (6 to 23)	17 (8 to 33)	4 (2 to 6)	7 (4 to 13)
Denmark	187 (133 to 254)	120 (84 to 168)	49 (32 to 69)	50 (30 to 74)
Finland	287 (211 to 377)	143 (103 to 197)	97 (66 to 133)	101 (65 to 146)
France	1691 (1322 to 2117)	1904 (1509 to 2315)	1543 (1221 to 1929)	1208 (806 to 1776)
Germany	2317 (1820 to 2870)	2320 (1862 to 2827)	1229 (947 to 1556)	916 (607 to 1294)
Greece	133 (91 to 187)	74 (48 to 111)	57 (37 to 84)	55 (33 to 84)
Iceland	13 (8 to 18)	5 (3 to 7)	7 (4 to 10)	6 (3 to 9)
Ireland	141 (98 to 200)	159 (109 to 209)	125 (87 to 171)	104 (67 to 158)
Israel	140 (98 to 197)	198 (138 to 275)	184 (128 to 256)	170 (107 to 249)
Italy	866 (778 to 962)	598 (532 to 671)	445 (398 to 496)	422 (363 to 498)
Luxembourg	8 (5 to 12)	12 (8 to 17)	11 (7 to 17)	7 (4 to 11)
Malta	3 (2 to 5)	4 (2 to 5)	6 (4 to 8)	3 (2 to 5)
Monaco	0 (0 to 1)	0 (0 to 1)	0 (0 to 1)	0 (0 to 1)
Netherlands	362 (259 to 478)	377 (279 to 503)	343 (251 to 454)	239 (153 to 345)
Norway	380 (346 to 416)	247 (215 to 286)	200 (179 to 221)	152 (132 to 175)
Portugal	328 (224 to 459)	124 (80 to 181)	98 (65 to 142)	57 (34 to 88)
San Marino	1 (0 to 1)	1 (0 to 1)	1 (0 to 1)	1 (0 to 1)
Spain	1391 (1063 to 1787)	548 (403 to 725)	435 (313 to 585)	422 (276 to 606)
Sweden	176 (129 to 235)	189 (140 to 248)	148 (109 to 199)	135 (94 to 187)
Switzerland	227 (162 to 308)	248 (177 to 339)	144 (102 to 199)	131 (84 to 191)
United Kingdom	508 (466 to 558)	488 (452 to 527)	408 (375 to 440)	522 (473 to 573)
Latin America and Caribbean	16,047	22,589	28,556	24,231

	(14,318 to 18,271)	(20,648 to 24,370)	(24,768 to 31,132)	(20,246 to 28,634)
Andean Latin America	1825 (1285 to 3165)	3012 (1906 to 3899)	4824 (1826 to 6478)	3904 (1408 to 5905)
Bolivia (Plurinational State of)	479 (237 to 816)	712 (356 to 1198)	707 (344 to 1254)	602 (298 to 1065)
Ecuador	667 (450 to 1033)	1440 (614 to 2055)	3272 (827 to 4743)	2685 (606 to 4352)
Peru	679 (373 to 1453)	861 (564 to 1344)	846 (544 to 1228)	617 (322 to 1075)
Caribbean	1478 (976 to 1990)	1510 (1079 to 2004)	1380 (924 to 1948)	1375 (857 to 2062)
Antigua and Barbuda	0 (0 to 1)	1 (1 to 1)	1 (0 to 1)	0 (0 to 1)
Bahamas	2 (1 to 3)	3 (2 to 4)	2 (2 to 4)	2 (1 to 4)
Barbados	4 (2 to 6)	2 (1 to 4)	3 (2 to 5)	2 (1 to 3)
Belize	6 (4 to 8)	9 (6 to 14)	9 (6 to 14)	9 (5 to 14)
Bermuda	0 (0 to 0)	0 (0 to 0)	0 (0 to 0)	0 (0 to 0)
Cuba	271 (190 to 376)	300 (210 to 410)	185 (127 to 257)	145 (84 to 229)
Dominica	1 (1 to 2)	1 (1 to 2)	2 (1 to 2)	1 (1 to 2)
Dominican Republic	194 (113 to 336)	228 (142 to 361)	252 (140 to 414)	199 (101 to 357)
Grenada	1 (1 to 2)	2 (1 to 3)	2 (1 to 2)	1 (1 to 1)
Guyana	103 (70 to 145)	99 (69 to 138)	119 (84 to 163)	71 (44 to 106)
Haiti	571 (158 to 991)	575 (198 to 1027)	596 (216 to 1118)	754 (281 to 1369)
Jamaica	25 (16 to 37)	28 (18 to 42)	38 (24 to 57)	31 (18 to 51)
Puerto Rico	106 (71 to 155)	71 (48 to 103)	41 (27 to 61)	26 (16 to 40)
Saint Kitts and Nevis	1 (0 to 1)	1 (0 to 1)	1 (1 to 2)	0 (0 to 1)
Saint Lucia	4 (2 to 6)	3 (2 to 5)	3 (2 to 4)	2 (1 to 4)
Saint Vincent and the Grenadines	2 (1 to 4)	4 (2 to 6)	2 (1 to 3)	2 (1 to 3)
Suriname	28 (16 to 44)	39 (23 to 60)	29 (18 to 45)	42 (22 to 71)
Trinidad and Tobago	105 (70 to 149)	88 (59 to 125)	46 (31 to 68)	39 (22 to 66)
United States Virgin Islands	4 (3 to 8)	5 (3 to 7)	1 (1 to 2)	1 (0 to 1)
Central Latin America	7794 (7096 to 8598)	12,956 (11,863 to 14,053)	15,879 (14,471 to 17,235)	12,687 (10,588 to 15,203)
Colombia	1427 (1106 to 1811)	2748 (2140 to 3393)	3846 (3040 to 4726)	2325 (1372 to 3663)
Costa Rica	161 (113 to 226)	199 (140 to 271)	209 (144 to 291)	182 (114 to 286)
El Salvador	410 (240 to 717)	289 (174 to 479)	280 (180 to 439)	201 (91 to 367)
Guatemala	267 (168 to 386)	445 (301 to 641)	474 (327 to 653)	393 (230 to 644)
Honduras	226 (111 to 515)	175 (74 to 473)	139 (57 to 397)	113 (45 to 331)
Mexico	3822 (3472 to 4200)	6825 (6258 to 7424)	9223 (8416 to 10,081)	7977 (6827 to 9343)
Nicaragua	316 (172 to 500)	441 (261 to 638)	447 (244 to 629)	330 (187 to 551)
Panama	95 (59 to 140)	168 (112 to 240)	172 (117 to 246)	138 (83 to 223)
Venezuela (Bolivarian Republic of)	1069 (803 to 1387)	1667 (1321 to 2113)	1090 (804 to 1426)	1029 (570 to 1654)
Tropical Latin America	4950 (4399 to 5465)	5110 (4518 to 5645)	6472 (5696 to 7163)	6265 (5362 to 7242)
Brazil	4792 (4240 to 5324)	4835 (4239 to 5357)	6085 (5351 to 6749)	5913 (5052 to 6794)
Paraguay	158 (90 to 239)	275 (123 to 413)	387 (124 to 582)	353 (112 to 606)
North Africa and Middle East	20,999 (9381 to 28,771)	23,407 (10,155 to 30,763)	14,623 (7623 to 18,950)	11,042 (6231 to 14,779)
Afghanistan	776 (174 to 1487)	1261 (286 to 2286)	1625 (416 to 2971)	1533 (422 to 2948)
Algeria	2120	1670	827	687

	(825 to 3825)	(766 to 3022)	(417 to 1469)	(355 to 1219)
Bahrain	8 (4 to 16)	9 (5 to 15)	12 (7 to 19)	9 (5 to 17)
Egypt	2522 (950 to 4571)	2213 (965 to 4100)	1863 (830 to 3502)	1738 (811 to 3263)
Iran (Islamic Republic of)	4910 (1394 to 6674)	5853 (1595 to 7546)	2552 (903 to 3223)	1129 (569 to 1448)
Iraq	613 (253 to 1300)	812 (356 to 1807)	708 (370 to 1634)	459 (236 to 1013)
Jordan	81 (38 to 150)	90 (44 to 168)	54 (29 to 103)	106 (53 to 196)
Kuwait	27 (16 to 46)	21 (14 to 32)	22 (15 to 32)	27 (16 to 45)
Lebanon	86 (38 to 152)	75 (34 to 146)	49 (23 to 97)	48 (22 to 98)
Libya	131 (66 to 229)	101 (50 to 184)	109 (54 to 195)	63 (32 to 117)
Morocco	1199 (432 to 2205)	1930 (853 to 3459)	1072 (531 to 1888)	667 (335 to 1204)
Oman	87 (35 to 162)	85 (32 to 146)	58 (21 to 104)	57 (22 to 110)
Palestine	46 (21 to 81)	54 (28 to 87)	53 (29 to 83)	64 (31 to 113)
Qatar	9 (4 to 17)	10 (5 to 18)	13 (8 to 21)	14 (7 to 27)
Saudi Arabia	212 (100 to 379)	212 (122 to 346)	201 (119 to 308)	130 (69 to 235)
Sudan	1197 (330 to 2538)	1550 (498 to 3087)	1625 (583 to 3392)	1581 (688 to 3012)
Syrian Arab Republic	271 (122 to 526)	256 (146 to 496)	271 (146 to 473)	185 (88 to 344)
Tunisia	345 (167 to 625)	298 (142 to 559)	174 (85 to 326)	134 (65 to 253)
Turkey	5449 (1728 to 9912)	5749 (1675 to 9891)	2319 (932 to 3408)	1220 (599 to 1947)
United Arab Emirates	40 (20 to 74)	64 (33 to 113)	49 (27 to 81)	42 (22 to 87)
Yemen	854 (253 to 1720)	1077 (438 to 2076)	952 (415 to 1882)	1134 (519 to 2097)
South Asia	158,947 (94,810 to 212,930)	209,679 (137,294 to 266,382)	194,169 (139,690 to 250,489)	146,599 (105,828 to 199,363)
Bangladesh	34,249 (10,751 to 60,613)	38,073 (17,068 to 59,841)	36,459 (20,168 to 55,132)	21,205 (11,904 to 34,853)
Bhutan	22 (3 to 60)	26 (10 to 71)	23 (10 to 64)	19 (7 to 54)
India	109,747 (67,652 to 146,047)	149,963 (100,029 to 194,331)	129,357 (92,016 to 164,660)	92,165 (62,334 to 131,934)
Nepal	2901 (1090 to 6814)	2332 (1007 to 5707)	2303 (1048 to 5758)	1986 (912 to 4711)
Pakistan	12,028 (5995 to 21,437)	19,286 (9722 to 34,097)	26,026 (12,476 to 46,761)	31,225 (15,788 to 55,929)
Southeast Asia, East Asia, and Oceania	165,286 (88,602 to 200,687)	141,161 (89,083 to 165,240)	67,563 (51,344 to 79,839)	42,411 (31,867 to 52,803)
East Asia	134,315 (73,220 to 163,712)	115,810 (74,541 to 137,480)	51,007 (40,324 to 61,566)	28,719 (21,750 to 37,093)
China	132,141 (71,650 to 161,432)	113,852 (72,526 to 134,912)	49,411 (38,937 to 59,491)	27,724 (20,743 to 35,660)
Democratic People's Republic of Korea	1766 (848 to 3157)	1663 (802 to 2901)	1279 (654 to 2157)	799 (408 to 1396)
Taiwan, Province of China	407 (291 to 562)	295 (207 to 407)	317 (225 to 437)	196 (116 to 314)
Oceania	600 (397 to 978)	733 (496 to 1,245)	768 (515 to 1386)	794 (532 to 1448)
American Samoa	3 (2 to 5)	4 (2 to 5)	3 (2 to 6)	3 (1 to 5)
Cook Islands	4 (2 to 6)	2 (1 to 4)	1 (1 to 2)	1 (0 to 1)
Fiji	95 (51 to 155)	125 (71 to 193)	90 (54 to 135)	99 (51 to 169)
Guam	25 (15 to 37)	38 (24 to 54)	23 (15 to 33)	18 (11 to 29)
Kiribati	29 (16 to 45)	37 (22 to 60)	35 (19 to 57)	33 (18 to 55)
Marshall Islands	9 (5 to 15)	14 (7 to 24)	11 (5 to 18)	9 (5 to 15)
Micronesia (Federated States of)	31 (16 to 52)	28 (14 to 47)	21 (12 to 34)	15 (4 to 25)
Nauru	2 (1 to 4)	3 (1 to 6)	3 (1 to 5)	2 (1 to 4)
Niue	0	0	0	0

	(0 to 1)	(0 to 1)	(0 to 0)	(0 to 0)
Northern Mariana Islands	3<br>(2 to 6)	4<br>(2 to 6)	5<br>(3 to 8)	4<br>(2 to 6)
Palau	2<br>(1 to 3)	1<br>(0 to 1)	1<br>(1 to 2)	1<br>(0 to 1)
Papua New Guinea	169<br>(68 to 502)	209<br>(82 to 644)	281<br>(107 to 836)	305<br>(125 to 914)
Samoa	41<br>(21 to 66)	31<br>(16 to 51)	28<br>(14 to 46)	26<br>(14 to 43)
Solomon Islands	116<br>(50 to 201)	145<br>(61 to 246)	168<br>(73 to 293)	180<br>(81 to 308)
Tokelau	0<br>(0 to 0)	0<br>(0 to 0)	0<br>(0 to 0)	0<br>(0 to 0)
Tonga	5<br>(2 to 8)	6<br>(3 to 10)	5<br>(3 to 9)	4<br>(2 to 7)
Tuvalu	2<br>(1 to 4)	2<br>(1 to 3)	2<br>(1 to 3)	1<br>(1 to 2)
Vanuatu	31<br>(15 to 53)	42<br>(20 to 72)	51<br>(24 to 87)	54<br>(27 to 91)
Southeast Asia	30,371<br>(14,566 to 40,623)	24,618<br>(12,699 to 31,305)	15,788<br>(9830 to 19,656)	12,899<br>(8560 to 16,084)
Cambodia	999<br>(339 to 1879)	1111<br>(389 to 1958)	725<br>(275 to 1297)	546<br>(219 to 1010)
Indonesia	7760<br>(3830 to 10,702)	6261<br>(3274 to 8285)	4875<br>(2690 to 6266)	3879<br>(2348 to 5184)
Lao People's Democratic Republic	502<br>(142 to 922)	480<br>(159 to 870)	373<br>(160 to 648)	245<br>(117 to 444)
Malaysia	279<br>(125 to 686)	276<br>(143 to 716)	238<br>(125 to 662)	236<br>(104 to 616)
Maldives	9<br>(3 to 18)	7<br>(3 to 13)	4<br>(2 to 7)	3<br>(2 to 6)
Mauritius	63<br>(42 to 91)	47<br>(30 to 69)	28<br>(18 to 42)	16<br>(9 to 26)
Myanmar	4902<br>(1251 to 10,239)	4330<br>(1276 to 8426)	2705<br>(929 to 4960)	1906<br>(745 to 3273)
Philippines	2908<br>(1690 to 3681)	2814<br>(1725 to 3511)	2790<br>(1582 to 3324)	2862<br>(1527 to 3688)
Seychelles	1<br>(0 to 2)	0<br>(0 to 1)	1<br>(1 to 2)	1<br>(1 to 2)
Sri Lanka	3200<br>(1632 to 5039)	1624<br>(1103 to 2253)	800<br>(504 to 1349)	743<br>(363 to 1379)
Thailand	6726<br>(2571 to 11,431)	4759<br>(2002 to 7605)	1607<br>(1014 to 2550)	884<br>(489 to 1628)
Timor-Leste	37<br>(13 to 72)	38<br>(17 to 68)	37<br>(16 to 64)	44<br>(20 to 78)
Viet Nam	2944<br>(1291 to 5215)	2839<br>(1483 to 4831)	1584<br>(921 to 2572)	1517<br>(786 to 2794)
Sub-Saharan Africa	61,864<br>(39,624 to 78,285)	75,778<br>(51,672 to 96,044)	89,067<br>(63,364 to 109,843)	97,429<br>(68,821 to 129,573)
Central Sub-Saharan Africa	8545<br>(5196 to 12,894)	10,976<br>(6593 to 17,478)	13,210<br>(8107 to 21,195)	14,226<br>(8241 to 26,134)
Angola	2025<br>(856 to 3667)	2433<br>(975 to 4351)	3067<br>(1657 to 5166)	3440<br>(1803 to 6043)
Central African Republic	605<br>(297 to 1032)	705<br>(333 to 1243)	930<br>(473 to 1622)	968<br>(483 to 1710)
Congo	328<br>(112 to 625)	376<br>(165 to 659)	362<br>(178 to 664)	384<br>(182 to 728)
Democratic Republic of the Congo	5371<br>(2661 to 9121)	7234<br>(3461 to 13,223)	8604<br>(4312 to 15,079)	9210<br>(4289 to 18,996)
Equatorial Guinea	87<br>(42 to 160)	103<br>(48 to 190)	105<br>(47 to 205)	105<br>(44 to 226)
Gabon	128<br>(53 to 235)	124<br>(58 to 229)	142<br>(74 to 251)	119<br>(59 to 227)
Eastern Sub-Saharan Africa	34,214<br>(19,280 to 45,171)	38,310<br>(23,124 to 48,686)	41,174<br>(27,719 to 50,788)	42,439<br>(28,140 to 57,961)
Burundi	1232<br>(626 to 2192)	1350<br>(661 to 2377)	1424<br>(720 to 2481)	1648<br>(824 to 2993)
Comoros	58<br>(11 to 117)	56<br>(19 to 107)	38<br>(15 to 73)	49<br>(20 to 101)
Djibouti	52<br>(21 to 98)	84<br>(33 to 165)	90<br>(37 to 178)	104<br>(43 to 206)
Eritrea	493<br>(211 to 950)	666<br>(279 to 1285)	826<br>(363 to 1514)	788<br>(324 to 1513)
Ethiopia	12,267<br>(5433 to 18,836)	12,386<br>(5665 to 18,311)	9928<br>(5600 to 13,755)	9125<br>(5356 to 13,557)
Kenya	1936<br>(1144 to 2648)	2502<br>(1521 to 3343)	3960<br>(2478 to 5172)	3739<br>(2274 to 5820)
Madagascar	3137<br>(1062 to 5755)	2155<br>(978 to 3703)	2417<br>(1258 to 4032)	2434<br>(1252 to 4321)
Malawi	1887	2178	2404	2587

	(866 to 3235)	(1187 to 3637)	(1352 to 3884)	(1305 to 4625)
Mozambique	2147 (960 to 3747)	2214 (1123 to 3758)	3667 (1963 to 6253)	3797 (1818 to 7147)
Rwanda	1609 (783 to 2909)	1880 (935 to 3225)	1165 (597 to 2145)	1215 (614 to 2190)
Somalia	970 (371 to 1847)	1686 (647 to 3309)	2717 (1097 to 5175)	3274 (1349 to 6225)
South Sudan	479 (217 to 863)	715 (311 to 1339)	802 (387 to 1511)	946 (456 to 1896)
Uganda	1816 (604 to 3734)	4045 (1715 to 7296)	4019 (1875 to 7236)	4529 (2057 to 8007)
United Republic of Tanzania	4742 (2343 to 8539)	4471 (2573 to 7055)	5240 (2871 to 8768)	6007 (3172 to 10,158)
Zambia	1364 (643 to 2469)	1890 (983 to 3167)	2444 (1372 to 4132)	2162 (1131 to 3842)
Southern Sub-Saharan Africa	1100 (800 to 1555)	1595 (1255 to 2200)	1396 (1017 to 1920)	944 (556 to 1521)
Botswana	43 (23 to 78)	66 (33 to 113)	51 (25 to 92)	57 (28 to 100)
Eswatini	33 (16 to 58)	45 (23 to 78)	46 (23 to 82)	41 (22 to 72)
Lesotho	82 (40 to 147)	89 (43 to 163)	91 (46 to 170)	86 (42 to 150)
Namibia	36 (13 to 65)	49 (23 to 86)	55 (28 to 94)	48 (23 to 86)
South Africa	563 (413 to 896)	1,091 (850 to 1,644)	698 (531 to 971)	134 (60 to 315)
Zimbabwe	343 (151 to 653)	256 (101 to 502)	454 (214 to 818)	579 (262 to 1103)
Western Sub-Saharan Africa	18,006 (10,846 to 24,710)	24,897 (15,971 to 34,050)	33,288 (23,366 to 44,036)	39,820 (27,514 to 53,727)
Benin	616 (306 to 1052)	914 (446 to 1542)	1181 (602 to 1985)	1383 (659 to 2633)
Burkina Faso	1333 (577 to 2460)	1668 (770 to 2892)	2247 (1071 to 4056)	2956 (1211 to 5750)
Cabo Verde	32 (16 to 54)	52 (28 to 90)	36 (20 to 59)	32 (17 to 54)
Cameroon	1045 (484 to 1807)	2130 (1030 to 3718)	2998 (1574 to 5063)	3408 (1726 to 6176)
Chad	651 (284 to 1170)	1166 (512 to 2111)	1785 (808 to 3260)	2471 (1184 to 4529)
Côte d'Ivoire	2037 (1014 to 3605)	3080 (1620 to 5391)	3347 (1786 to 5634)	3412 (1694 to 6230)
Gambia	77 (30 to 151)	115 (48 to 221)	141 (57 to 275)	160 (68 to 307)
Ghana	1290 (667 to 2219)	1275 (703 to 2324)	2491 (1502 to 3958)	2562 (1322 to 4563)
Guinea	645 (264 to 1190)	875 (393 to 1610)	1357 (580 to 2408)	1664 (746 to 3032)
Guinea-Bissau	196 (81 to 372)	249 (111 to 453)	256 (120 to 449)	243 (116 to 437)
Liberia	240 (115 to 426)	277 (133 to 482)	358 (175 to 624)	395 (194 to 696)
Mali	919 (377 to 1781)	1061 (437 to 1915)	1563 (671 to 2923)	2246 (956 to 4211)
Mauritania	171 (74 to 316)	198 (89 to 365)	205 (92 to 377)	223 (91 to 498)
Niger	1016 (355 to 1927)	1216 (495 to 2260)	1543 (674 to 2894)	2494 (1077 to 4899)
Nigeria	5835 (2446 to 9841)	8312 (3821 to 13,969)	10,830 (5896 to 17,795)	12,814 (7632 to 21,051)
Sao Tome and Principe	5 (3 to 10)	3 (2 to 6)	3 (2 to 6)	3 (2 to 6)
Senegal	1089 (520 to 1897)	1189 (577 to 2053)	1461 (803 to 2477)	1687 (836 to 3002)
Sierra Leone	374 (145 to 713)	514 (212 to 981)	734 (314 to 1327)	864 (384 to 1621)
Togo	431 (218 to 751)	603 (288 to 1042)	749 (382 to 1274)	802 (386 to 1440)
Africa	69,417 (43,715 to 87,757)	83,555 (55,926 to 104,832)	94,733 (66,826 to 116,725)	102,282 (72,222 to 133,987)
America	36,200 (34,148 to 38,579)	44,706 (42,338 to 46,884)	48,292 (44,304 to 51,149)	46,487 (41,930 to 51,777)
Asia	349,445 (210,902 to 420,771)	382,489 (258,308 to 452,040)	287,603 (213,867 to 350,361)	210,783 (158,935 to 271,131)
Europe	54,091 (49,820 to 59,116)	61,328 (56,924 to 65,941)	26,210 (24,509 to 27,877)	21,319 (19,068 to 24,437)

Supplementary Table 4. Deaths due to intentional self-harm among girls aged 10-14 years: country/territory-wise comparison of years of life lost (YLLs) together with 95% uncertainty intervals (UIs)–1990-2019.

Location	YLLs (95% UI) 1990	2000	2010	2019
Global	446,123 (386,005 to 514,141)	491,841 (435,122 to 553,902)	301,111 (261,735 to 339,306)	255,121 (217,770 to 295,046)
Central Europe, Eastern Europe, and Central Asia	13,070 (12,355 to 13,870)	16,382 (15,496 to 17,336)	12,164 (11,255 to 13,099)	11,667 (10,122 to 13,359)
Central Asia	3200 (2764 to 3692)	4555 (4012 to 5203)	5290 (4456 to 6121)	5406 (4229 to 6896)
Armenia	24 (16 to 35)	18 (12 to 27)	30 (21 to 41)	22 (14 to 32)
Azerbaijan	56 (35 to 86)	60 (37 to 95)	68 (36 to 116)	53 (28 to 92)
Georgia	66 (42 to 98)	44 (28 to 68)	36 (22 to 54)	19 (11 to 31)
Kazakhstan	942 (728 to 1193)	1539 (1227 to 1919)	1299 (1028 to 1609)	1184 (837 to 1600)
Kyrgyzstan	362 (260 to 468)	318 (230 to 422)	271 (197 to 357)	288 (193 to 396)
Mongolia	452 (260 to 727)	382 (205 to 600)	329 (197 to 528)	269 (155 to 446)
Tajikistan	168 (109 to 248)	209 (131 to 313)	188 (101 to 308)	234 (121 to 402)
Turkmenistan	169 (119 to 230)	257 (187 to 345)	170 (120 to 232)	135 (88 to 200)
Uzbekistan	961 (744 to 1216)	1729 (1349 to 2198)	2898 (2224 to 3664)	3202 (2175 to 4424)
Central Europe	2922 (2641 to 3215)	2039 (1865 to 2230)	1285 (1165 to 1417)	886 (747 to 1041)
Albania	105 (66 to 155)	113 (74 to 162)	130 (80 to 201)	59 (33 to 95)
Bosnia and Herzegovina	151 (101 to 220)	70 (41 to 112)	42 (26 to 63)	43 (25 to 66)
Bulgaria	442 (326 to 582)	264 (191 to 351)	112 (79 to 156)	76 (48 to 115)
Croatia	127 (90 to 173)	78 (55 to 108)	56 (40 to 78)	35 (22 to 50)
Czechia	179 (126 to 244)	127 (90 to 173)	55 (38 to 76)	48 (31 to 72)
Hungary	284 (205 to 383)	128 (88 to 178)	62 (43 to 87)	48 (31 to 73)
Montenegro	23 (14 to 36)	26 (16 to 40)	15 (9 to 22)	7 (4 to 11)
North Macedonia	99 (63 to 143)	65 (43 to 94)	32 (20 to 46)	28 (16 to 43)
Poland	700 (624 to 779)	572 (514 to 635)	444 (393 to 491)	300 (246 to 365)
Romania	368 (268 to 501)	329 (239 to 441)	214 (152 to 291)	157 (103 to 226)
Serbia	277 (157 to 440)	157 (103 to 230)	59 (38 to 88)	35 (20 to 57)
Slovakia	109 (65 to 171)	69 (44 to 100)	47 (31 to 69)	33 (19 to 52)
Slovenia	57 (38 to 79)	40 (29 to 55)	17 (12 to 23)	18 (12 to 26)
Eastern Europe	6947 (6543 to 7404)	9788 (9244 to 10,364)	5590 (5296 to 5915)	5375 (4716 to 6173)
Belarus	319 (236 to 422)	328 (237 to 448)	158 (113 to 212)	128 (79 to 187)
Estonia	51 (36 to 71)	32 (23 to 44)	10 (7 to 14)	9 (6 to 14)
Latvia	84 (59 to 117)	64 (45 to 88)	22 (15 to 31)	15 (9 to 23)
Lithuania	125 (89 to 173)	122 (84 to 169)	60 (43 to 80)	40 (27 to 57)
Republic of Moldova	170 (119 to 227)	106 (72 to 149)	68 (47 to 94)	46 (31 to 65)
Russian Federation	5071 (4795 to 5360)	7619 (7267 to 8025)	4643 (4398 to 4913)	4035 (3499 to 4756)
Ukraine	1126 (906 to 1402)	1517 (1184 to 1899)	629 (486 to 801)	1103 (738 to 1548)
High-income	15,739 (14,898 to 16,614)	16,408 (15,557 to 17,335)	17,321 (16,285 to 18,337)	17,149 (15,960 to 18,599)
Australasia	291	430	461	479

	(231 to 367)	(355 to 519)	(380 to 557)	(365 to 619)
Australia	195 (144 to 267)	258 (193 to 344)	288 (217 to 373)	346 (243 to 483)
New Zealand	96 (74 to 125)	172 (135 to 212)	173 (135 to 209)	133 (100 to 170)
High-income Asia Pacific	3831 (3356 to 4470)	3319 (2920 to 3781)	3849 (3388 to 4346)	2269 (1963 to 2626)
Brunei Darussalam	2 (1 to 4)	3 (2 to 5)	5 (3 to 7)	4 (2 to 6)
Japan	1957 (1819 to 2095)	1542 (1431 to 1661)	1496 (1373 to 1606)	1333 (1220 to 1444)
Republic of Korea	1764 (1328 to 2280)	1674 (1291 to 2094)	2279 (1841 to 2751)	866 (598 to 1191)
Singapore	108 (79 to 146)	101 (71 to 134)	69 (49 to 91)	66 (46 to 91)
High-income North America	6313 (5904 to 6745)	6992 (6458 to 7471)	7853 (7353 to 8414)	9843 (9055 to 10,791)
Canada	779 (609 to 981)	1112 (886 to 1356)	985 (793 to 1186)	1229 (902 to 1591)
Greenland	20 (12 to 28)	57 (40 to 75)	14 (10 to 18)	9 (5 to 12)
United States of America	5513 (5150 to 5871)	5822 (5369 to 6227)	6854 (6394 to 7330)	8605 (7936 to 9419)
Southern Latin America	1484 (1227 to 1773)	2270 (1913 to 2678)	2761 (2268 to 3261)	2239 (1687 to 2866)
Argentina	998 (773 to 1251)	1708 (1386 to 2084)	2099 (1667 to 2574)	1683 (1183 to 2292)
Chile	409 (301 to 546)	451 (339 to 581)	559 (421 to 707)	477 (332 to 643)
Uruguay	77 (55 to 107)	111 (81 to 153)	102 (73 to 141)	79 (53 to 113)
Western Europe	3821 (3507 to 4144)	3398 (3097 to 3742)	2398 (2160 to 2678)	2319 (2022 to 2670)
Andorra	1 (0 to 1)	0 (0 to 1)	0 (0 to 1)	0 (0 to 0)
Austria	112 (79 to 150)	131 (95 to 175)	74 (52 to 103)	68 (46 to 96)
Belgium	131 (96 to 176)	138 (98 to 183)	99 (70 to 135)	86 (56 to 123)
Cyprus	3 (2 to 6)	3 (1 to 4)	2 (1 to 3)	2 (1 to 4)
Denmark	53 (38 to 75)	26 (18 to 35)	17 (11 to 24)	14 (9 to 22)
Finland	71 (49 to 96)	96 (68 to 131)	60 (42 to 81)	54 (36 to 77)
France	633 (484 to 831)	693 (522 to 887)	551 (410 to 724)	478 (325 to 663)
Germany	742 (580 to 966)	829 (620 to 1,061)	532 (399 to 708)	524 (365 to 737)
Greece	41 (29 to 58)	24 (17 to 35)	21 (14 to 30)	18 (12 to 26)
Iceland	4 (3 to 5)	3 (2 to 3)	2 (1 to 3)	2 (1 to 2)
Ireland	52 (35 to 72)	55 (38 to 77)	34 (23 to 49)	32 (21 to 47)
Israel	77 (53 to 105)	77 (55 to 105)	62 (44 to 85)	66 (45 to 94)
Italy	357 (323 to 395)	252 (227 to 280)	151 (134 to 169)	134 (117 to 156)
Luxembourg	6 (4 to 8)	4 (2 to 5)	3 (2 to 4)	3 (2 to 4)
Malta	2 (1 to 2)	2 (1 to 3)	1 (1 to 1)	1 (0 to 1)
Monaco	0 (0 to 0)	0 (0 to 0)	0 (0 to 0)	0 (0 to 0)
Netherlands	180 (129 to 243)	120 (86 to 167)	94 (69 to 130)	107 (72 to 150)
Norway	143 (127 to 158)	100 (90 to 111)	92 (83 to 102)	81 (71 to 93)
Portugal	207 (145 to 287)	96 (68 to 134)	47 (33 to 65)	36 (24 to 52)
San Marino	0 (0 to 0)	0 (0 to 0)	0 (0 to 0)	0 (0 to 0)
Spain	419 (308 to 561)	207 (147 to 283)	137 (95 to 184)	156 (104 to 227)
Sweden	166 (122 to 213)	154 (117 to 196)	120 (90 to 152)	148 (111 to 189)
Switzerland	104 (75 to 139)	122 (89 to 166)	60 (42 to 83)	65 (44 to 94)
United Kingdom	313	263	235	242

	(286 to 343)	(244 to 284)	(218 to 262)	(220 to 272)
Latin America and Caribbean	15,188 (14,104 to 16,466)	20,969 (19,470 to 22,724)	24,954 (23,143 to 26,918)	21,586 (18,803 to 24,645)
Andean Latin America	2146 (1637 to 2809)	3553 (2799 to 4453)	4396 (3521 to 5464)	3626 (2669 to 4810)
Bolivia (Plurinational State of)	590 (307 to 1027)	934 (489 to 1543)	868 (453 to 1462)	658 (339 to 1136)
Ecuador	726 (519 to 974)	1734 (1276 to 2246)	2653 (2039 to 3365)	2351 (1606 to 3265)
Peru	829 (525 to 1265)	885 (593 to 1274)	874 (561 to 1249)	616 (325 to 1045)
Caribbean	2346 (1868 to 3007)	1746 (1311 to 2401)	1544 (1124 to 2063)	1495 (1002 to 2147)
Antigua and Barbuda	0 (0 to 0)	0 (0 to 0)	0 (0 to 0)	0 (0 to 0)
Bahamas	2 (1 to 3)	2 (1 to 2)	1 (1 to 2)	1 (1 to 2)
Barbados	4 (3 to 6)	2 (1 to 3)	2 (1 to 2)	1 (1 to 2)
Belize	7 (4 to 10)	8 (6 to 12)	5 (4 to 8)	4 (3 to 7)
Bermuda	1 (0 to 1)	0 (0 to 1)	0 (0 to 0)	0 (0 to 0)
Cuba	828 (632 to 1030)	406 (306 to 533)	217 (157 to 292)	150 (100 to 217)
Dominica	1 (1 to 2)	1 (1 to 2)	1 (1 to 1)	1 (0 to 1)
Dominican Republic	203 (126 to 307)	189 (120 to 293)	230 (138 to 355)	193 (99 to 332)
Grenada	2 (1 to 3)	1 (1 to 2)	1 (1 to 1)	1 (0 to 1)
Guyana	144 (92 to 213)	76 (48 to 108)	114 (70 to 162)	58 (34 to 93)
Haiti	750 (396 to 1335)	794 (418 to 1363)	749 (383 to 1284)	894 (463 to 1554)
Jamaica	17 (12 to 25)	15 (11 to 23)	33 (22 to 48)	25 (16 to 39)
Puerto Rico	38 (26 to 53)	23 (16 to 32)	17 (12 to 25)	9 (6 to 14)
Saint Kitts and Nevis	1 (1 to 2)	1 (0 to 1)	0 (0 to 1)	0 (0 to 0)
Saint Lucia	2 (1 to 3)	2 (2 to 3)	2 (1 to 3)	1 (1 to 2)
Saint Vincent and the Grenadines	3 (2 to 4)	2 (1 to 3)	2 (1 to 2)	1 (1 to 1)
Suriname	75 (32 to 115)	23 (13 to 36)	71 (46 to 105)	49 (28 to 80)
Trinidad and Tobago	186 (130 to 251)	139 (97 to 187)	46 (31 to 64)	54 (34 to 81)
United States Virgin Islands	2 (1 to 4)	1 (1 to 2)	1 (0 to 1)	1 (0 to 1)
Central Latin America	6009 (5435 to 6654)	10,453 (9590 to 11,405)	13,084 (12,110 to 14,140)	10,843 (9101 to 12,792)
Colombia	1102 (845 to 1392)	3098 (2491 to 3763)	3201 (2574 to 3883)	2335 (1515 to 3319)
Costa Rica	94 (66 to 126)	127 (91 to 176)	152 (109 to 203)	113 (73 to 170)
El Salvador	1086 (773 to 1460)	882 (667 to 1130)	817 (595 to 1075)	382 (232 to 589)
Guatemala	208 (136 to 292)	421 (307 to 578)	759 (569 to 976)	705 (451 to 1039)
Honduras	152 (85 to 258)	94 (46 to 164)	68 (35 to 122)	46 (23 to 77)
Mexico	1900 (1715 to 2072)	3895 (3570 to 4252)	6422 (5910 to 7014)	5930 (5059 to 6976)
Nicaragua	553 (368 to 780)	678 (500 to 886)	632 (480 to 817)	446 (279 to 660)
Panama	89 (58 to 128)	79 (55 to 113)	94 (66 to 133)	83 (53 to 123)
Venezuela (Bolivarian Republic of)	825 (623 to 1069)	1178 (915 to 1486)	939 (741 to 1185)	804 (516 to 1200)
Tropical Latin America	4688 (4216 to 5221)	5216 (4718 to 5742)	5931 (5377 to 6525)	5623 (4828 to 6516)
Brazil	4365 (3906 to 4892)	4610 (4183 to 5059)	5303 (4774 to 5844)	5038 (4352 to 5844)
Paraguay	323 (212 to 468)	606 (440 to 821)	627 (455 to 820)	585 (365 to 899)
North Africa and Middle East	17,567 (13,965 to 21,757)	17,750 (14,888 to 21,417)	13,205 (10,938 to 16,165)	10,396 (8064 to 13,452)
Afghanistan	149	206	273	265

	(74 to 274)	(107 to 358)	(147 to 451)	(139 to 457)
Algeria	2350 (1148 to 4244)	1810 (932 to 3064)	980 (514 to 1643)	824 (420 to 1440)
Bahrain	4 (2 to 7)	8 (5 to 12)	7 (5 to 11)	6 (4 to 10)
Egypt	3534 (1765 to 6090)	2564 (1326 to 4675)	1850 (923 to 3319)	1750 (877 to 3328)
Iran (Islamic Republic of)	5743 (4688 to 6858)	6768 (5813 to 7812)	2933 (2514 to 3419)	1603 (1319 to 1941)
Iraq	315 (159 to 577)	457 (242 to 813)	398 (221 to 653)	243 (130 to 411)
Jordan	62 (36 to 100)	70 (41 to 114)	30 (19 to 45)	57 (32 to 95)
Kuwait	14 (10 to 21)	13 (9 to 19)	14 (10 to 21)	15 (10 to 22)
Lebanon	73 (39 to 129)	73 (37 to 126)	45 (23 to 78)	43 (21 to 76)
Libya	138 (71 to 234)	125 (65 to 210)	92 (49 to 159)	73 (39 to 123)
Morocco	1367 (710 to 2416)	1449 (746 to 2625)	1077 (570 to 1807)	784 (399 to 1400)
Oman	17 (9 to 30)	16 (9 to 27)	12 (6 to 19)	11 (6 to 18)
Palestine	28 (15 to 46)	32 (19 to 50)	32 (19 to 50)	32 (18 to 52)
Qatar	7 (4 to 11)	10 (5 to 16)	9 (5 to 15)	12 (7 to 21)
Saudi Arabia	96 (48 to 183)	91 (53 to 145)	75 (46 to 117)	53 (27 to 93)
Sudan	1210 (565 to 2216)	1571 (699 to 2992)	1647 (746 to 3222)	1601 (780 to 3047)
Syrian Arab Republic	105 (60 to 174)	101 (62 to 158)	95 (57 to 151)	69 (40 to 114)
Tunisia	347 (183 to 599)	291 (152 to 488)	167 (86 to 298)	121 (60 to 218)
Turkey	1056 (559 to 1761)	809 (437 to 1368)	2246 (1595 to 3036)	1396 (844 to 2112)
United Arab Emirates	13 (6 to 22)	17 (9 to 28)	14 (7 to 23)	12 (6 to 22)
Yemen	927 (440 to 1766)	1255 (585 to 2306)	1197 (575 to 2159)	1415 (696 to 2506)
South Asia	199,023 (149,879 to 251,428)	227,408 (178,277 to 277,823)	153,162 (119,342 to 185,637)	135,575 (103,123 to 170,351)
Bangladesh	12,270 (6347 to 19,994)	11,105 (6946 to 17,117)	8286 (5183 to 12,488)	3851 (2015 to 6714)
Bhutan	13 (2 to 28)	16 (8 to 29)	11 (6 to 20)	9 (4 to 16)
India	183,056 (135,235 to 232,190)	211,114 (165,321 to 258,370)	137,723 (106,932 to 167,891)	123,287 (93,189 to 156,772)
Nepal	720 (364 to 1316)	598 (298 to 1090)	718 (369 to 1335)	484 (240 to 886)
Pakistan	2965 (1712 to 4750)	4575 (2786 to 7085)	6423 (3965 to 10,050)	7945 (4715 to 13,206)
Southeast Asia, East Asia, and Oceania	164,063 (141,717 to 190,914)	166,900 (148,763 to 186,175)	48,215 (43,922 to 52,903)	26,111 (22,975 to 29,873)
East Asia	141,183 (119,853 to 168,606)	150,657 (133,661 to 169,319)	37,386 (33,845 to 41,650)	16,901 (14,398 to 19,706)
China	139,104 (117,612 to 166,451)	149,129 (132,122 to 167,848)	36,291 (32,822 to 40,411)	16,298 (13,910 to 19,015)
Democratic People's Republic of Korea	1765 (842 to 3153)	1244 (629 to 2180)	877 (464 to 1495)	489 (256 to 831)
Taiwan, Province of China	314 (222 to 415)	285 (210 to 376)	218 (155 to 293)	115 (76 to 171)
Oceania	275 (201 to 376)	341 (256 to 453)	281 (209 to 384)	299 (216 to 403)
American Samoa	2 (1 to 3)	2 (1 to 3)	2 (1 to 3)	2 (1 to 3)
Cook Islands	2 (1 to 4)	1 (1 to 2)	1 (0 to 1)	1 (0 to 1)
Fiji	93 (49 to 154)	124 (75 to 192)	54 (33 to 78)	66 (37 to 110)
Guam	11 (7 to 17)	16 (10 to 23)	13 (9 to 20)	17 (10 to 25)
Kiribati	9 (5 to 14)	11 (6 to 18)	11 (6 to 18)	10 (5 to 18)
Marshall Islands	6 (4 to 11)	9 (5 to 14)	7 (4 to 12)	6 (3 to 9)
Micronesia (Federated States of)	16 (8 to 27)	15 (8 to 25)	12 (6 to 19)	8 (1 to 14)
Nauru	1	2	2	1

	(1 to 2)	(1 to 3)	(1 to 3)	(1 to 2)
Niue	0<br>(0 to 0)	0<br>(0 to 0)	0<br>(0 to 0)	0<br>(0 to 0)
Northern Mariana Islands	2<br>(1 to 3)	1<br>(1 to 2)	1<br>(1 to 2)	1<br>(1 to 2)
Palau	1<br>(1 to 2)	1<br>(1 to 2)	1<br>(1 to 2)	1<br>(0 to 1)
Papua New Guinea	72<br>(36 to 130)	87<br>(43 to 157)	111<br>(58 to 196)	118<br>(61 to 202)
Samoa	17<br>(9 to 29)	15<br>(8 to 25)	13<br>(7 to 22)	13<br>(6 to 23)
Solomon Islands	7<br>(3 to 12)	9<br>(4 to 15)	9<br>(5 to 17)	11<br>(5 to 18)
Tokelau	0<br>(0 to 0)	0<br>(0 to 0)	0<br>(0 to 0)	0<br>(0 to 0)
Tonga	4<br>(2 to 8)	6<br>(3 to 9)	4<br>(2 to 7)	3<br>(2 to 5)
Tuvalu	1<br>(1 to 2)	1<br>(1 to 2)	1<br>(0 to 2)	1<br>(0 to 1)
Vanuatu	13<br>(7 to 23)	22<br>(12 to 37)	25<br>(13 to 41)	27<br>(15 to 45)
Southeast Asia	22,605<br>(18,030 to 27,627)	15,901<br>(13,027 to 18,939)	10,548<br>(8926 to 12,401)	8911<br>(7312 to 10,705)
Cambodia	593<br>(303 to 1050)	601<br>(311 to 1047)	325<br>(174 to 550)	225<br>(119 to 374)
Indonesia	6367<br>(4573 to 8631)	4878<br>(3604 to 6373)	3854<br>(2991 to 4893)	3208<br>(2503 to 4103)
Lao People's Democratic Republic	602<br>(303 to 1075)	506<br>(270 to 878)	353<br>(177 to 611)	196<br>(102 to 344)
Malaysia	133<br>(72 to 224)	111<br>(68 to 170)	69<br>(42 to 105)	75<br>(41 to 132)
Maldives	6<br>(3 to 11)	3<br>(2 to 6)	1<br>(1 to 3)	1<br>(1 to 2)
Mauritius	184<br>(138 to 236)	100<br>(74 to 130)	49<br>(36 to 67)	52<br>(35 to 75)
Myanmar	1794<br>(939 to 3242)	1658<br>(846 to 2903)	996<br>(513 to 1733)	684<br>(359 to 1137)
Philippines	2325<br>(1961 to 2769)	1980<br>(1678 to 2299)	1878<br>(1634 to 2185)	1803<br>(1451 to 2197)
Seychelles	1<br>(0 to 1)	0<br>(0 to 1)	0<br>(0 to 0)	0<br>(0 to 0)
Sri Lanka	3884<br>(2649 to 5360)	1863<br>(1416 to 2409)	942<br>(650 to 1274)	987<br>(569 to 1561)
Thailand	4123<br>(2299 to 6736)	2085<br>(1287 to 3191)	1042<br>(703 to 1524)	665<br>(376 to 1029)
Timor-Leste	51<br>(22 to 95)	48<br>(23 to 88)	36<br>(15 to 66)	36<br>(16 to 63)
Viet Nam	2511<br>(1302 to 4390)	2046<br>(1107 to 3399)	988<br>(578 to 1651)	965<br>(506 to 1630)
Sub-Saharan Africa	21,473<br>(16,593 to 27,566)	26,024<br>(20,237 to 32,693)	32,091<br>(25,512 to 38,419)	32,636<br>(25,570 to 40,879)
Central Sub-Saharan Africa	2374<br>(1454 to 3802)	3142<br>(1809 to 5029)	4114<br>(2471 to 6280)	4026<br>(2504 to 6151)
Angola	497<br>(228 to 914)	680<br>(289 to 1257)	872<br>(442 to 1549)	883<br>(439 to 1532)
Central African Republic	133<br>(63 to 240)	162<br>(71 to 290)	237<br>(117 to 407)	233<br>(117 to 446)
Congo	81<br>(27 to 157)	115<br>(48 to 204)	130<br>(68 to 222)	112<br>(56 to 199)
Democratic Republic of the Congo	1616<br>(811 to 2943)	2131<br>(972 to 3897)	2818<br>(1447 to 4878)	2756<br>(1433 to 4673)
Equatorial Guinea	18<br>(9 to 35)	25<br>(10 to 46)	26<br>(11 to 49)	19<br>(7 to 39)
Gabon	29<br>(11 to 57)	30<br>(13 to 53)	32<br>(15 to 57)	23<br>(10 to 41)
Eastern Sub-Saharan Africa	11,939<br>(8833 to 15,859)	13,468<br>(10,305 to 17,503)	16,248<br>(12,839 to 20,350)	14,827<br>(11,384 to 18,965)
Burundi	414<br>(209 to 724)	504<br>(249 to 883)	604<br>(314 to 1062)	686<br>(362 to 1198)
Comoros	24<br>(3 to 53)	29<br>(9 to 57)	20<br>(8 to 39)	26<br>(11 to 49)
Djibouti	20<br>(8 to 36)	30<br>(13 to 56)	30<br>(13 to 56)	35<br>(15 to 67)
Eritrea	140<br>(63 to 266)	182<br>(88 to 345)	271<br>(137 to 493)	278<br>(140 to 514)
Ethiopia	4484<br>(2711 to 6975)	5665<br>(3648 to 8148)	6453<br>(4315 to 9185)	4435<br>(2812 to 6881)
Kenya	792<br>(544 to 1067)	713<br>(507 to 988)	1050<br>(785 to 1378)	1048<br>(781 to 1402)
Madagascar	1228	883	1133	1131

	(634 to 2155)	(479 to 1489)	(560 to 1948)	(542 to 2008)
Malawi	607<br>(284 to 1114)	617<br>(336 to 1092)	551<br>(299 to 944)	597<br>(302 to 1085)
Mozambique	769<br>(390 to 1374)	631<br>(321 to 1154)	1050<br>(523 to 1788)	917<br>(443 to 1692)
Rwanda	599<br>(294 to 1090)	670<br>(334 to 1150)	453<br>(224 to 818)	445<br>(219 to 805)
Somalia	372<br>(181 to 682)	526<br>(232 to 981)	960<br>(489 to 1679)	1219<br>(650 to 2150)
South Sudan	194<br>(92 to 356)	219<br>(91 to 423)	276<br>(131 to 518)	339<br>(161 to 655)
Uganda	407<br>(130 to 832)	1018<br>(495 to 1922)	1003<br>(501 to 1798)	1140<br>(536 to 2115)
United Republic of Tanzania	1340<br>(657 to 2367)	1222<br>(660 to 2091)	1729<br>(948 to 3028)	1991<br>(974 to 3607)
Zambia	542<br>(265 to 973)	548<br>(267 to 993)	652<br>(354 to 1108)	529<br>(272 to 924)
Southern Sub-Saharan Africa	503<br>(375 to 674)	659<br>(525 to 802)	701<br>(546 to 907)	382<br>(218 to 610)
Botswana	14<br>(7 to 27)	17<br>(8 to 32)	19<br>(9 to 34)	20<br>(10 to 36)
Eswatini	10<br>(5 to 18)	11<br>(6 to 20)	12<br>(6 to 23)	10<br>(5 to 18)
Lesotho	24<br>(11 to 48)	20<br>(10 to 36)	23<br>(11 to 46)	22<br>(11 to 43)
Namibia	12<br>(4 to 24)	14<br>(6 to 25)	14<br>(7 to 25)	13<br>(5 to 23)
South Africa	264<br>(200 to 345)	528<br>(422 to 645)	440<br>(337 to 574)	44<br>(19 to 85)
Zimbabwe	178<br>(84 to 329)	69<br>(24 to 142)	192<br>(92 to 350)	272<br>(122 to 499)
Western Sub-Saharan Africa	6656<br>(4606 to 8945)	8756<br>(6178 to 11,653)	11,028<br>(8158 to 14,351)	13,401<br>(9528 to 17,937)
Benin	188<br>(97 to 337)	262<br>(129 to 449)	359<br>(167 to 648)	447<br>(202 to 921)
Burkina Faso	418<br>(204 to 755)	570<br>(303 to 950)	717<br>(393 to 1167)	1005<br>(491 to 1827)
Cabo Verde	5<br>(3 to 9)	6<br>(3 to 11)	4<br>(2 to 6)	3<br>(2 to 6)
Cameroon	364<br>(180 to 650)	662<br>(339 to 1164)	927<br>(472 to 1649)	1044<br>(506 to 1904)
Chad	238<br>(119 to 437)	397<br>(202 to 721)	617<br>(308 to 1056)	864<br>(416 to 1536)
Côte d'Ivoire	393<br>(182 to 693)	610<br>(275 to 1085)	702<br>(331 to 1233)	726<br>(336 to 1385)
Gambia	33<br>(16 to 60)	49<br>(24 to 85)	59<br>(30 to 103)	58<br>(26 to 104)
Ghana	396<br>(207 to 703)	502<br>(281 to 815)	666<br>(385 to 1119)	554<br>(278 to 1006)
Guinea	293<br>(145 to 537)	387<br>(191 to 685)	527<br>(278 to 938)	643<br>(299 to 1125)
Guinea-Bissau	56<br>(27 to 101)	74<br>(36 to 127)	77<br>(37 to 138)	64<br>(33 to 109)
Liberia	99<br>(51 to 183)	125<br>(62 to 221)	165<br>(86 to 293)	168<br>(79 to 300)
Mali	498<br>(256 to 906)	621<br>(310 to 1079)	775<br>(397 to 1349)	1015<br>(462 to 1886)
Mauritania	73<br>(36 to 128)	95<br>(49 to 165)	97<br>(49 to 170)	99<br>(44 to 193)
Niger	409<br>(200 to 745)	503<br>(255 to 900)	640<br>(317 to 1150)	1024<br>(474 to 1962)
Nigeria	2557<br>(1310 to 4205)	3136<br>(1555 to 5237)	3723<br>(2009 to 6012)	4604<br>(2317 to 7899)
Sao Tome and Principe	2<br>(1 to 3)	2<br>(1 to 4)	2<br>(1 to 3)	1<br>(1 to 3)
Senegal	347<br>(172 to 600)	379<br>(185 to 679)	464<br>(232 to 830)	491<br>(239 to 885)
Sierra Leone	132<br>(67 to 229)	201<br>(99 to 359)	323<br>(159 to 565)	382<br>(173 to 696)
Togo	154<br>(82 to 267)	175<br>(84 to 304)	184<br>(93 to 318)	206<br>(98 to 396)
Africa	30,594<br>(24,020 to 38,555)	33,923<br>(27,319 to 41,463)	37,940<br>(30,747 to 45,119)	37,827<br>(30,328 to 46,527)
America	22,846<br>(21,562 to 24,223)	30,089<br>(28,456 to 31,953)	35,483<br>(33,560 to 37,560)	33,598<br>(30,481 to 37,227)
Asia	377,648<br>(322,394 to 441,570)	411,524<br>(357,353 to 471,446)	215,942<br>(180,915 to 249,959)	173,553<br>(140,003 to 210,122)
Europe	14,832<br>(13,959 to 15,771)	16,134<br>(15,330 to 17,024)	11,601<br>(10,742 to 12,513)	10,012<br>(8982 to 11,191)
